# Supplementary material for: Structural basis of the membrane intramolecular transacylase reaction responsible for lyso-form lipoprotein synthesis
Source: Nat Commun. 2021 Jul 12;12:4254. doi: 10.1038/s41467-021-24475-0 (PMC8275575; doi:10.1038/s41467-021-24475-0)
Supplement: Supplementary file 1 — Supplementary Information [file 41467_2021_24475_MOESM1_ESM.pdf]

Supplementary Information

for

Structural basis of the membrane intramolecular transacylase action responsible for lyso-form lipoprotein synthesis

Samir Olatunji[1], Katherine Bowen[2], Chia-Ying Huang[3], Dietmar Weichert[1], Warispreet Singh[4^], Irina G. Tikhonova[4], Eoin M. Scanlan[2], Vincent Olieric[3], Martin Caffrey[1]*

* Correspondence and requests for materials should be addressed to M.C. (email: martin.caffrey@tcd.ie)

Supplementary Methods
Supplementary Figures 1 to 53
Supplementary Tables 1 to 6
Supplementary References

Supplementary Methods

Lipopeptide synthesis and characterization

Abbreviations

Abz	2-aminobenzoyl
Ar	aromatic
calcd.	calculated
d	doublet
DAG	diacylglyceryl
dd	doublet of doublets
DIPEA	*N,N*-diisopropylethylamine
DMAP	4-(dimethylamino)pyridine
DMF	*N,N*-dimethylformamide
EDC·HCl	*N*-(3-dimethylaminopropyl)-*N'*-ethylcarbodiimide hydrochloride
Equiv.	equivalents
ESI	electrospray ionisation
EtOAc	ethyl acetate
Fmoc	fluorenylmethyloxycarbonyl
HRMS	high resolution mass spectrometry
IR	infrared
m	multiplet
MALDI	matrix assisted laser desorption ionization
MeOH	methanol
NMM	*N*-methylmorpholine
NMR	nuclear magnetic resonance
Mtt	4-methyltrityl
Pal	palmitic
Pam	pamitoyl
ppm	parts per million
PyBOP	benzotriazol-1-yl-oxytripyrrolidinophosphonium hexafluorophosphate
qC	quaternary carbon
Q-ToF	quadrupole time-of-flight
s	singlet
SPPS	solid phase peptide synthesis
t	triplet
TES	triethylsilane
TFA	trifluoroacetic acid
TLC	thin layer chromatography
R_f	retention factor

Instrumental and General Considerations

Proton nuclear magnetic resonance (^{1}H NMR) and carbon nuclear magnetic resonance (^{13}C NMR) spectra were recorded on a 400 MHz Bruker Avance spectrometer (^{1}H, 400.13 MHz; ^{13}C, 100.6 MHz) or a 600 MHz Bruker Avance II spectrometer (^{1}H, 600.13 MHz; ^{13}C, 150.6 MHz). Resonances δ, are in parts per million (ppm) calibrated using residual nondeuterated solvent (^{1}H NMR) or the deuterated solvent (^{13}C NMR) as internal reference standards. Infrared (IR) spectra were recorded on a Perkin

Elmer spectrometer. Mass spectrometry analysis was performed with a Waters Premier quadrupole time-of-flight (Q-ToF) mass spectrometer equipped with Z–spray electrospray ionization (ESI) and matrix assisted laser desorption ionization (MALDI) sources. Silica gel Florisil (200 mesh; Aldrich) was used for column chromatography. Thin-layer chromatography (TLC) was performed using Merck 60 F254 silica gel plates (pre-coated, 0.2 mm thick) and visualised by UV light (λ_{max} = 254 nm) and ammonium molybdate staining (ammonium molybdate (0.26 M) in aq. H_2SO_4 (1 M)). Unless otherwise stated, protected amino acids for peptide synthesis and all other reagents were purchased from an industrial supplier. In the following section, the peptides and the intermediates used in their syntheses are identified in shorthand by emboldened arabic numerals.

Lit substrates **1-3** (FP2: **1**, (*S*)-DAG-FP2: **2** and dFP2: **3**) were synthesized using manual Fmoc/*t*Bu SPPS performed in polypropylene syringe reaction vessels (10 mL; Torviq, MI, USA). All SPPS reactions were carried out at room temperature (19.5 – 20.5 °C) under continuous agitation.

The protected amino acid 2-((*tert*-butoxycarbonyl)amino)benzoic acid (Boc-Abz-OH) and the modified cysteine building blocks *tert*-butyl-*N*-(((9*H*-fluoren-9-yl)methoxy)carbonyl)-*S*-((*R*)-2,3-dihydroxypropyl)-L-cysteinate (Fmoc-Cys((*R*)-2,3-dihydroxypropyl)-OH and *N*-(((9*H*-fluoren-9-yl)methoxy)carbonyl)-*S*-((*R*)-2,3-bis(palmitoyloxy)propyl)-L-cysteine (Fmoc-Cys((*R*)-2,3-bis(palmitoyloxy)propyl)-OH) were synthesized as previously reported[1].

Fmoc-Cys((*S*)-2,3-bis(palmitoyloxy)propyl)-OH (**4**) used in the synthesis of peptide **2** and Fmoc-Cys((*R*)-2-((hexadecanoyl-d_{31})oxy)-3-(palmitoyloxy)propyl)-OH (**5**) used in the synthesis of peptide **3** were synthesized as described below and as shown in Supplementary Fig. 37 and Supplementary Fig. 38, respectively. The SPPS of peptides **1**-**3** is outlined in Supplementary Fig. 39. The NMR spectra of novel compounds are shown in Supplementary Figs. 40-53.

Synthesis of Modified Amino Acids for Peptide Synthesis

<u>Synthesis of Fmoc-Cys((*S*)-Pam)$_2$-OH (4)</u>

Compounds **4**, **6** and **7** were synthesized using an adapted literature procedure as shown in Supplementary Fig. 37. Spectral data were in good agreement with the literature[2].

***tert*-Butyl-*N*-(((9*H*-fluoren-9-yl)methoxy)carbonyl)-*S*-((*S*)-2,3-dihydroxypropyl)-L-cysteinate (6)**

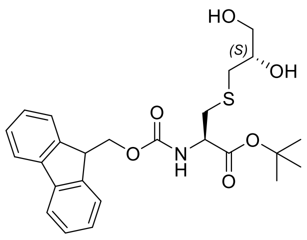

To a solution of (Fmoc-Cys-O*t*Bu)$_2$ (0.600 g, 0.750 mmol) in anhydrous THF (10 mL) under argon was added PBu_3 (0.187 mL, 0.750 mmol). The reaction was stirred at room temperature for 5 min followed by the addition of H_2O (2 mL). The solution was stirred at room temperature for 4 h at which time TLC analysis indicated complete consumption of (Fmoc-Cys-O*t*Bu)$_2$ disulfide (R_f = 0.31; EtOAc:n-hexane 1:4) and formation of thiol (R_f = 0.46; EtOAc:n-hexane 1:4). The solvents were evaporated under reduced pressure followed by addition of anhydrous CH_2Cl_2 (10 mL) under argon. To this solution was added (*S*)-(-)-glycidol (1.11 mL, 15.0 mmol) and DIPEA (0.260 mL, 1.50 mmol) under argon. The reaction was stirred at 40 °C for 16 h. The reaction was cooled to room temperature and washed with H_2O (3 x 15 mL) and sat. aq. NaCl solution (3 x 15 mL) and the organic

layer was dried over $MgSO_4$. The solvent was removed *in vacuo* and the product was purified using silica column chromatography (EtOAc:n-hexane 1:1 – 4:1) to afford **6** as a viscous, colourless oil (0.385 g, 51%).

TLC (EtOAc:n-hexane 4:1) R_f = 0.36; ^{1}H NMR (600 MHz, $CDCl_3$) δ 7.77 (d, *J* = 7.4 Hz, 2H, Fmoc-Ar), 7.61 (d, *J* = 7.4 Hz, 2H, Fmoc-Ar), 7.41 (t, *J* = 7.4 Hz, 2H, Fmoc-Ar), 7.32 (t, *J* = 7.4 Hz, 2H, Fmoc-Ar), 5.70 (d, *J* = 7.7 Hz, 1H, NH), 4.53 – 4.47 (m, 1H, Cys-αCH), 4.47 – 4.34 (m, 2H, Fmoc-CH_2), 4.27 – 4.24 (t, *J* = 7.3 Hz, 1H, Fmoc-CH), 3.83 – 3.78 (m, 1H, S-glyceryl-CH), 3.69 (dd, *J* = 11.1, 3.2 Hz, 1H, S-glyceryl-OC$\underline{H}_a H_b$), 3.56 (dd, *J* = 11.1, 5.6 Hz, 1H, S-glyceryl-OC$H_a \underline{H}_b$), 3.05 – 2.99 (m, 1H, Cys-βC$\underline{H}_a H_b$), 2.97 – 2.92 (m, 1H, Cys-βC$H_a \underline{H}_b$), 2.82 – 2.76 (m, 1H, S-glyceryl-C$\underline{H}_a H_b$), 2.70 – 2.64 (m, 1H, S-glyceryl-C$H_a \underline{H}_b$), 1.50 (s, 9H, *t*Bu-CH_3) ppm; ^{13}C NMR (151 MHz, $CDCl_3$) δ 169.8 (Cys C=O), 156.2 (Fmoc C=O), 144.0 (Fmoc-qC), 141.5 (Fmoc-qC), 127.9 (Fmoc-Ar-CH), 127.3 (Fmoc-Ar-CH), 125.3 (Fmoc-Ar-CH), 120.2 (Fmoc-Ar-CH), 83.4 (*t*Bu-qC), 70.4 (S-glyceryl-CH), 67.4 (Fmoc-CH_2), 65.3 (S-glyceryl-OCH_2), 54.6 (Cys-αCH), 47.3 (Fmoc-CH), 36.9 (S-glyceryl-CH_2), 36.1 (Cys-βCH_2), 28.2 (*t*Bu-CH_3) ppm; *m/z* HRMS (ESI$^+$) calcd. $C_{25}H_{31}NNaO_6S$ = 496.1764 $(M + Na)^+$. Found = 496.1772.

(*S*)-3-(((*R*)-2-((((9*H*-Fluoren-9-yl)methoxy)carbonyl)amino)-3-(*tert*-butoxy)-3-oxopropyl)thio)propane-1,2-diyl dipalmitate (7)

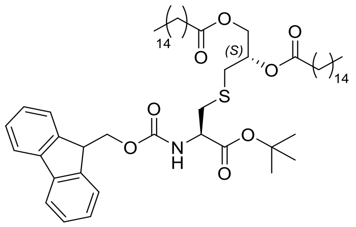

To a solution of palmitic acid (0.387 g, 1.51 mmol) in anhydrous CH_2Cl_2 (10 mL) under argon at 0 °C was added *N*-(3-dimethylaminopropyl)-*N'*-ethylcarbodiimide hydrochloride (EDC·HCl) (0.270 g, 1.51 mmol) and 4-(dimethylamino)pyridine (DMAP) (0.015 g, 0.12 mmol). The reaction was stirred at 0 °C for 1 h. Diol **6** (0.305 g, 0.605 mmol) was dissolved in anhydrous CH_2Cl_2 (10 mL) and added to the reaction which was stirred at room temperature under argon for 16 h. The solvent was removed *in vacuo* and the product was purified using silica column chromatography (n-hexane - EtOAc:n-hexane 1:9) to yield **7** as a white solid (0.395 g, 69%).

TLC (EtOAc:n-hexane 1:4) R_f = 0.60; ^{1}H NMR (600 MHz, $CDCl_3$) δ 7.76 (d, *J* = 7.3 Hz, 2H, Fmoc-Ar), 7.62 (d, *J* = 7.3 Hz, 2H, Fmoc-Ar), 7.40 (t, *J* = 7.3 Hz, 2H, Fmoc-Ar), 7.31 (t, *J* = 7.3 Hz, 2H, Fmoc-Ar), 5.67 (d, *J* = 7.3 Hz, 1H, NH), 5.18 – 5.13 (m, 1H, S-glyceryl-CH), 4.54 – 4.48 (m, 1H, Cys-αCH), 4.43 – 4.30 (m, 3H, Fmoc-CH_2, S-glyceryl-OC$\underline{H}_a H_b$), 4.24 (t, *J* = 7.1 Hz, 1H, Fmoc-CH), 4.15 (dd, *J* = 11.9, 5.9 Hz, 1H, S-glyceryl-OC$H_a \underline{H}_b$), 3.08 (dd, *J* = 13.5, 4.2 Hz, 1H, Cys-βC$\underline{H}_a H_b$), 3.02 (dd, *J* = 13.5, 5.1 Hz, 1H, Cys-βC$H_a \underline{H}_b$), 2.82 – 2.72 (m, 2H, S-glyceryl-CH_2), 2.28 (t, *J* = 7.3 Hz, 4H, Pal-αCH_2 x2), 1.61 – 1.56 (m, 4H, Pal-CH_2 x2), 1.49 (s, 9H, *t*Bu-CH_3), 1.32 – 1.22 (m, 48H, Pal-CH_2), 0.88 (t, *J* = 6.8 Hz, 6H, Pal-CH_3 x 2) ppm; ^{13}C NMR (151 MHz, $CDCl_3$) δ 173.5 (Pal C=O), 173.2 (Pal C=O), 169.6 (Cys C=O), 155.8 (Fmoc C=O), 144.0 (Fmoc-qC), 141.4 (Fmoc-qC), 127.9 (Fmoc-Ar-CH), 127.2 (Fmoc-Ar-CH), 125.3 (Fmoc-Ar-CH), 120.1 (Fmoc-Ar-CH), 83.2 (*t*Bu-qC), 70.5 (S-glycery-CH), 67.4 (Fmoc-CH_2), 63.6 (S-glyceryl-OCH_2), 54.5 (Cys-αCH), 47.3 (Fmoc-CH), 35.6 (Cys-βCH_2), 34.4, 34.2, 33.4, 32.1, 29.9, 29.8, 29.7, 29.6, 29.5, 29.4, 29.3, 29.2 (Pal-CH_2), 28.1 (*t*Bu-CH_3), 25.0 (Pal-CH_2), 22.8 (Pal-CH_2), 14.3 (Pal-CH_3) ppm; *m/z* HRMS (ESI$^+$) calcd. $C_{57}H_{91}NNaO_8S$ = 972.6358 $(M + Na)^+$. Found = 972.6359.

N-(((9H-Fluoren-9-yl)methoxy)carbonyl)-S-((S)-2,3-bis(palmitoyloxy)propyl)-L-cysteine (4)

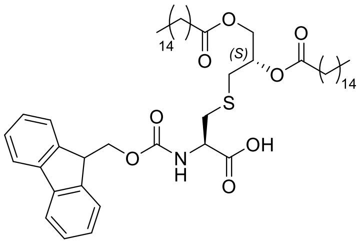

To a solution of compound **7** (0.390 g, 0.411 mmol) in CH_2Cl_2 (3 mL) was added TES (0.5 mL, 3.13 mmol) and TFA (7 mL). The reaction was stirred at room temperature for 4 h. The solvents were removed *in vacuo* and the product recrystallized from a minimum amount of CH_2Cl_2 (5 mL) layered with MeOH (50 mL) for 16 h. The obtained white solid was filtered and dried *in vacuo* to yield **4** as a white solid (0.286 g, 78%).

TLC (CH_2Cl_2:MeOH 9:1) R_f = 0.53; ^{1}H NMR (600 MHz, $CDCl_3$) δ 7.76 (d, *J* = 7.5 Hz, 2H, Fmoc-Ar), 7.63 – 7.59 (m, 2H, Fmoc-Ar), 7.40 (t, *J* = 7.5 Hz, 2H, Fmoc-Ar), 7.31 (t, *J* = 7.5 Hz, 2H, Fmoc-Ar), 5.76 (d, *J* = 7.4 Hz, 1H, NH), 5.19 – 5.14 (m, 1H, S-glyceryl-CH), 4.71 – 4.66 (m, 1H, Cys-αCH), 4.42 – 4.36 (m, 3H, S-glyceryl-OC$\underline{H}_a$$H_b$, Fmoc-$CH_2$), 4.24 (t, *J* = 7.0 Hz, 1H, Fmoc-CH), 4.13 (dd, *J* = 11.8, 6.1 Hz, 1H, S-glyceryl-OC$H_a$$\underline{H}_b$), 3.17 (dd, *J* = 13.4, 3.3 Hz, 1H, Cys-βC$\underline{H}_a$$H_b$), 3.10 (dd, *J* = 13.4, 4.3 Hz, 1H, Cys-βC$H_a$$\underline{H}_b$), 2.79 (dd, *J* = 13.8, 6.5 Hz, 1H, S-glyceryl-C$\underline{H}_a$$H_b$), 2.73 (dd, *J* = 13.8, 5.9 Hz, 1H, S-glyceryl-C$H_a$$\underline{H}_b$), 2.33 – 2.28 (m, 4H, Pal-αCH_2 x2), 1.63 – 1.56 (m, 2H, Pal-CH_2 x2), 1.35 – 1.21 (m, 48H, Pal-CH_2), 0.88 (t, *J* = 6.9 Hz, 6H, Pal-CH_3 x 2) ppm; ^{13}C NMR (151 MHz, $CDCl_3$) δ 173.8 (Pal C=O), 173.6 (Pal C=O), 172.8 (Cys C=O), 156.1 (Fmoc-C=O), 143.9 (Fmoc-qC), 141.5 (Fmoc-qC), 127.9 (Fmoc-Ar-CH), 127.3 (Fmoc-Ar-CH), 125.3 (Fmoc-Ar-CH), 120.2 (Fmoc-Ar-CH), 70.5 (*S*-glycery-CH), 67.6 (Fmoc-CH_2), 63.8 (*S*-glyceryl-*O*CH_2), 53.5 (Cys-αCH), 47.2 (Fmoc-CH), 34.5 (Cys-βCH_2), 34.3 (Pal-CH_2), 33.0 (*S*-glycery-CH_2), 32.1, 29.9, 29.8, 29.8, 29.7, 29.5, 29.4, 29.3, 25.1, 22.8 (Pal-CH_2), 14.3 (Pal-CH_3); *m/z* HRMS (ESI$^+$) calcd. $C_{53}H_{83}NNaO_8S$ = 916.5732 (M + Na)$^+$. Found = 916.5727.

<u>Synthesis of Fmoc-Cys(Pam-d_{31},Pam)-OH (5)</u>

Compounds **5**, **8** and **9** were synthesised as shown in Supplementary Fig. 38.

(R)-3-(((R)-2-((((9H-Fluoren-9-yl)methoxy)carbonyl)amino)-3-(tert-butoxy)-3-oxopropyl)thio)-2-hydroxypropyl palmitate (8)

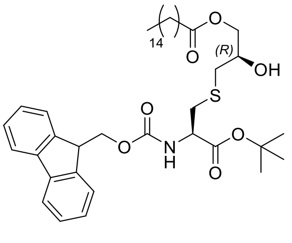

Compound **8** was synthesized according to a reported literature procedure. Spectral data were in good agreement with the literature [3]. To a solution of *tert*-butyl-*N*-(((9*H*-fluoren-9-yl)methoxy)carbonyl)-*S*-((*R*)-2,3-dihydroxypropyl)-L-cysteinate (0.500 g, 0.992 mmol) in anhydrous CH_2Cl_2 (35 mL) under argon at -10 °C was added Et_3N (0.207 mL, 1.49 mmol) and a solution of palmitoyl chloride (0.451 mL, 1.49 mmol) in anhydrous CH_2Cl_2 (15 mL) at -10 °C dropwise *via* syringe. The reaction was stirred for 4 h with the temperature strictly maintained at -10 °C. MeOH (5 mL) was added and the solvents were removed *in vacuo* at 0 °C. The product was purified using silica column

chromatography (n-hexane - EtOAc:n-hexane 3:17) and dried *in vacuo* at 0 °C to yield **8** as a white solid (0.586 g, 83%).

TLC (EtOAc:n-hexane 3:7) R_f = 0.34; ^{1}H NMR (600 MHz, $CDCl_3$) δ 7.78 (d, *J* = 7.5 Hz, 2H, Fmoc-Ar), 7.63 (d, *J* = 7.5 Hz, 2H, Fmoc-Ar), 7.42 (t, *J* = 7.5 Hz, 2H, Fmoc-Ar), 7.34 (t, *J* = 7.5 Hz, 2H, Fmoc-Ar), 5.76 (d, *J* = 7.0 Hz, 1H, NH), 4.58 – 4.52 (m, 1H, Cys-αCH), 4.42 (d, *J* = 6.5 Hz, 2H, Fmoc-CH_2), 4.26 (t, *J* = 6.5 Hz, 1H, Fmoc-CH), 4.18 (dd, *J* = 11.4, 3.7 Hz, 1H, S-glyceryl-OC$\underline{H}_a$$H_b$), 4.13 – 4.07 (m, 1H, S-glyceryl-OC$H_a$$\underline{H}_b$), 3.97 – 3.92 (m, 1H, *S*-glyceryl-CH), 3.11 – 2.95 (m, 2H, βCH_2), 2.87 – 2.79 (m, 1H, S-glyceryl-C$\underline{H}_a$$H_b$), 2.68 – 2.60 (m, 1H, S-glyceryl-C$H_a$$\underline{H}_b$), 2.34 (t, *J* = 7.5 Hz, 2H, Pal-αCH_2), 1.67 – 1.58 (m, 2H, Pal-CH_2), 1.52 (s, 9H, *t*Bu-CH_3), 1.34 – 1.13 (m, 24H, Pal-CH_2), 0.90 (t, *J* = 6.9 Hz, 3H, Pal-CH_3) ppm; ^{13}C NMR (151 MHz, $CDCl_3$) δ 174.0 (Pal C=O), 169.7 (Cys C=O), 156.1 (Fmoc C=O), 144.0 (Fmoc qC), 141.5 (Fmoc qC), 127.9 (Fmoc-Ar-CH), 127.2 (Fmoc-Ar-CH), 125.3 (Fmoc-Ar-CH), 120.2 (Fmoc-Ar-CH), 83.3 (*t*Bu-qC), 69.0 (S-glycery-CH), 67.4 (Fmoc-CH_2), 66.8 (S-glyceryl-OCH_2), 54.6 (Cys-αCH), 47.3 (Fmoc-CH), 37.2 (S-glycery-CH_2), 36.0 (Cys-βCH_2), 34.3 (Pal-αCH_2), 32.1, 29.8, 29.7, 29.6, 29.5, 29.4, 29.3 (Pal-CH_2), 28.2 (*t*Bu-CH_3), 25.0 (Pal- βCH_2), 22.8 (Pal-CH_2), 14.3 (Pal-CH_3) ppm; *m/z* HRMS (ESI$^+$) calcd. $C_{41}H_{61}NNaO_7S$ = 734.4061 $(M + Na)^+$. Found = 734.4063.

(5*R*,9*R*)-5-(*tert*-Butoxycarbonyl)-1-(9*H*-fluoren-9-yl)-3,12-dioxo-2,11-dioxa-7-thia-4-azaheptacosan-9-yl hexadecanoate-*d*$_{31}$ (9)

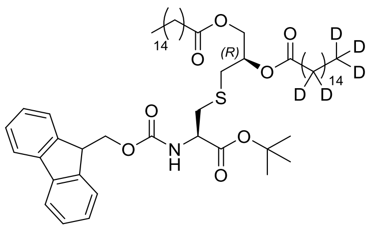

To a solution of palmitic acid-*d*$_{31}$ (0.220 g, 0.764 mmol) in anhydrous CH_2Cl_2 (20 mL) under argon at 0 °C was added EDC·HCl (0.146 g, 0.764 mmol) and DMAP (12 mg, 0.102 mmol). After 1 h of stirring at 0 °C, compound **8** (0.363 g, 0.509 mmol) was added. The reaction was stirred at room temperature for 16 h and the product was purified using silica column chromatography (EtOAc:n-hexane 1:9) to yield **9** as a white solid (0.354 g, 71%).

TLC (EtOAc:n-hexane 1:4) R_f = 0.60; ^{1}H NMR (600 MHz, $CDCl_3$) δ 7.76 (d, *J* = 7.4 Hz, 1H, Fmoc-Ar), 7.62 (d, *J* = 7.4 Hz, 1H, Fmoc-Ar), 7.40 (t, *J* = 7.4 Hz, 1H, Fmoc-Ar), 7.31 (t, *J* = 7.4 Hz, 1H, Fmoc-Ar), 5.71 (d, *J* = 7.5 Hz, 1H, NH), 5.18 – 5.13 (m, 1H, S-glyceryl-CH), 4.54 – 4.48 (m, 1H, Cys-αCH), 4.44 – 4.28 (m, 3H, Fmoc-CH_2, S-glyceryl-OC$\underline{H}_a$$H_b$), 4.24 (t, *J* = 7.1 Hz, 1H, Fmoc-CH), 4.18 – 4.12 (m, 1H, S-glyceryl-OC$H_a$$\underline{H}_b$), 3.09 (dd, *J* = 13.5, 4.5 Hz, 1H, Cys-βC$\underline{H}_a$$H_b$), 3.02 (dd, *J* = 13.5, 4.5 Hz, 1H, Cys-βC$H_a$$\underline{H}_b$), 2.77 (d, *J* = 6.0 Hz, 2H, S-glyceryl-CH_2), 2.29 (t, *J* = 7.7 Hz, 2H, Pal-αCH_2), 1.67 – 1.56 (m, 2H, Pal-βCH_2), 1.49 (s, 9H, *t*Bu-CH_3), 1.33 – 1.16 (m, 24H, Pal-CH_2) , 0.88 (t, *J* = 7.0 Hz, 3H, Pal-CH_3) ppm; ^{13}C NMR (151 MHz, $CDCl_3$) δ 173.3 (Pal C=O), 173.1 (Pal C=O), 169.5 (Cys C=O), 155.7 (Fmoc C=O), 143.8 (Fmoc-qC), 141.3 (Fmoc-qC), 127.7 (Fmoc-Ar-CH), 127.1 (Fmoc-Ar-CH), 125.1 (Fmoc-Ar-CH), 120.0 (Fmoc-Ar-CH), 83.0 (*t*Bu qC), 70.2 (S-glyceryl-CH) , 67.2 (Fmoc-CH_2), 63.5 (S-glyceryl-OCH_2), 54.3 (Cys-αCH), 47.1 (Fmoc-CH), 35.3 (Cys-βCH_2), 34.1 (Pal-αCH_2), 33.3 (S-glyceryl-CH_2), 31.9, 29.7, 29.6, 29.5, 29.3, 29.2, 29.1 (Pal-CH_2), 28.0 (*t*Bu-CH_3), 24.9 (Pal-βCH_2), 22.7 (Pal-CH_2), 14.1 (Pal CH_3) ppm; *m/z* HRMS (ESI$^+$) calcd. $C_{57}H_{60}D_{31}NNaO_8S$ = 1003.8303 $(M + Na)^+$. Found = 1003.8310; ν_{max} (thin film)/cm^{-1}: 2925 (CH_2), 2854 (CH_2), 1737 (CO), 1467 (CH_2), 1369 (CH_3) 1248 (CO), 1155 (CO).

N-(((9H-Fluoren-9-yl)methoxy)carbonyl)-S-((R)-2-((hexadecanoyl-d_{31})oxy)-3-(palmitoyloxy)propyl)-L-cysteine (5)

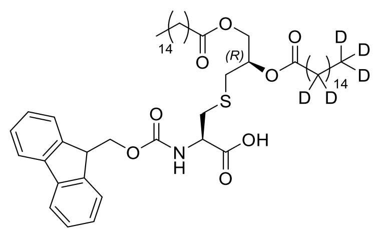

To a solution of compound **9** (0.524 g, 0.535 mmol) in CH_2Cl_2 (3 mL) was added TES (0.5 mL, 3.13 mmol) and TFA (7 mL). The reaction was stirred at room temperature for 4 h. The solvents were removed *in vacuo* and the product recrystallized from a minimum amount of CH_2Cl_2 (5 mL) layered with MeOH (50 mL) for 16 h to yield the product as a white solid (0.450 g, 91%).

TLC (CH_2Cl_2:MeOH 9:1) R_f = 0.53; ^{1}H NMR (600 MHz, $CDCl_3$) δ 7.76 (d, J = 7.3 Hz, 2H, Fmoc-Ar), 7.61 (d, J = 7.3 Hz, 2H, Fmoc-Ar), 7.40 (t, J = 7.3 Hz, 2H, Fmoc-Ar), 7.32 (t, J = 7.3 Hz, 2H, Fmoc-Ar), 5.74 (d, J = 7.3 Hz, 1H, NH), 5.20 – 5.14 (m, 1H, S-glyceryl-CH), 4.68 – 4.62 (m, 1H, Cys-αCH), 4.41 (d, J = 7.0 Hz, 2H, Fmoc-CH_2), 4.35 (dd, J = 12.0, 2.8 Hz, 1H, S-glyceryl-OC$\underline{H}_a$$H_b$), 4.24 (t, J = 7.0 Hz, 1H, Fmoc-CH), 4.15 (dd, J = 12.0, 5.7 Hz, 1H, S-glyceryl-OC$H_a$$\underline{H}_b$), 3.16 (dd, J = 13.8, 4.1 Hz, 1H, Cys-βC$\underline{H}_a$$H_b$), 3.08 (dd, J = 13.8, 4.5 Hz, 1H, Cys-βC$H_a$$\underline{H}_b$), 2.82 – 2.71 (m, 2H, S-glyceryl-CH_2), 2.30 (t, J = 7.6 Hz, 2H, Pal-αCH_2), 1.66 – 1.51 (m, 2H, Pal-βCH_2), 1.39 – 1.07 (m, 24H, Pal-CH_2), 0.88 (t, J = 7.0 Hz, 3H, Pal-CH_3) ppm; ^{13}C NMR (151 MHz, $CDCl_3$) δ 173.7 (Pal C=O), 173.0 (Pal C=O), 156.0 (Cys C=O), 143.8 (Fmoc-qC), 141.5 (Fmoc-qC), 127.9 (Fmoc-Ar-CH), 127.3 (Fmoc-Ar-CH), 125.3 (Fmoc-Ar-CH), 120.2 (Fmoc-Ar-CH), 70.4 (S-glycery-CH), 67.6 (Fmoc-CH_2), 63.7 (S-glyceryl-OCH_2), 53.6 (Cys-αCH), 47.2 (Fmoc-CH), 34.7 (Cys-βCH_2), 34.3 (Pal-CH_2), 33.1 (S-glycery-CH_2), 32.1, 29.9, 29.8, 29.7, 29.6, 29.5, 29.4, 29.3, 25.0, 22.9 (Pal-CH_2), 14.3 (Pal-CH_3) ppm; *m/z* HRMS (ESI$^+$) calcd. $C_{53}H_{52}D_{31}NO_8SNa$ = 947.7677 (M + Na)$^+$. Found = 947.7669. ν_{max} (thin film)/cm^{-1}: 1741 (CO), 1498 (CH_2), 1352 (CH_3) 1270 (CO).

Peptide Synthesis

Peptides **1**-**3** were prepared *via* the synthesis of the resin bound common intermediate **10** which was then functionalized with either Fmoc-Cys((*R*)-2,3-bis(palmitoyloxy)propyl)-OH, Fmoc-Cys((*S*)-2,3-bis(palmitoyloxy)propyl)-OH (**4**) or Fmoc-Cys((*R*)-2-((hexadecanoyl-d_{31})oxy)-3-(palmitoyloxy)propyl)-OH (**5**) as indicated in Supplementary Fig. 39.

Common resin intermediate (10)

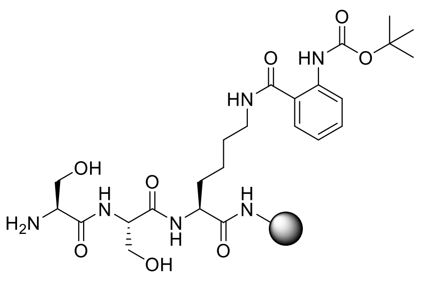

To a polypropylene syringe reaction vessel was added rink amide (aminomethyl)polystyrene resin (209 mg, 0.150 mmol; initial loading 0.70 mmol/g) and DMF (5 mL). The syringe was agitated for 20 min and then drained. A solution of 20 %(v/v) piperidine in DMF (5 mL) was added for 2 x 10 min. The Fmoc deprotected resin was drained and washed with DMF (3 x 5 mL), CH_2Cl_2 (3 x 5 mL) and

DMF (3 x 5 mL). A solution of Fmoc-Lys(N^{ε}-4-methyltrityl)-OH (4 equiv, 365 mg, 0.580 mmol), PyBOP (4 equiv, 304 mg, 0.580 mmol), NMM (8 equiv, 0.129 mL, 1.17 mmol) in DMF (3 mL) was added to the resin and agitated for 45 min. The reaction vessel was drained and the resin was washed with DMF (3 x 5 mL), CH_2Cl_2 (3 x 5 mL), DMF (3 x 5 mL) and CH_2Cl_2 (3 x 5 mL). A solution of 5 %(v/v) TFA, 5 %(v/v) TES, 90 %(v/v) CH_2Cl_2 (5 mL) was added to the resin for 5 x 1 min to remove 4-methyltrityl. The resin was drained and washed with CH_2Cl_2 (3 x 5 mL), DMF (3 x 5 mL), CH_2Cl_2 (3 x 5 mL) and DMF (3 x 5 mL). A solution of Boc-Abz-OH (3 equiv, 104 mg, 0.438 mmol), PyBOP (3 equiv, 228 mg, 0.438 mmol) and NMM (6 equiv, 0.096 mL, 0.876 mmol) in DMF (2 mL) was added to the syringe which was agitated for 45 min then drained and washed with DMF (3 x 5 mL), CH_2Cl_2 (3 x 5 mL) then DMF (3 x 5 mL). Two sequential couplings of Fmoc-Ser(*t*Bu)-OH were then performed on the resin using coupling cycles consisting of (i) Fmoc deprotection using 20 %(v/v) piperidine in DMF (2 x 10 min; 5 mL), (ii) resin washing with DMF (3 x 5 mL), CH_2Cl_2 (3 x 5 mL) then DMF (3 x 5 mL), (iii) peptide coupling by the addition of PyBOP (3 equiv, 228 mg, 0.438 mmol), NMM (6 equiv, 0.096 mL, 0.876 mmol) and Fmoc-Ser(*t*Bu)-OH (3 equiv, 168 mg, 0.438 mmol) in DMF (2 mL) to the peptide resin for 45 min, (iv) resin washing with DMF (3 x 5 mL), CH_2Cl_2 (3 x 5 mL) then DMF (3 x 5 mL). Following the second coupling cycle of Fmoc-Ser(*t*Bu)-OH the resin was treated with 20 %(v/v) piperidine in DMF (2 x 10 min; 5 mL). The resin was drained and washed with DMF (3 x 5 mL), CH_2Cl_2 (3 x 5 mL) and DMF (3 x 5 mL) and used directly in the next step.

(7*S*,10*S*,13*S*,16*R*,20*R*)-16-Amino-1-(2-aminophenyl)-7-carbamoyl-10,13-bis(hydroxymethyl)-1,9,12,15-tetraoxo-18-thia-2,8,11,14-tetraazahenicosane-20,21-diyl dipalmitate (1)

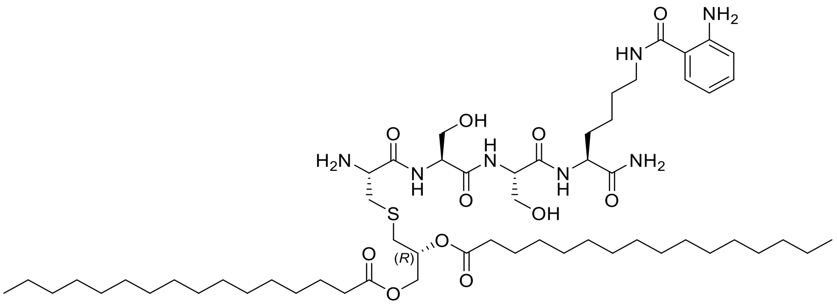

To peptide resin **10** (0.146 mmol) was added a solution of Fmoc-Cys((*R*)-2,3-bis(palmitoyloxy)propyl)-OH (3 equiv, 392 mg, 0.438 mmol), PyBOP (3 equiv, 228 mg, 0.438 mmol) and NMM (6 equiv, 0.096 mL, 0.876 mmol) in DMF (2 mL). The reaction was agitated for 45 min at room temperature then drained and washed with DMF (3 x 5 mL), CH_2Cl_2 (3 x 5 mL) and DMF (3 x 5 mL). A solution of 20 %(v/v) piperidine in DMF (5 mL) was added to the resin for 2 x 10 min. The resin was washed with DMF (3 x 5 mL), CH_2Cl_2 (3 x 5 mL), DMF (3 x 5 mL) and CH_2Cl_2 (3 x 5 mL). The resin was then dried under reduced pressure. The dry resin was swollen in CH_2Cl_2 (5 mL) under agitation for 20 min, then drained. The cleavage cocktail consisting of 95 %(v/v) TFA, 2.5 %(v/v) TES and 2.5 %(v/v) H_2O (5 mL) was added to the syringe and agitated for 90 min. The cleavage cocktail was drained and collected. The resin was washed with cleavage cocktail (2 x 2.5 mL) and the combined solution was concentrated under a stream of N_2 followed by precipitation of the peptide with Et_2O (10 mL) at 0 °C. The crude peptide was collected by centrifugation and washed with Et_2O (2 x 10 mL) at 0 °C. The crude material was dried *in vacuo* at 0 °C and the peptide was purified by silica column chromatography (CH_2Cl_2 – CH_2Cl_2:MeOH 9:1), and dried *in vacuo* at 0 °C to yield **1** as a white solid (14.0 mg, 9%).

TLC (CH_2Cl_2:MeOH 9:1) R_f = 0.31; ^{1}H NMR (600 MHz, DMSO-d_6) δ 8.21 – 8.13 (m, 3H, Lys-εNH, Cys-Ser-NH, Lys-Ser-NH), 7.87 (d, *J* = 7.9 Hz, 1H, Lys-NH), 7.45 (d, *J* = 8.0, Hz, 1H, Abz-Ar-CH), 7.16 – 7.07 (m, 3H, $CONH_2$, Abz-Ar-CH), 6.68 (d, *J* = 8.0 Hz, 1H, Abz-Ar-CH), 6.53 – 6.47 (m, 1H, Abz-Ar-CH), 6.35

(s, 2H, Abz-NH_2), 5.24 – 5.19 (m, 1H, Cys-Ser-OH), 5.14 – 5.08 (m, 1H, S-glyceryl-CH), 5.08 – 5.04 (m, 1H, Lys-Ser-OH), 4.41 - 4.36 (m, 1H, Cys-Ser-αCH), 4.34 – 4.26 (m, 2H, Lys-Ser-αCH, S-glyceryl-OC$H_a$$H_b$), 4.15 – 4.07 (m, 2H, Lys-αCH, S-glyceryl-OC$H_a$$H_b$), 3.72 – 3.64 (m, 2H, Cys-Ser-C$H_a$$H_b$, Lys-Ser-C$H_a$$H_b$), 3.61 – 3.55 (m, 1H, Lys-Ser-C$H_a$$H_b$), 3.55 – 3.49 (m, 1H, Cys-Ser-C$H_a$$H_b$), 3.41 – 3.38 (m, 1H, Cys-αCH), 3.17 (dd, *J* = 12.0, 5.6 Hz, 2H, Lys-εCH_2), 2.88 (dd, *J* = 13.3, 4.3 Hz, 1H, Cys-C$H_a$$H_b$), 2.80 (dd, *J* = 14.1, 5.7 Hz, 1H, S-glyceryl-C$H_a$$H_b$), 2.70 (dd, *J* = 14.1, 7.3 Hz, 1H, S-glyceryl-C$H_a$$H_b$), 2.61 – 2.57 (m, 1H, Cys-C$H_a$$H_b$), 2.31 – 2.24 (m, 4H, Pal-αCH_2 x 2), 1.78 – 1.71 (m, 1H, Lys-βC$H_a$$H_b$), 1.57 – 1.42 (m, 7H, Lys-βC$H_a$$H_b$, Pal-$CH_2$ x 2, Lys-*δ*CH_2), 1.38 – 1.32 (m, 2H, Lys-γCH_2), 1.31 – 1.17 (m, 48H, Pal-CH_2 x 24), 0.86 (t, *J* = 6.9 Hz, 6H, Pal-CH_3 x 2) ppm; ^{13}C NMR (151 MHz, DMSO-d_6) δ 173.8 ($CONH_2$), 172.5 (Pal C=O), 172.3 (Pal C=O), 170.5 (Cys-Ser C=O), 169.7 (Lys-Ser C=O), 168.8 (Abz C=O), 149.5 (Abz qC-NH_2), 131.4 (Abz-Ar-CH), 128.0 (Abz-Ar-CH), 116.3 (Abz-Ar-CH), 115.0 (Abz qC), 114.5 (Abz Ar-CH), 70.0 (S-glyceryl-CH), 63.5 (S-glyceryl-OCH_2), 62.0 (Cys-Ser-βCH_2), 61.4 (Lys-Ser-βCH_2), 55.5 (Lys-Ser-αCH), 54.4 (Cys-Ser-αCH), 54.2 (Cys-αCH), 52.8 (Lys-αCH), 38.7 (Lys-εCH_2), 37.5 (Cys-βCH_2), 33.6 (Pal-αCH_2), 33.4 (Pal-αCH_2), 31.7 (Lys-βCH_2), 31.3 (S-glyceryl-CH_2), 29.1 , 29.0, 28.9, 28.8, 28.7, 28.6, 28.4, 28.4 (Pal-CH_2), 24.5 , 24.4 (Pal-βCH_2), 23.1 (Lys-γCH_2), 22.1 (Pal-CH_2), 13.9 (Pal-CH_3) ppm; *m/z* HRMS (ESI^+) calcd. $C_{57}H_{102}N_7O_{11}S$ = 1092.7353 $(M + H)^+$. Found = 1092.7350; ν_{max} (thin film)/cm^{-1}: 3287 (Amide A: NH), 2919 (CH_2), 2851 (CH_2), 1674 (Amide I: CO, CN), 1534 (Amide II: CN, NH), 1440, 1303 (Amide III: CN, CO), 1203 (CO).

(7*S*,10*S*,13*S*,16*R*,20*S*)-16-Amino-1-(2-aminophenyl)-7-carbamoyl-10,13-bis(hydroxymethyl)-1,9,12,15-tetraoxo-18-thia-2,8,11,14-tetraazahenicosane-20,21-diyl dipalmitate (2)

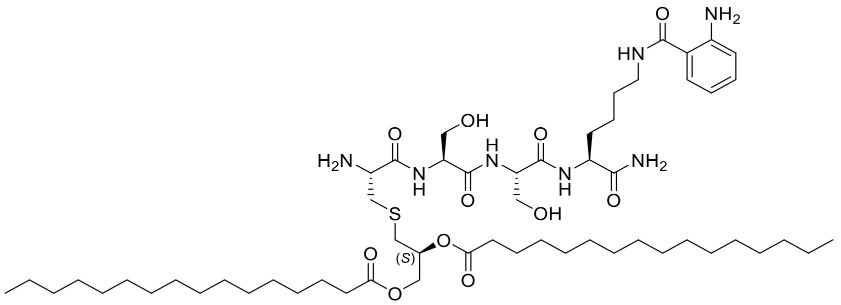

To peptide resin **10** (0.073 mmol) was added a solution of Fmoc-Cys((*S*)-2,3-bis(palmitoyloxy)propyl)-OH (**4**) (3 equiv, 195 mg, 0.219 mmol), PyBOP (3 equiv, 114 mg, 0.219 mmol) and NMM (6 equiv, 0.048 mL, 0.438 mmol) in DMF (2 mL). The reaction was agitated for 45 min at room temperature and then drained. The resin was washed with DMF (3 x 5 mL), CH_2Cl_2 (3 x 5 mL) and DMF (3 x 5 mL). A solution of 20 %(v/v) piperidine in DMF (5 mL) was added to the resin for 2 x 10 min. The resin was washed with DMF (3 x 5 mL), CH_2Cl_2 (3 x 5 mL), DMF (3 x 5 mL) and CH_2Cl_2 (3 x 5 mL). The resin was then dried under reduced pressure. The dry resin was swollen in CH_2Cl_2 (5 mL) under agitation for 20 min, then drained. The cleavage cocktail consisting of 95 %(v/v) TFA, 2.5 %(v/v) TES and 2.5 %(v/v) H_2O (5 mL) was added to the syringe which was agitated for 90 min. The cleavage cocktail was drained and collected. The resin was washed with cleavage cocktail (2 x 2.5 mL) and the combined solution was concentrated under a stream of N_2 followed by precipitation of the peptide with Et_2O (10 mL) at 0 °C. The crude peptide was collected by centrifugation and washed with Et_2O (2 x 10 mL) at 0 °C. The crude material was dried *in vacuo* at 0 °C and the peptide was purified by silica column chromatography (CH_2Cl_2 – CH_2Cl_2:MeOH 9:1), and dried *in vacuo* at 0 °C to yield **2** as a white solid (5.0 mg, 6%).

TLC (CH_2Cl_2:MeOH 9:1) R_f = 0.31; ^{1}H NMR (600 MHz, DMSO-d_6) δ 8.27 – 8.13 (m, 3H, Lys-εNH, Cys-Ser-NH, Lys-Ser-NH), 7.86 (d, *J* = 7.7 Hz, 1H, Lys-NH), 7.44 (dd, *J* = 8.2, 1.0 Hz, 1H, Abz-Ar-CH), 7.19 – 7.00 (m, 1H, $CONH_2$, Abz-Ar-CH), 6.66 (dd, *J* = 8.2, 1.0 Hz, 1H, Abz-Ar-CH), 6.56 – 6.44 (m, 1H, Abz-Ar-

CH), 6.34 (s, 2H, Abz-NH_2), 5.21 (t, *J* = 5.3 Hz, 1H, Cys-Ser-OH), 5.15 – 5.08 (m, 1H, S-glyceryl-CH), 5.05 (t, *J* = 5.5 Hz, 1H, Lys-Ser-OH), 4.44 – 4.34 (m, 1H, Cys-Ser-αCH), 4.35 – 4.22 (m, 2H, Lys-Ser-αCH, S-glyceryl-OCH_aH_b), 4.16 – 4.03 (m, 2H, Lys-αCH, S-glyceryl-OCH_aH_b), 3.73 – 3.61 (m, 2H, Cys-Ser-CH_aH_b, Lys-Ser-CH_aH_b), 3.60 – 3.55 (m, 1H, Lys-Ser-CH_aH_b), 3.54 – 3.47 (m, 1H, Cys-Ser-CH_aH_b), 3.45 – 3.39 (m, 1H, Cys-αCH), 3.16 (dd, *J* = 12.8, 7.0 Hz, 2H, Lys-εCH_2), 2.88 (dd, *J* = 13.2, 4.4 Hz, 1H, Cys-CH_aH_b), 2.80 (dd, *J* = 13.9, 5.5 Hz, 1H, S-glyceryl-CH_aH_b), 2.73 – 2.65 (m, 1H, S-glyceryl-CH_aH_b), 2.65 – 2.57 (m, 1H, Cys-CH_aH_b), 2.29 – 2.23 (m, 4H, Pal-αCH_2 x 2), 1.77 – 1.69 (m, 2H, Lys-βCH_aH_b), 1.58 – 1.44 (m, 7H, Lys-βCH_aH_b,Pal-CH_2 x 2, Lys-*δ*CH_2), 1.36 – 1.31 (m, 2H, Lys-γCH_2), 1.31 – 1.17 (m, 48H, Pal-CH_2 x 24), 0.85 (t, *J* = 6.9 Hz, 6H, Pal-CH_3 x 2); ^{13}C NMR (151 MHz, DMSO-d_6) δ 173.7 ($CONH_2$), 172.5 (Pal C=O), 172.3 (Pal C=O), 170.4 (Cys-Ser C=O), 169.7 (Lys-Ser C=O), 168.7 (Abz C=O), 149.5 (Abz qC-NH_2), 131.4 (Abz-Ar-CH), 128.0 (Abz-Ar-CH), 116.2 (Abz-Ar-CH), 115.0 (Abz qC), 114.5 (Abz Ar-CH), 70.0 (S-glyceryl-CH), 63.5 (S-glyceryl-OCH_2), 61.9 (Cys-Ser-βCH_2), 61.4 (Lys-Ser-βCH_2), 55.5 (Lys-Ser-αCH), 54.4 (Cys-Ser-αCH), 54.0 (Cys-αCH), 52.7 (Lys-αCH), 38.7 (Lys-εCH_2), 37.2 Cys-βCH_2), 33.6, 33.4, 31.6, 31.3, 31.2, 29.1, 29.0, 28.9, 28.9, 28.8, 28.7, 28.4 (Pal-αCH_2), 24.5, 24.4(Pal-βCH_2), 23.1 (Lys-γCH_2), 22.1 (Pal-CH_2), 13.9 (Pal-CH_3) ppm; *m/z* HRMS (ESI$^+$) calcd. $C_{57}H_{102}N_7O_{11}S$ = 1092.7353 (M + H)$^+$. Found = 1092.7345; ν_{max} (thin film)/cm^{-1}: 3354 (Amide A: NH), 2920 (CH_2), 2855 (CH_2), 1636 (Amide I: CO, CN), 1560 (Amide II: CN, NH), 1223 (Amide III: CN, CO), 1040 (CO).

(7*S*,10*S*,13*S*,16*R*,20*R*)-16-Amino-1-(2-aminophenyl)-7-carbamoyl-10,13-bis(hydroxymethyl)-1,9,12,15,23-pentaoxo-22-oxa-18-thia-2,8,11,14-tetraazaoctatriacontan-20-yl hexadecanoate-*d*$_{31}$ (3)

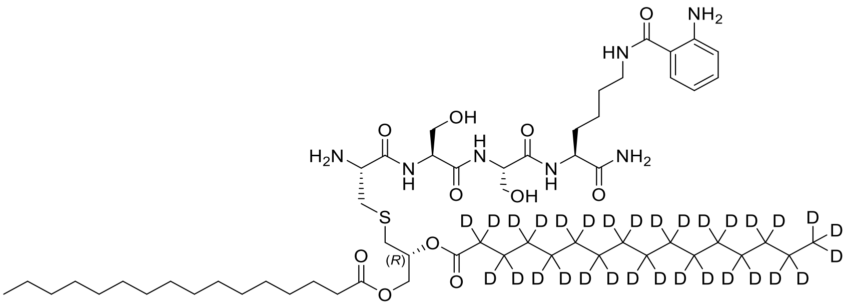

To peptide resin **10** (0.178 mmol) was added a solution of Fmoc-Cys((*R*)-2-((hexadecanoyl-*d*$_{31}$)oxy)-3-(palmitoyloxy)propyl)-OH (**5**) (3 equiv, 494 mg, 0.534 mmol), PyBOP (3 equiv, 278 mg, 0.534 mmol) and NMM (6 equiv, 0.117 mL, 1.068 mmol) in DMF (2 mL). The reaction was agitated for 45 min at room temperature, drained and was washed with DMF (3 x 5 mL), CH_2Cl_2 (3 x 5 mL) and DMF (3 x 5 mL). A solution of 20 %(v/v) piperidine in DMF (5 mL) was added to the resin for 2 x 10 min. The resin was drained and washed with DMF (3 x 5 mL), CH_2Cl_2 (3 x 5 mL), DMF (3 x 5 mL) and CH_2Cl_2 (3 x 5 mL). The resin was then dried under reduced pressure swollen in CH_2Cl_2 (5 mL) under agitation for 20 min, then drained. The cleavage cocktail consisting of 95 %(v/v) TFA, 2.5 %(v/v) TES and 2.5 %(v/v) H_2O (5 mL) was added to the syringe which was agitated for 90 min. The cleavage cocktail was drained and collected. The resin was washed with cleavage cocktail (2 x 2.5 mL) and the combined solution was concentrated under a stream of N_2 followed by precipitation of the peptide with Et_2O (10 mL) at 0 °C. The crude peptide was collected by centrifugation and washed with Et_2O (2 x 10 mL) at 0 °C. The crude material was dried *in vacuo* at 0 °C and the peptide was purified by silica column chromatography (CH_2Cl_2 – CH_2Cl_2:MeOH 9:1), and dried *in vacuo* at 0 °C to yield **3** as a white solid (18.0 mg, 9%).

TLC (CH_2Cl_2:MeOH 9:1) R_f = 0.31; ^{1}H NMR (600 MHz, DMSO-d_6) δ 8.23 – 8.11 (m, 3H, Lys-εNH, Cys-Ser-NH, Lys-Ser-NH), 7.85 (d, *J* = 7.8 Hz, 1H, Lys-NH), 7.44 (d, *J* = 8.0 Hz, 1H, Abz-Ar-CH), 7.15 – 7.04 (m, 3H, $CONH_2$, Abz-Ar-CH), 6.66 (d, *J* = 8.0, Hz, 1H, Abz-Ar-CH), 6.56 – 6.43 (m, 1H, Abz-Ar-CH), 6.34

(s, 2H, Abz-NH$_2$), 5.20 (t, *J* = 5.6 Hz, 1H, Cys-Ser-OH), 5.12 – 5.07 (m, 1H, S-glyceryl-CH), 5.05 (t, *J* = 5.6 Hz, 1H, Lys-Ser-OH), 4.42 – 4.34 (m, 1H, Cys-Ser-αCH), 4.34 – 4.24 (m, 2H, Lys-Ser-αCH, S-glyceryl-OCH$_a$H$_b$), 4.20 – 4.03 (m, 2H, Lys-αCH, S-glyceryl-OCH$_a$H$_b$), 3.75 – 3.61 (m, 2H, Cys-Ser-CH$_a$H$_b$, Lys-Ser-CH$_a$H$_b$), 3.61 – 3.55 (m, 1H, Lys-Ser-CH$_a$H$_b$), 3.55 – 3.47 (m, 1H, Cys-Ser-CH$_a$H$_b$), 3.41 – 3.37 (m, 1H, Cys-αCH), 3.16 (dd, *J* = 13.0, 7.1 Hz, 1H, Lys-εCH$_2$), 2.87 (dd, *J* = 13.0, 4.2 Hz, 1H, Cys-CH$_a$H$_b$), 2.79 (dd, *J* = 14.0, 5.9 Hz, 1H, S-glyceryl-CH$_a$H$_b$), 2.69 (dd, *J* = 14.0, 7.3 Hz, 1H, S-glyceryl-CH$_a$H$_b$), 2.59 (dd, *J* = 13.0, 8.1 Hz, 1H, Cys-CH$_a$H$_b$), 2.26 (t, *J* = 7.3 Hz, 2H, Pal-αCH$_2$), 1.79 – 1.69 (m, 1H, Lys-βCH$_a$H$_b$), 1.56 – 1.43 (m, 5H, Lys-βCH$_a$H$_b$, Pal-CH$_2$, Lys-*δ*CH$_2$), 1.37 – 1.30 (m, 2H, Lys-γCH$_2$), 1.29 – 1.17 (m, 24H, Pal-CH$_2$ x 12)), 0.85 (t, *J* = 6.9 Hz, 3H, Pal-CH$_3$) ppm; ^{13}C NMR (151 MHz, DMSO-*d*$_6$) δ 173.7 (CONH$_2$), 172.5 (Pal C=O), 172.3 (Pal C=O), 170.5 (Cys-Ser C=O), 169.7 (Lys-Ser C=O), 168.7 (Abz C=O), 149.5 (Abz qC-NH$_2$), 131.4 (Abz-Ar-CH), 128.0 (Abz-Ar-CH), 116.2 (Abz-Ar-CH), 115.0 (Abz qC), 114.5 (Abz Ar-CH), 70.0 (S-glyceryl-CH), 63.5 (S-glyceryl-OCH$_2$), 61.9 (Cys-Ser-βCH$_2$), 61.4 (Lys-Ser-βCH$_2$), 55.5 (Lys-Ser-αCH), 54.5 (Cys-Ser-αCH), 54.2 (Cys-αCH), 52.8 (Lys-αCH), 38.7 (Lys-εCH$_2$), 37.4 (Cys-βCH$_2$), 33.4 (Pal-αCH$_2$), 31.6 (Lys-βCH$_2$), 31.3 (S-glyceryl-CH$_2$), 29.1, 28.9, 28.9, 28.9, 28.7, 28.7, 28.4 (Pal-CH$_2$), 24.4 (Pal-βCH$_2$), 23.1 (Lys-γCH$_2$), 22.1 (Pal-CH$_2$), 13.9 (Pal-CH$_3$) ppm; *m/z* HRMS (ESI$^+$) calcd. $C_{57}H_{71}D_{31}N_7O_{11}S$ = 1123.9226 (M + H)$^+$. Found = 1123.9332; ν$_{max}$ (thin film)/cm^{-1}: 3287 (Amide A: NH), 2920 (CH$_2$), 2851 (CH$_2$), 1676 (Amide I: CO, CN), 1545 (Amide II: CN, NH), 1261 (Amide III: CN, CO), 1021 (CO).

Lit Reaction Product Characterization

Lyso-FP2 (15)

(7*S*,10*S*,13*S*,16*R*,20*R*)-1-(2-Aminophenyl)-7-carbamoyl-20-hydroxy-10,13-bis(hydroxymethyl)-1,9,12,15-tetraoxo-16-palmitamido-18-thia-2,8,11,14-tetraazahenicosan-21-yl palmitate (**15**)

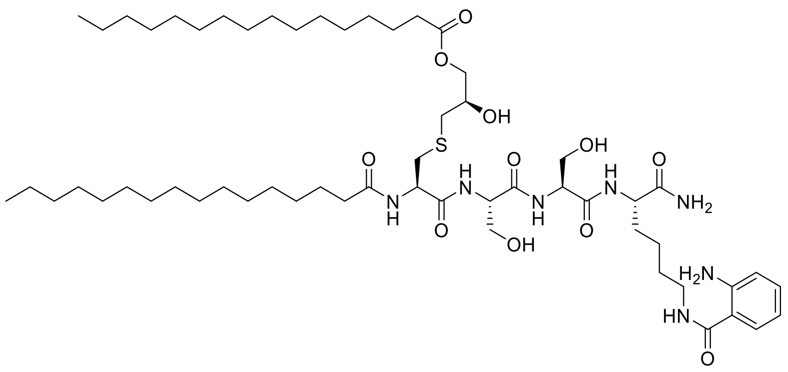

Lyso-dFP2 (**15**) was prepared as described in Materials and Methods.

TLC (CHCl$_3$:MeOH:NH$_4$OH 8:2:0.1) *R*$_f$ = 0.71; ^{1}H NMR (600 MHz, DMSO-*d*$_6$) δ 8.18 – 8.13 (m, 1H, Lys-εNH), 8.14 – 8.08 (m, 1H, Ser-NH), 8.08 – 8.01 (m, 2H, Cys-NH, Ser-NH), 7.86 (d, *J* = 8.1 Hz, 1H, Lys-NH), 7.44 (d, *J* = 7.7 Hz, 1H, Abz-Ar-CH), 7.14 – 7.07 (m, 3H, CONH$_2$, Abz-Ar-CH), 6.66 (d, *J* = 8.1 Hz, 1H, Abz-Ar-CH), 6.52 – 6.46 (m, 1H, Abz-Ar-CH), 6.34 (s, 2H, Abz-NH$_2$), 5.21 – 5.11 (m, 2H, Ser-OH, S-glyceryl-CH-OH), 5.11 – 5.01 (m, 1H, Ser-OH), 4.52 – 4.43 (m, 1H, Cys-αCH), 4.41 – 4.31 (m, 1H, Ser-αCH), 4.31 – 4.21 (m, 1H, Ser-αCH), 4.17 – 4.07 (m, 1H, Lys-αCH), 4.02 – 3.91 (m, 2H, S-glyceryl-OCH$_2$), 3.83 – 3.75 (m, 1H, S-glyceryl-CH), 3.71 – 3.59 (m, 2H, Ser-CH$_a$H$_b$, Ser-CH$_a$H$_b$), 3.59 – 3.45 (m, 2H, Ser-CH$_a$H$_b$, Ser-CH$_a$H$_b$), 3.19 – 3.13 (m, 2H, Lys-εCH$_2$), 2.93 – 2.85 (m, 1H, Cys-CH$_a$H$_b$), 2.68 – 2.55 (m, 3H, Cys-CH$_a$H$_b$, S-glyceryl-CH$_2$), 2.34 – 2.23 (m, 2H, O-Pal-αCH$_2$), 2.17 – 2.06 (m, 2H, NH-Pal-αCH$_2$), 1.80 – 1.69 (m, 3H, Lys-βCH$_a$H$_b$), 1.62 – 1.39 (m, 7H, Lys-βCH$_a$H$_b$, Pal-CH$_2$ x 2, Lys-*δ*CH$_2$), 1.39 – 1.30 (m, 2H, Lys-γCH$_2$), 1.30 – 1.10 (m, 48H, Pal-CH$_2$ x 24), 0.89 – 0.79 (m, 6H, Pal-CH$_3$ x 2) ppm; *m/z* HRMS (ESI$^+$) calcd. $C_{57}H_{102}N_7O_{11}S$ = 1092.7353 (M + H)$^+$. Found = 1092.7365

Lyso-dFP2 (16)

(7*S*,10*S*,13*S*,16*R*,20*R*)-1-(2-Aminophenyl)-7-carbamoyl-16-(hexadecanamido-2,2,3,3,4,4,5,5,6,6,7,7,8,8,9,9,10,10,11,11,12,12,13,13,14,14,15,15,16,16,16-d_{31})-20-hydroxy-10,13-bis(hydroxymethyl)-1,9,12,15-tetraoxo-18-thia-2,8,11,14-tetraazahenicosan-21-yl palmitate (**16**)

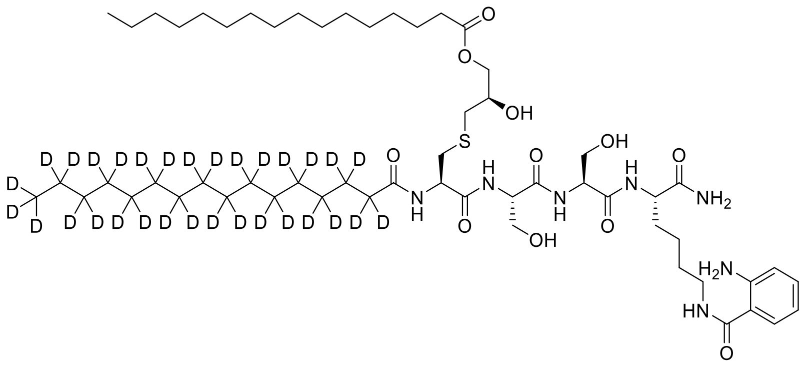

Lyso-dFP2 (**16**) was prepared as described in Materials and Methods.

TLC ($CHCl_3$:MeOH:NH_4OH 8:2:0.1) R_f = 0.71; ^1H NMR (600 MHz, DMSO-d_6) δ 8.18 – 8.14 (m, 1H, Lys-εNH), 8.11 – 8.08 (m, 1H, Ser-NH), 8.07 – 8.01 (m, 2H, Cys-NH, Ser-NH), 7.85 (d, *J* = 7.8 Hz, 1H, Lys-NH), 7.44 (d, *J* = 7.3 Hz, 1H, Abz-Ar-CH), 7.13 – 7.09 (m, 3H, $CONH_2$, Abz-Ar-CH), 6.66 (d, *J* = 8.1 Hz, 1H, Abz-Ar-CH), 6.51 – 6.47 (m, 1H, Abz-Ar-CH), 6.34 (s, 2H, Abz-NH_2), 5.19 – 5.13 (m, 2H, Ser-OH, S-glyceryl-CH-O<u>H</u>), 5.07 – 5.03 (m, 1H, Ser-OH), 4.50 – 4.45 (m, 1H, Cys-αCH), 4.36 – 4.31 (m, 1H, Ser-αCH), 4.29 – 4.24 (m, 1H, m, 1H, Ser-αCH), 4.14 – 4.08 (m, 1H, Lys-αCH), 4.05 – 3.93 (m, 2H, S-glyceryl-OCH_2), 3.83 – 3.77 (m, 1H, S-glyceryl-CH), 3.71 – 3.61 (m, 2H, Ser-C$\underline{H_a}H_b$, Ser-C$\underline{H_a}H_b$), 3.59 – 3.52 (m, 2H, Ser-$CH_a\underline{H_b}$, Ser-$CH_a\underline{H_b}$), 3.19 – 3.14 (m, 2H, Lys-εCH_2), 2.92 – 2.86 (m, 1H, Cys-C$\underline{H_a}H_b$), 2.68 – 2.62 (m, 3H, Cys-$CH_a\underline{H_b}$, S-glyceryl-CH_2), 2.31 – 2.25 (m, 2H, O-Pal-αCH_2), 1.77 – 1.70 (m, 3H, Lys-βC$\underline{H_a}H_b$), 1.54 – 1.43 (m, 7H, Lys-β$CH_a\underline{H_b}$, Pal-CH_2, Lys-δCH_2), 1.30 – 1.19 (m, 24H, Pal-CH_2 x 12), 0.85 (t, *J* = 6.9 Hz, 3H, Pal-CH_3) ppm; *m/z* HRMS (ESI$^+$) calcd. $C_{57}H_{71}D_{31}N_7O_{11}S$ = 1123.9226 $(M + H)^+$. Found = 1123.9385.

Supplementary Figs. 1-53

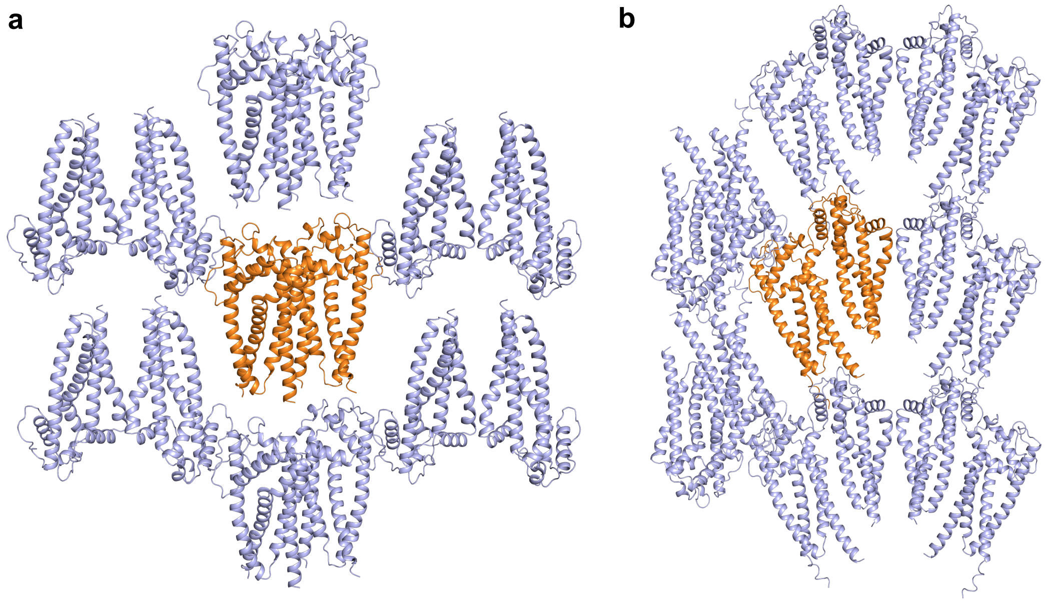

Supplementary Figure 1. Packing arrangement in Lit crystals. (**a**) Structure at 2.27 Å resolution in space group $P2_1$. (**b**) Structure at 1.95 Å resolution in space group $P2_12_12$. The packing in **a** is classically type 1 or layered as expected for *in meso* grown crystals. In **b**, layered packing is less obvious and may have emerged as a result of a polymorphic transition from an initial type 1 packed crystal[4]. The precipitant used in **a** included 100 mM sodium citrate pH 5.5, 40 %(v/v) PEG400, and 100 mM ammonium sulfate which induced formation of the sponge phase. The precipitant used in **b** included 100 mM sodium citrate pH 5.5, 30-38 %(v/v) PEG200, and 50-100 mM sodium chloride which stabilized the cubic or the sponge phase depending on the PEG200 and sodium chloride concentrations. Bigger crystals tended to grow in the sponge phase. However, no difference was observed in the diffraction limits of crystals from either mesophase.

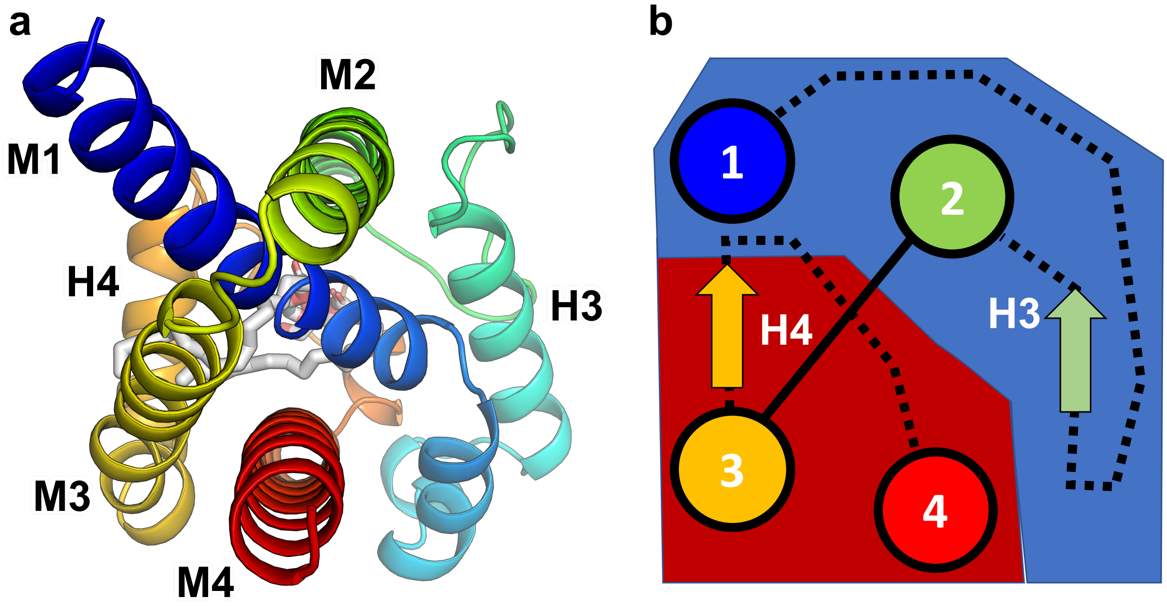

Supplementary Figure 2. Lit viewed from the cytoplasm to reveal structural pseudosymmetry. (**a**) Transmembrane helices M2 and M3 spay apart as they cross the membrane. The loop where they come together in the cytoplasm is straddled pseudosymmetrically by M1 and M4. The symmetry extends to helices H3 and H4 in EGD1 and EGD2, respectively. (**b**) Simplified representation of the structure in **a** to highlight elements of symmetry. The N-terminal and C-terminal halves of the protein have blue and red backgrounds, respectively. Membrane and extracytoplasmic helices are shown as discs and arrows, respectively.

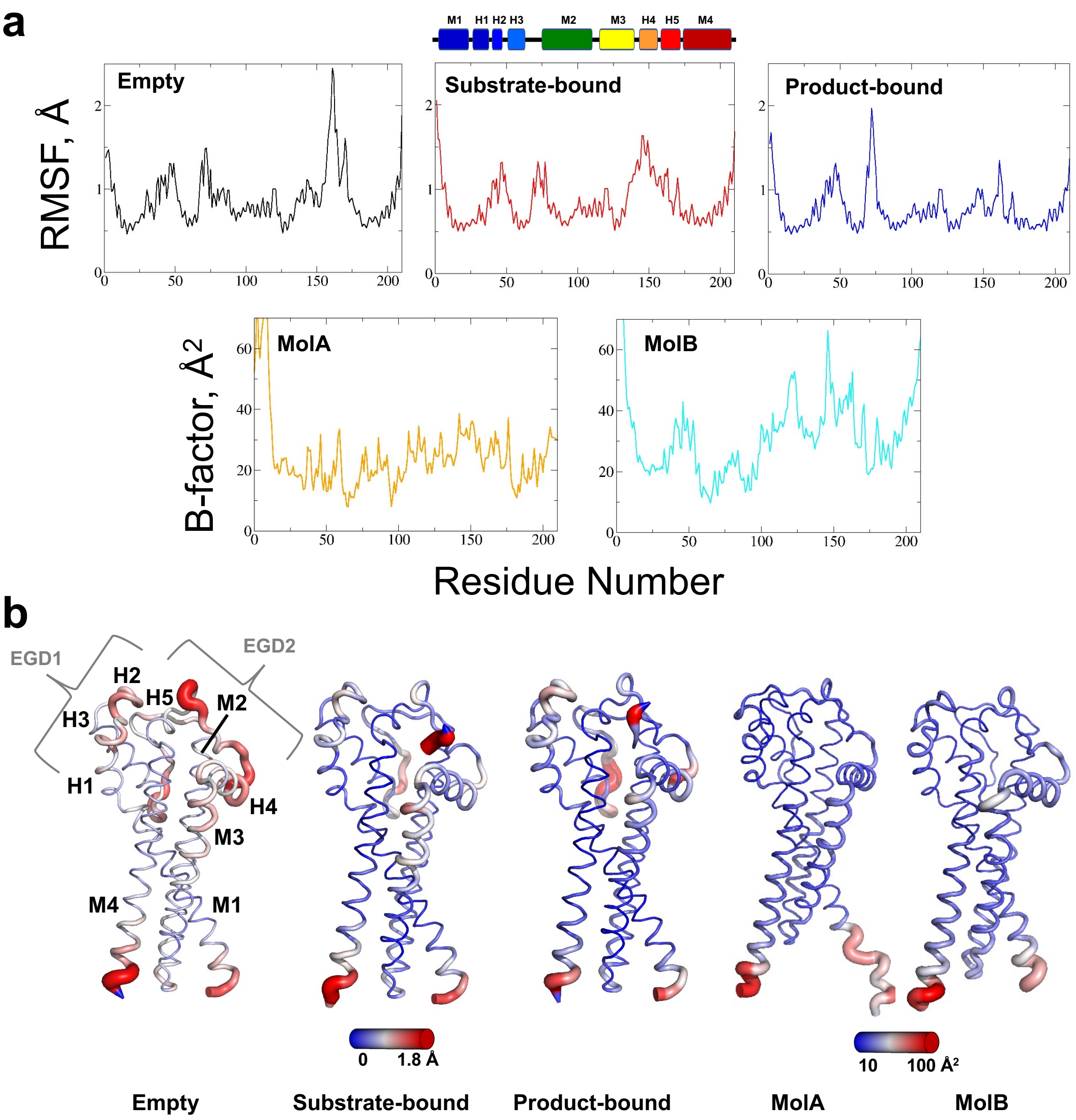

Supplementary Figure 3. Stability and flexibility revealed in different parts of Lit by MD simulations of the enzyme in a lipid membrane and by crystallographic B-factor. Simulations were run with three variants of the crystal structure model. These included i) an empty model where the internal monoolein molecules in the crystal structure were removed, ii) a model where a lipopeptide substrate was positioned in the active site pocket, and iii) a model where a lyso-lipopeptide product was placed in the active site pocket. (**a**) The average root-mean-square fluctuation (RMSF) of the C_α atoms in Lit derived from five 500 ns MD simulation replica runs reveals enhanced flexibility in EGD1 (H1, H2, H3), EGD2 (H4, H5) and the extracytoplasmic half of M3. B-factors are shown for molecules A and B. (**b**) The average RMSF of C_α atoms mapped onto the average structure of Lit in putty representation from simulations of empty, substrate- and product-bound forms. Likewise, B-factors are shown for molecules A and B. The structure colour and cartoon thickness are scaled to the average RMSF and B-factor.

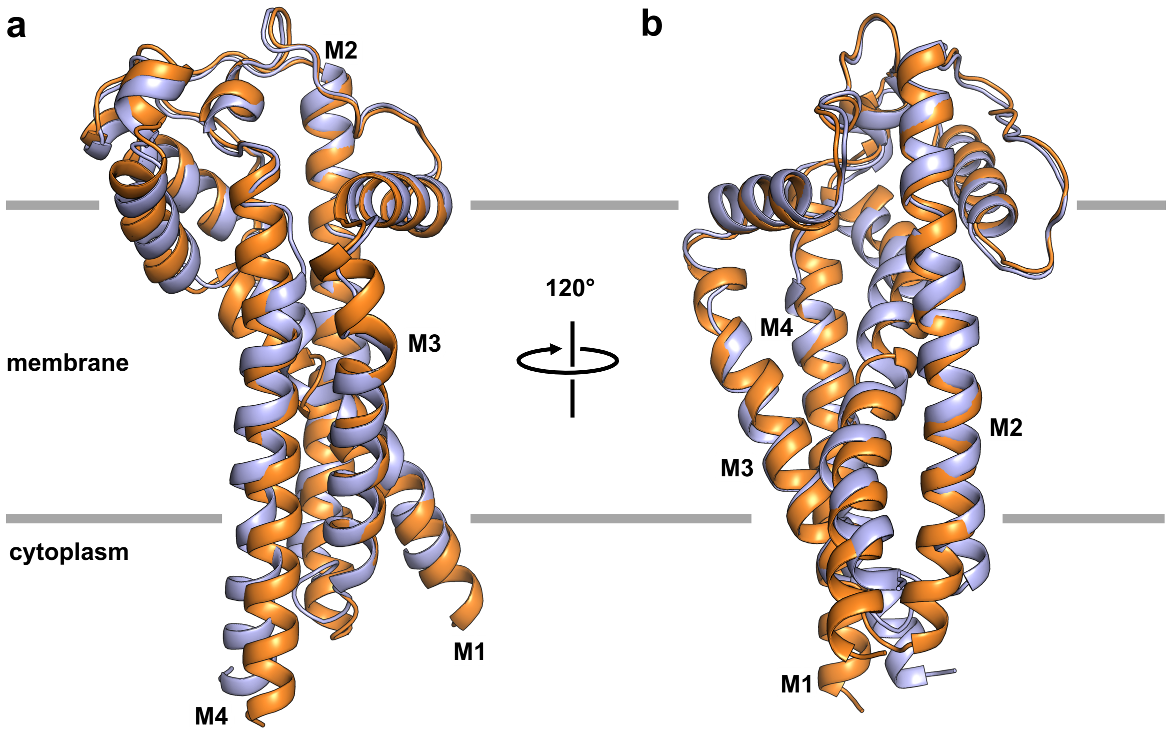

Supplementary Figure 4. Homology model of Lit2 from *L. monocytogenes*. The homology model of Lit2 from *L. monocytogenes* (light blue) is superimposed to the Lit crystal structure (orange). The model was generated using the Robetta server and the structure of Lit (MolB) as template.

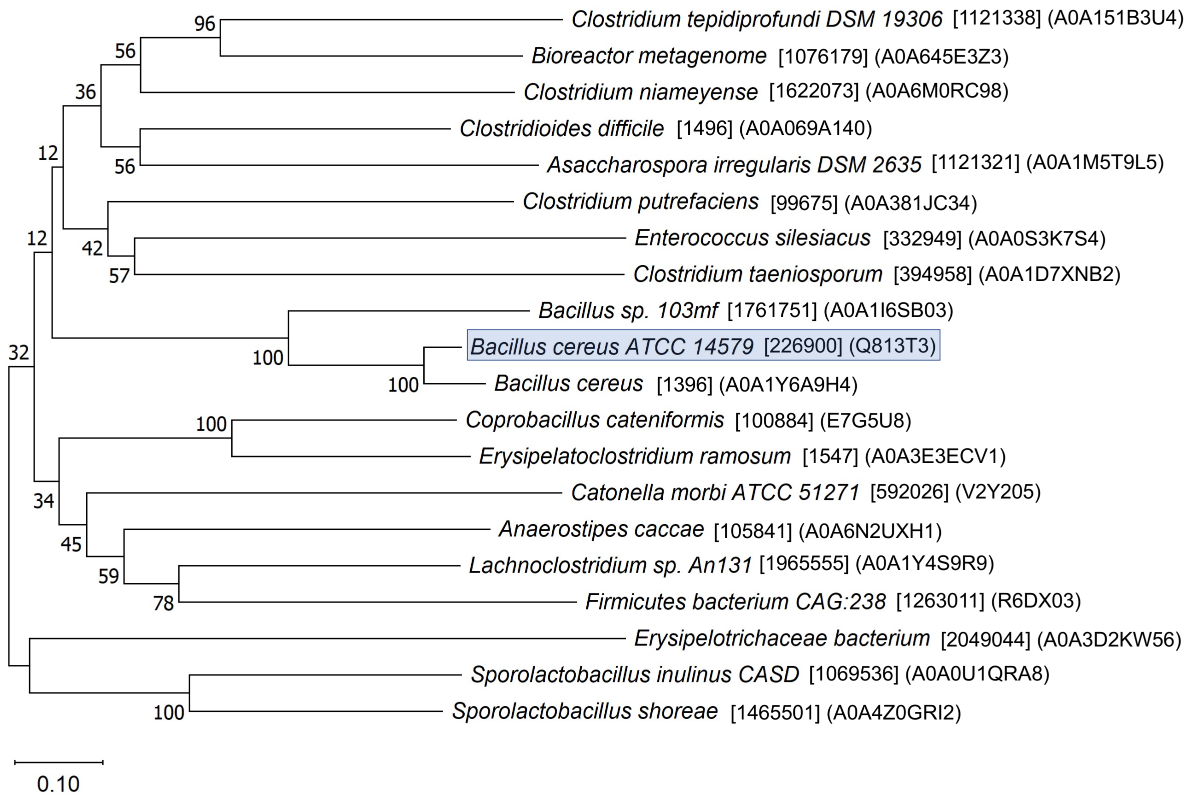

Supplementary Figure 5. Phylogenetic relationship of *Bacillus subtilis* (in blue) to other bacteria harbouring the Lit coding sequence. The phylogenetic tree was constructed from 20 non-redundant amino acid sequences from the UniProt database using MEGA X[5] with the neighbour-joining method[6]. Uniprot accession numbers for Lit orthologues are in round brackets and unique taxonomic identifiers assigned by the NCBI to the source organism of each orthologue are in square brackets. Bootstrap values from 500 replicons are shown next to the branches and represent the percentage of replicate trees in which the associated taxa clustered together. The scale bar represents the relative evolutionary distance, in number of amino acid substitutions per site, used to infer the phylogenetic tree.

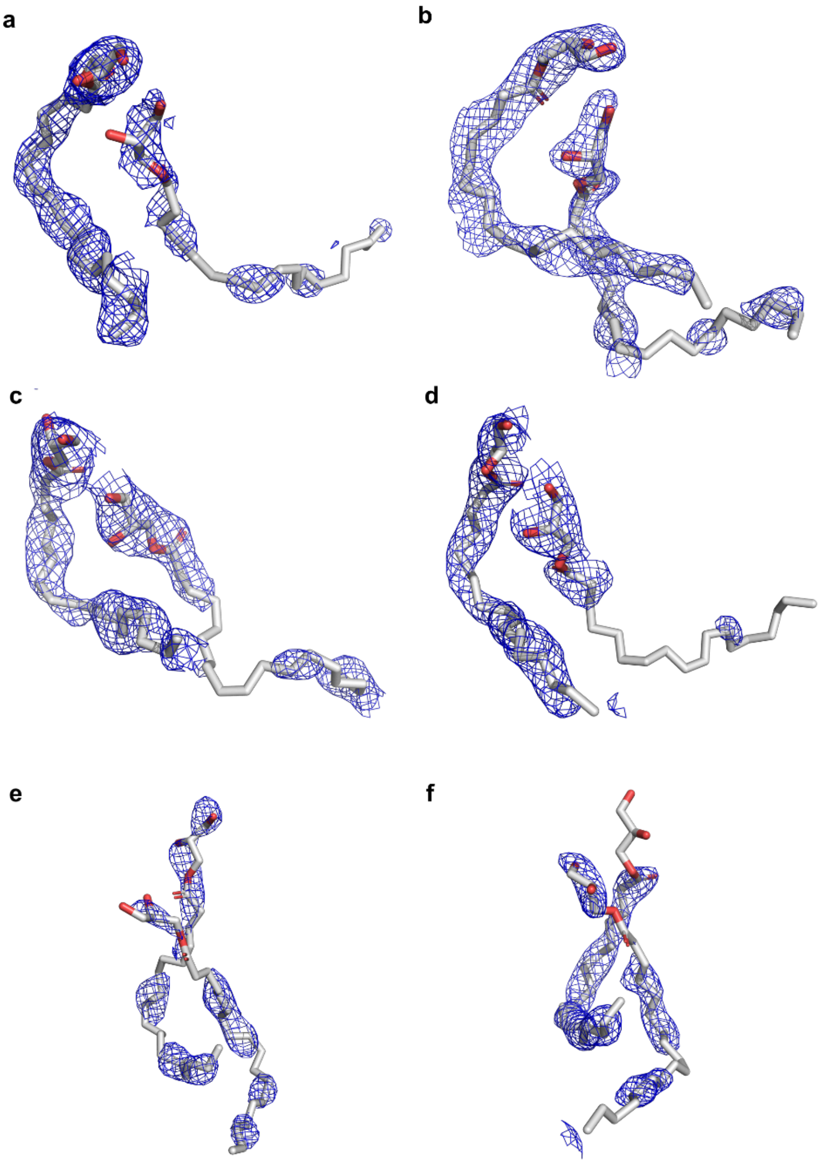

Supplementary Figure 6. Electron densities associated with the monoolein molecules in the active site of Lit wild-type and mutants. The $2F_o - F_c$ omit maps are contoured at 1.0 σ (blue net). (**a, b**) Monoolein molecules in the active site of Lit wild-type (Lit_2 structure at 1.95 Å, orthorhombic $P2_12_12$ form)**.** (**c,d**) Monoolein molecules in the active site of Lit wild-type (Lit_1 structure at 2.27 Å, monoclinic $P2_1$ form)**.** For Lit_1 and Lit_2, the electron densities are shown for molecule A (**a, c**) and for molecule B (**b,d**) of the asymmetric units. (**e**) Monoolein molecules in the active site of the Lit H85A mutant (LitH85A structure at 2.43 Å, monoclinic $C2_1$ form)**.** (**f**) Monoolein molecules in the active site of Lit H85R mutant (LitH85A structure at 2.2 Å, monoclinic $C2_1$ form)**.**

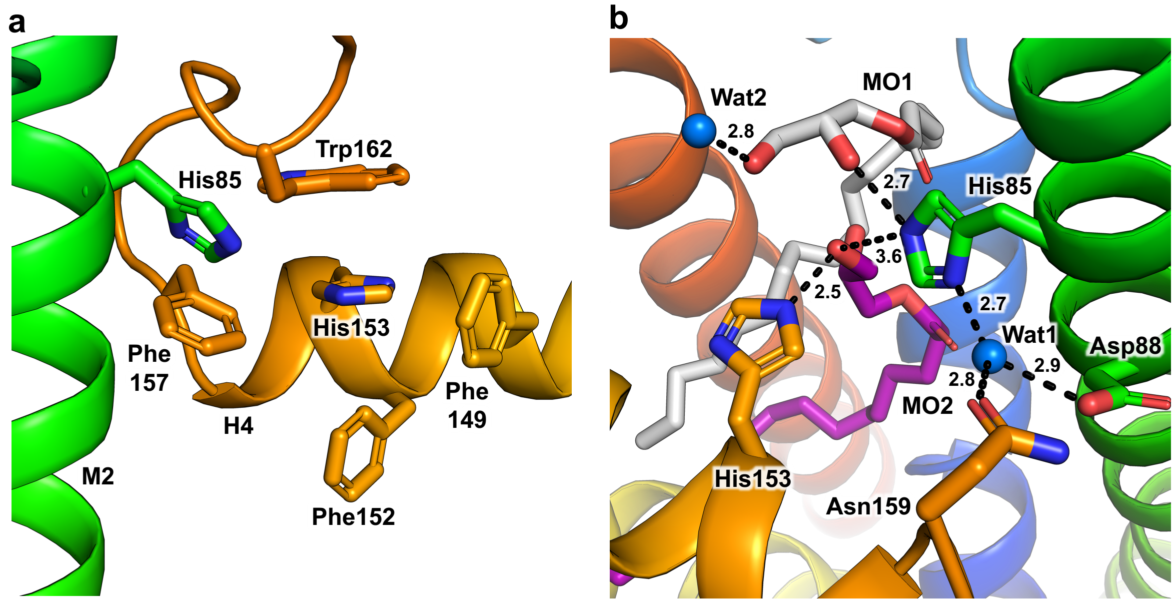

Supplementary Figure. 7. Views of catalytic histidines and their surroundings in Lit. (**a**) View into the active site of catalytic histidines, His85 and His153, surrounded by conserved aromatic residues. The neighbouring aromatics put the catalytic dyad in a special environment; one that can at once interact favourably with charged and uncharged histidines. (**b**) The two catalytic histidines are within 5 Å of one another and both hydrogen bond with the C3 primary hydroxy of monoolein molecule MO2. His85 hydrogen bonds with the C2 hydroxy of monoolein MO1 and with water molecule Wat1 . Wat1, in turn, is hydrogen bonded to Asp88 and Asn159. Distances shown as dashed lines are in ångströms.

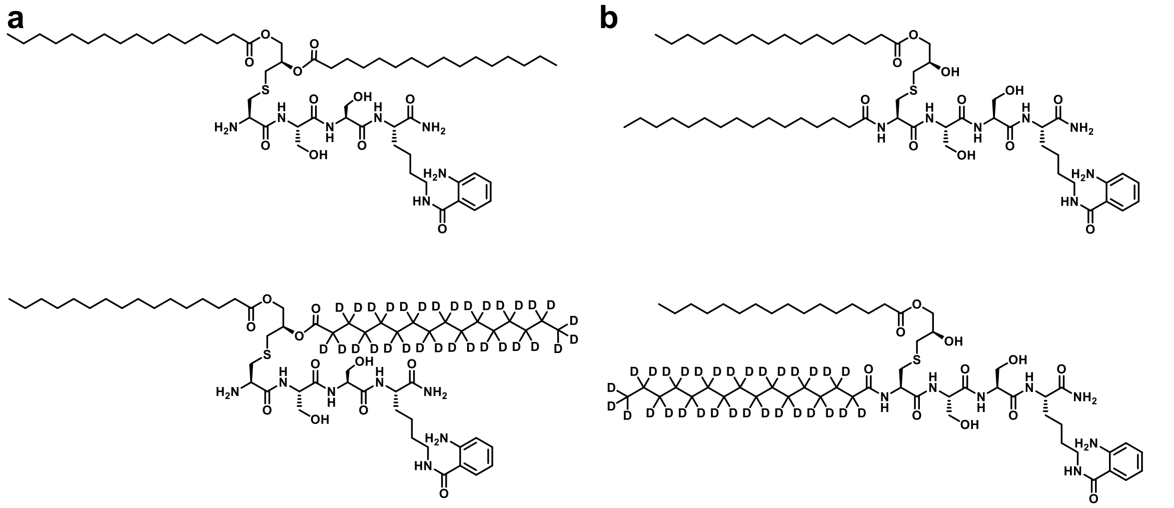

Supplementary Figure 8. Synthetic protiated and deuterated fluorescent lipopeptides used to assay Lit transacylase activity. (**a**) Substrate. Top, protiated. Bottom, deuterated. (**b**) Product. Top, protiated. Bottom, deuterated. The lipopeptide has a dipalmitoylated cysteine at the N-terminus followed by two serines and a C-terminal lysine amino-benzylated at its ε-amino group. The stereoform at the C2 position of the dipalmitoyl group is (*R*).

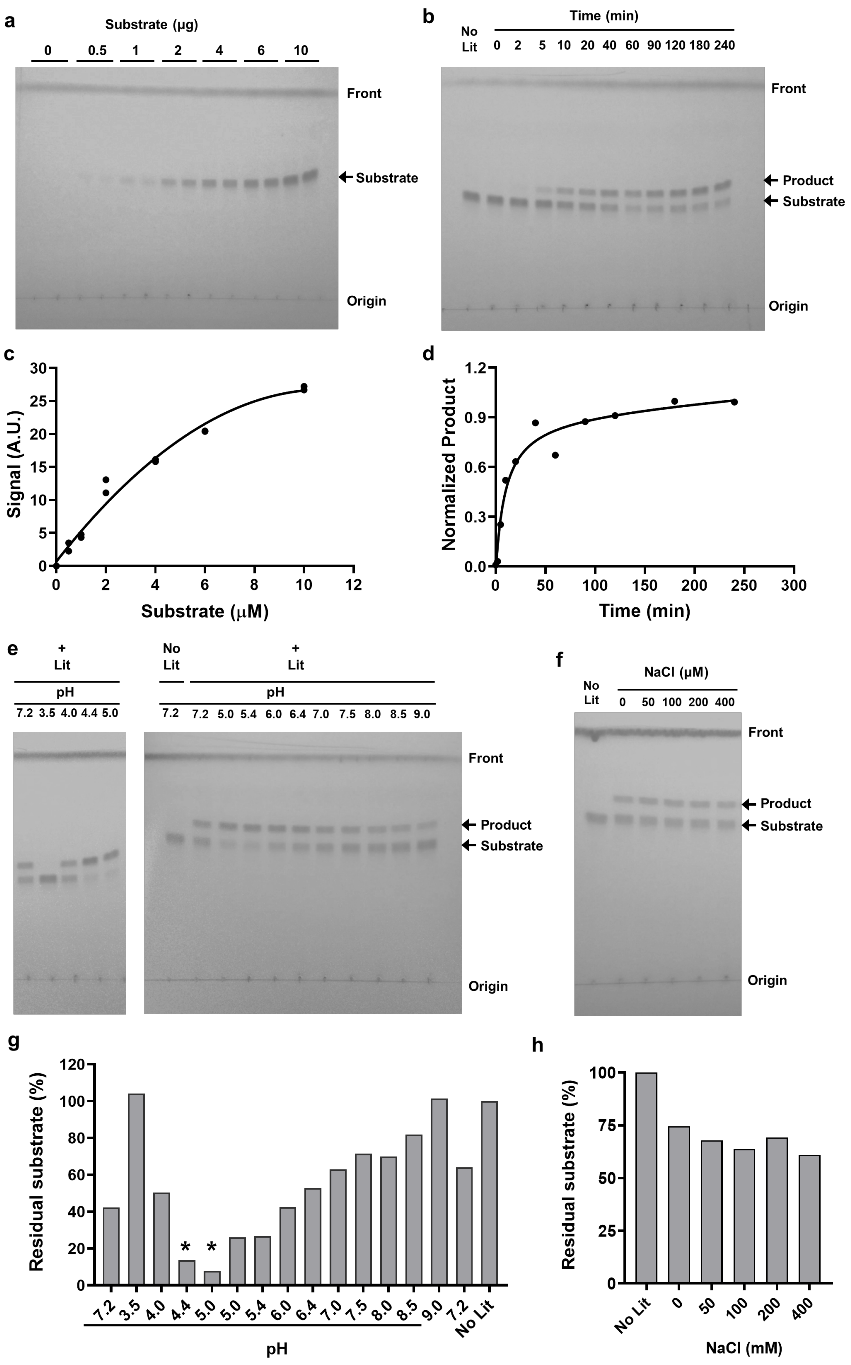

Supplementary Figure 9. Thin layer chromatographic assay of Lit transacylase activity with the fluorescent lipopeptide substrate (*R*)-DAG-CSSK-Abz (FP2). (**a,b**) Calibration of the TLC method where increasing amounts of the substrate (in duplicate) were loaded on the plate. Fluorescence was detected as described under Methods and quantified by densitometric analysis of the data in **a** using ImageJ. The response curve shown in **b** was generated using Graphpad Prism 8 and is drawn to guide the eye. (**c,d**) Progress curve of the Lit reaction. The substrate and product are well separated chromatographically and can be quantified individually by densitometric analysis. (**e,f**) Dependence of Lit reaction rate on pH. Two TLC plates were used to cover the pH range of interest from pH 3.5 to pH 9.0. The plate labelled **e** (left) included pH values 3.5 to 5.0. Plate **e** (right) included pH values from 5.0 to 9.0. The disparity in the substrate signal observed at pH 5.0 on the two plates (asterisks in panel **f**) likely reflects a differential response of the two plates to loading, position on the plate and to image analysis. A scaling factor could have been used to normalize the two sets of data.

However, we have chosen not to do so; rather to show the actual data as recorded. Clearly on plate **e** (left), Lit activity rises on going from pH 3.5 to pH 5.0. On plate **e** (right), Lit activity rises on going from pH 9.0 to pH 5.0 - 5.4. This is consistent with Lit having an optimum pH in the range from 4.4 to 5.4. The value chosen for subsequent assays was pH 5.4. (**g,h**) Dependence of Lit reaction rate on sodium chloride concentration. Activity reduced slightly with salt concentration in the range studied. The value chosen for subsequent assays was 200 mM sodium chloride.

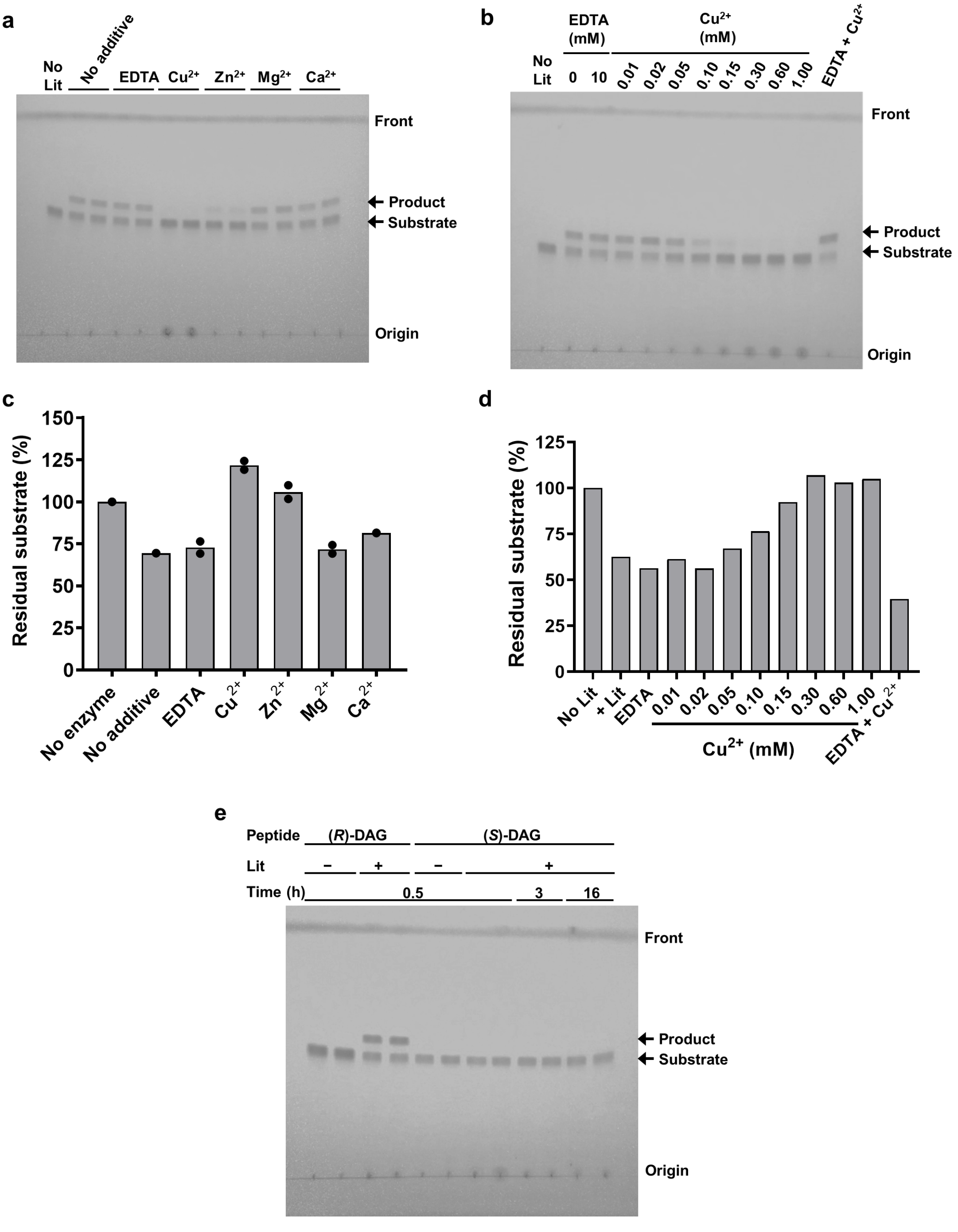

Supplementary Figure 10. Dependence of Lit activity on metal ions and on the DAG stereochemistry of the fluorescent lipopeptide substrate DAG-CSSK-Abz. Assays were performed as described under Methods and in the legend to Supplementary Fig. 9. (**a,b**) Lit activity as influenced by EDTA, copper, zinc, magnesium and calcium. (**c,d**) Lit activity as influenced by copper concentration. Assays were performed at 10 μM Lit and 150 μM substrate. (**e**) Activity of Lit with (*R*)-DAG-CSSK-Abz and (*S*)-DAG-CSSK-Abz as substrate. Approximately 70% conversion to product took place within 30 min with (*R*)-DAG-CSSK-Abz. No conversion was detected with (*S*)-DAG-CSSK-Abz after 16 h.

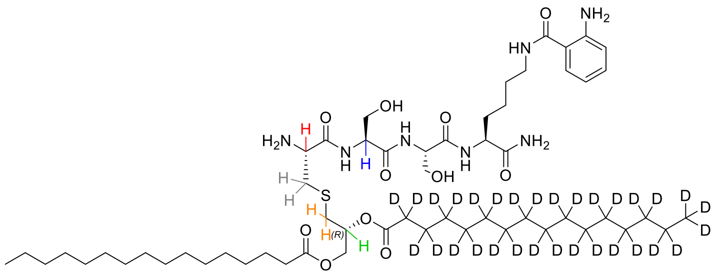

(3)

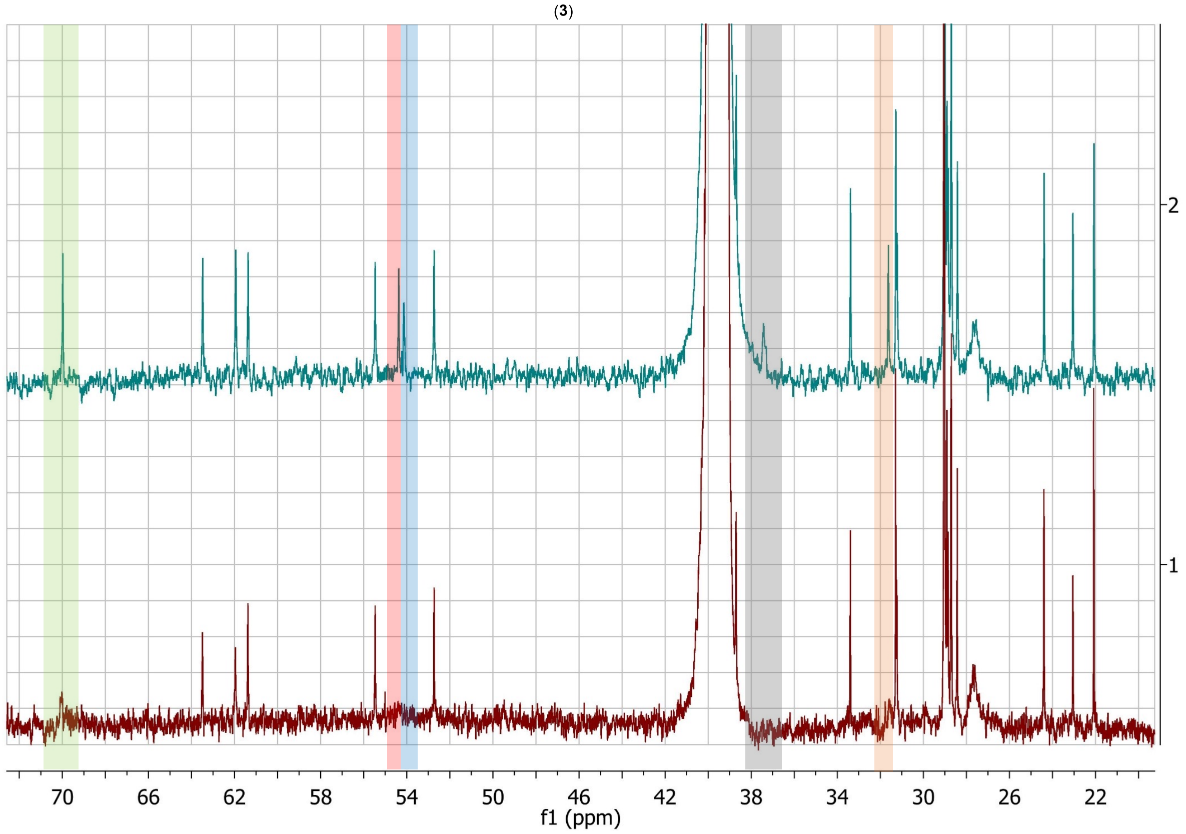

Supplementary Figure 11. Expanded view of the ^{13}C NMR (151 MHz, DMSO-d_6) comparison of dFP2 in the absence (teal, top) and presence (red, bottom) of Cu(II). Protons are color-coded by type in the chemical structure and regions in the spectra. Because Cu(II) is paramagnetic, it can broaden and induce shifts in the backbone and side chain ^{1}H and ^{13}C NMR resonances from residues it interacts in proteins (*105*). Broadening can be to the extent that the resonance signal 'disappears' completely upon direct binding. This property of Cu(II) was used here to investigate the possibility that copper binds at the N-terminus of DA-BLPs. To this end, the effect of Cu(II) addition on the ^{1}H and ^{13}C NMR resonances of the DA-lipopeptide, dFP2, was examined. The addition of $CuSO_4$ (0.1 equivalents) to dFP2 in DMSO-d_6 resulted in quenching of five ^{13}C signals from the N-terminal end of the peptide indicating proximity of these nuclei to Cu(II). The affected residues, S-glyceryl-CH (green, 70.0 ppm), Ser^{+2}-αC (purple, 54.5 ppm), Cys^{+1}-αC (red, 54.2 ppm), Cys^{+1}-βC (grey, 37.4 ppm), S-glyceryl-CH_2 (orange, 31.3 ppm), are highlighted in the spectra. The full ^{13}C NMR spectrum of dFP2 following Cu(II) addition is shown in Supplementary Fig. 12. The ^{1}H and ^{1}H-^{13}C HSQC spectra (Supplementary Fig. 13 and Supplementary Fig. 14 respectively) show that the ^{1}H resonances of these residues are similarly affected, confirming a selective interaction of Cu(II) at the N-terminal region of the lipopeptide in DMSO.

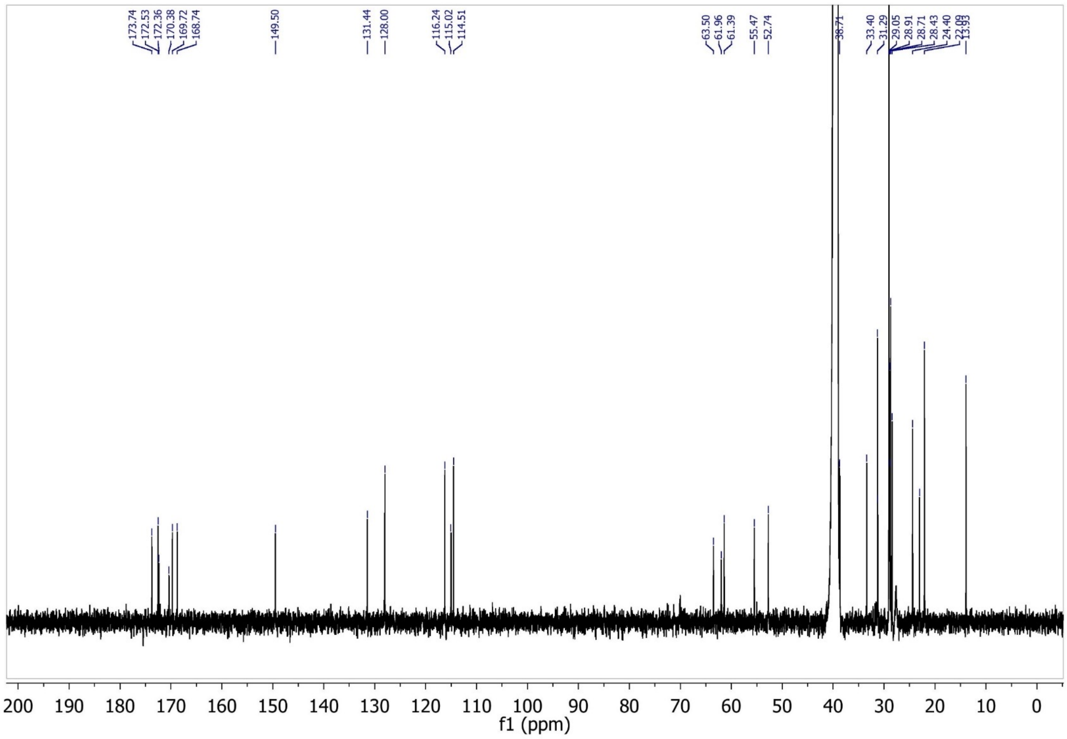

Supplementary Figure 12. ^{13}C NMR (151 MHz, DMSO-d_6) of dFP2 in the presence of Cu(II).

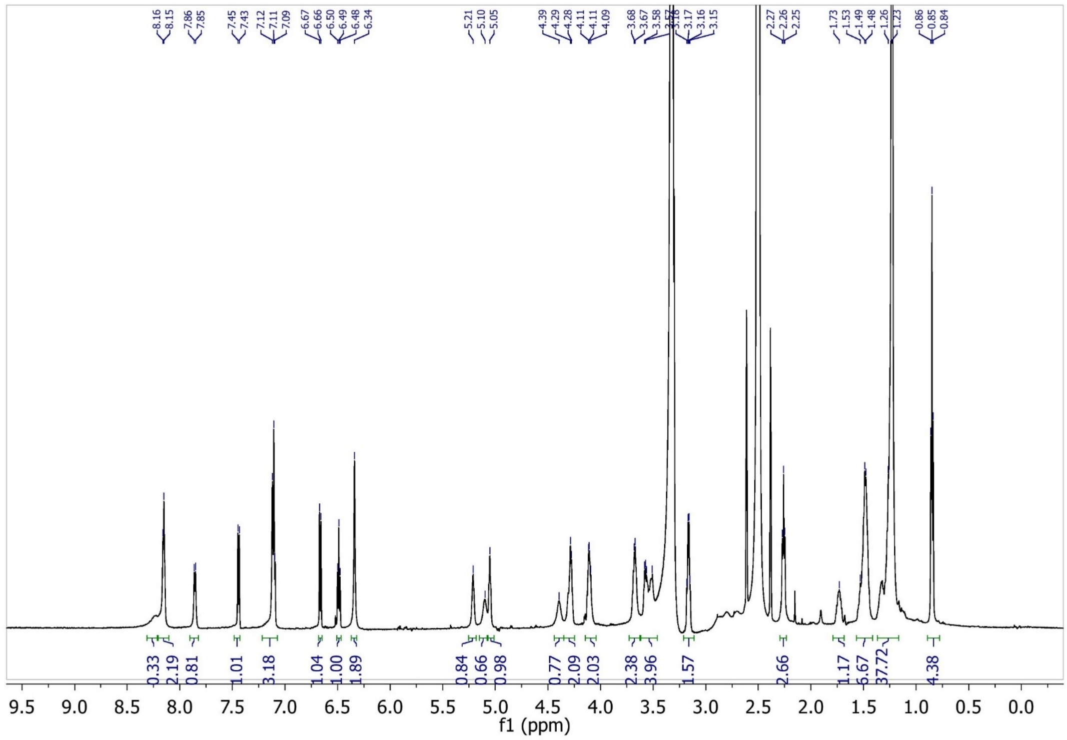

Supplementary Figure 13. ^{1}H NMR (600 MHz, DMSO-d_6) of dFP2 in the presence of Cu(II).

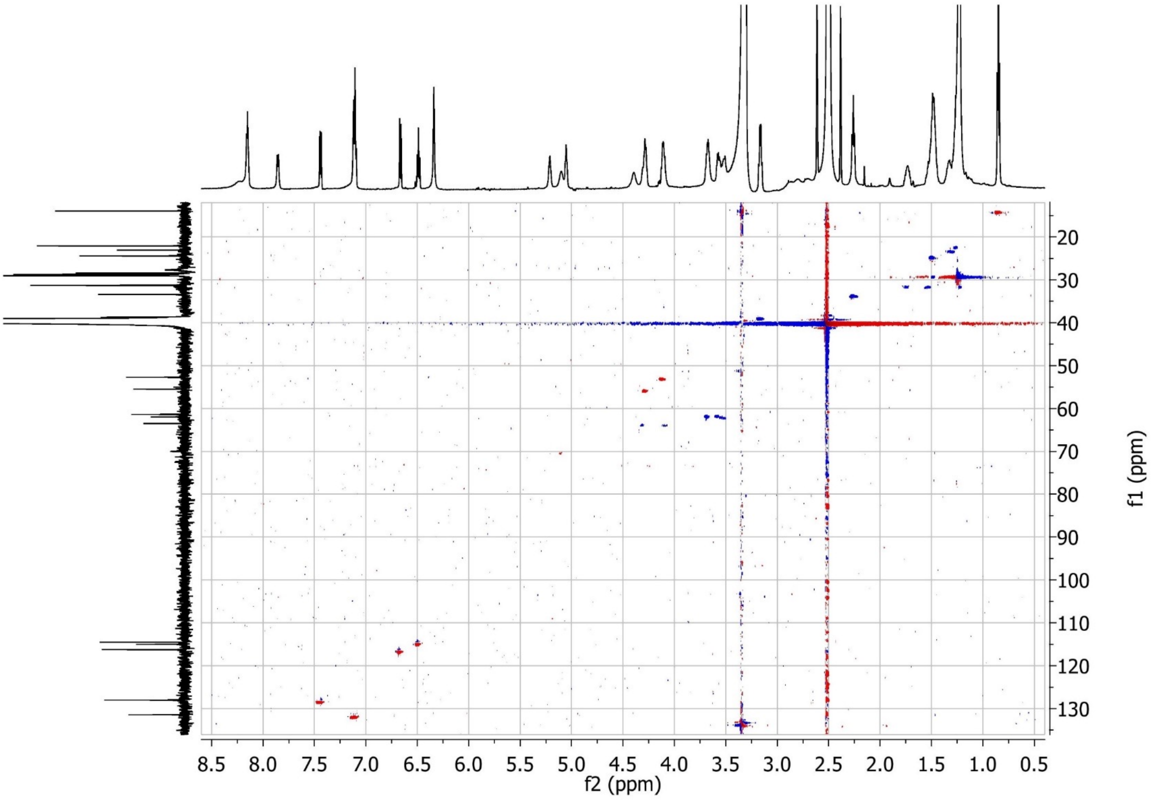

Supplementary Figure 14. HSQC NMR (DMSO-d_6) of dFP2 in the presence of Cu(II).

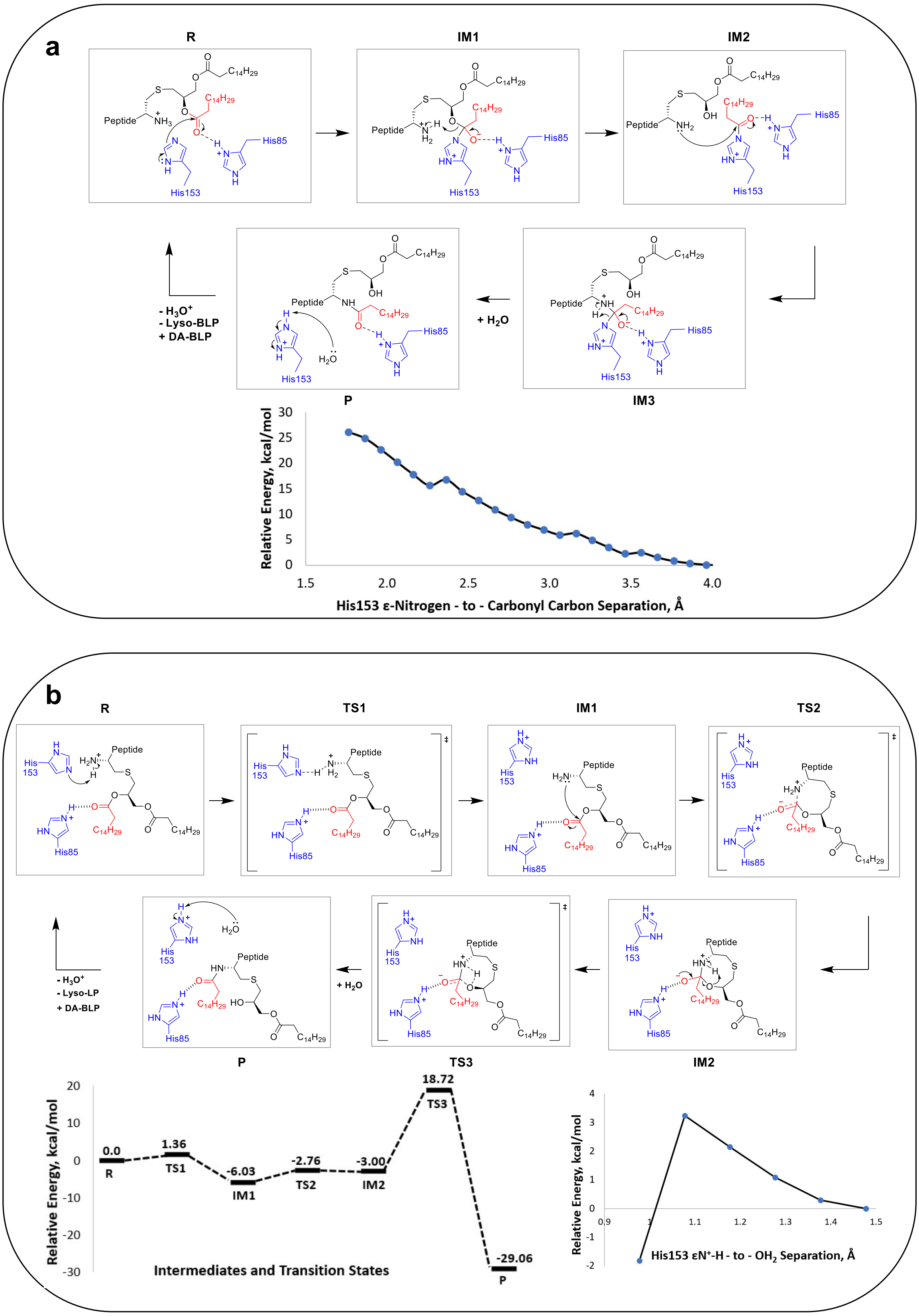

Supplementary Figure 15. Proposed Lit transacylation reaction mechanisms evaluated by QM/MM. (**a**) Transfer via an acylated enzyme intermediate. Upper panels: Reaction mechanism. The reaction takes place in five steps: **1.** The ε-nitrogen in His153 performs a nucleophilic attack at the carbonyl carbon of the substrate's *sn*-2 chain (red) resulting in the formation of a covalent link

between the substrate and the enzyme (intermediate 1, IM1). **2.** The *sn*-2 bridging ester oxygen abstracts a proton from the substrate's α-ammonium group forming acylated His153 (intermediate 2, IM2). **3.** The newly formed terminal α-amine performs a nucleophilic attack on the carbonyl carbon of the *sn*-2 chain resulting in the formation of intermediate 3 (IM3). **4.** IM3 collapses forming the lyso-lipoprotein product. **5**. The imidazolium of His153 transfers a proton to a water molecule. Lower panels: Reaction energy profile. The first step in the proposed reaction mechanism, in which the ε-nitrogen of His153 attacks the carbonyl carbon of the *sn*-2 chain, has been explored using QM/MM adiabatic mapping of the potential energy surface. The potential energy of the system was calculated as the ε-nitrogen was moved toward the carbonyl carbon in steps of 0.1 Å. The energy continued to rise reaching a value of 26 kcal/mol at a N-C separation of 1.76 Å. Throughout the process, neither an acylated intermediate nor a transition state associated with this intermediate were found, highlighting the unfeasibility of the proposed mechanism. (**b**) Direct intramolecular transfer. Upper panel: Reaction mechanism. The reaction takes place in four steps: **1**. His153 abstracts a proton from the terminal α-ammonium group of the substrate to form intermediate IM1 via transition state 1 (TS1) with an activation energy barrier of 1.36 kcal/mol. **2**. The newly formed terminal amine performs a nucleophilic attack on the carbonyl carbon of the *sn*-2 chain forming an 8-membered heterocyclic intermediate, IM2, via TS2 with an activation energy barrier of 3.27 kcal/mol. **3**. IM2 collapses to form the lyso-lipoprotein product (P). This process goes through TS3 with an activation energy barrier of 21.7 kcal/mol and is the rate-limiting step in the reaction. **4**. Recovery of Lit for another round of catalysis occurs upon deprotonation of His153 by a water with an activation energy barrier of 3.2 kcal/mol (inset). The relative energies for the reactant (R), transition states, intermediates and product were obtained from QM/MM calculations optimized with the B3LYP functional[7]. Lower panels: Left. The QM/MM profile for the Lit catalysed reaction from the DA-BLP reactant to the lyso-lipoprotein product. Right. The QM/MM potential energy scan for the deprotonation of the His153 ε-nitrogen in the lyso-lipoprotein product by a water molecule. The potential energy of the system was calculated as the proton of the ε-nitrogen was moved toward the oxygen atom of the water molecule in steps of 0.1 Å.

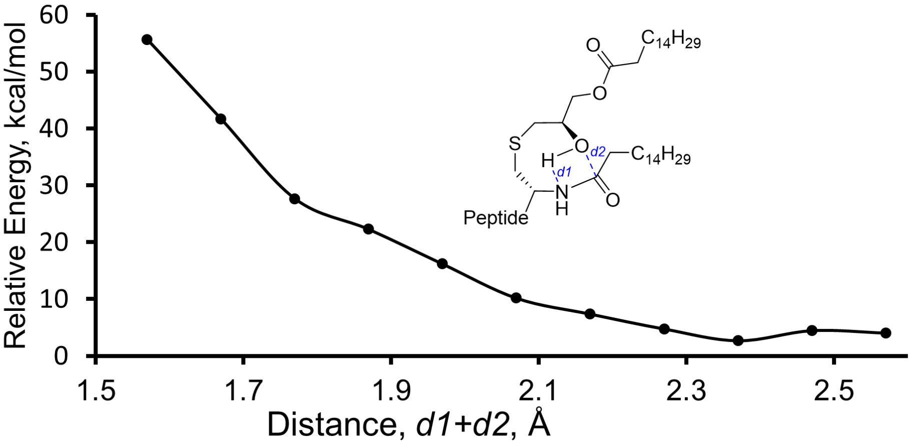

Supplementary Figure 16. Failure to obtain IM2 with unprotonated His85. The potential energy scan from the product to IM2 was conducted along the reaction coordinate defined as a linear combination of two distances, *d1* and *d2* (shown in a light blue dotted line). The calculated energy continued to rise reaching a value of 55 kcal/mol at a separation of 1.57 Å.

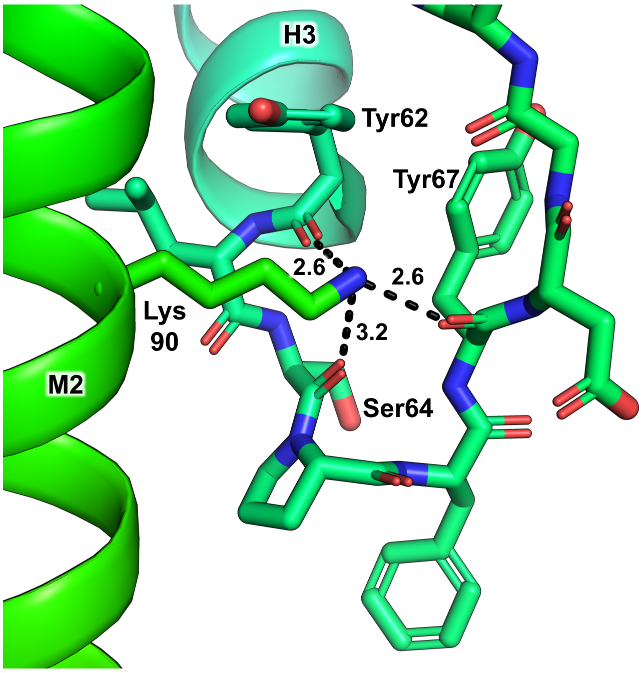

Supplementary Figure 17. Lys90 is proposed to play a role in stabilizing the structure of Lit. The ε-amino group of Lys90 coordinates with backbone carbonyls to secure the extracellular domain, ECD1, which forms part of the substrate binding pocket.

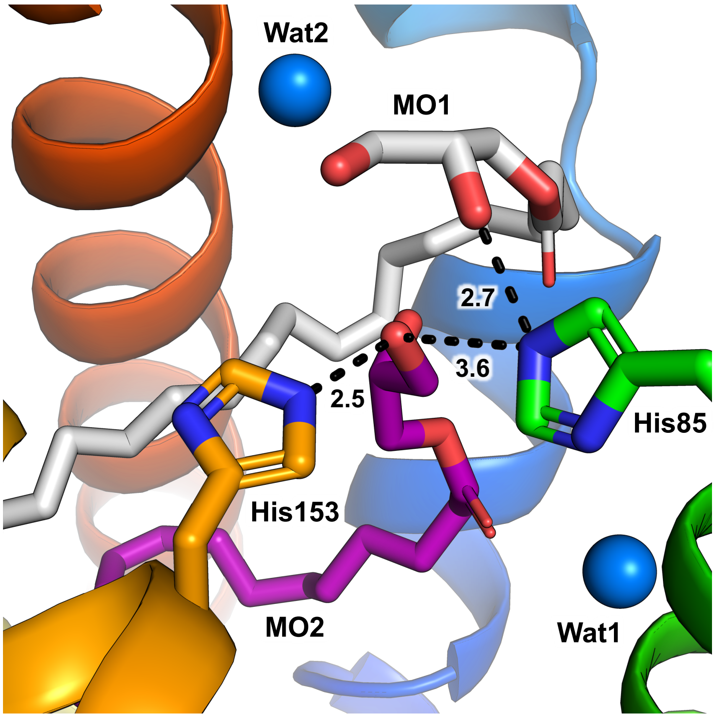

Supplementary Figure 18. Structured lipids act as surrogates for the fatty acyl chains in the lipoprotein substrate and product of the Lit reaction. The view is into the active site from the extracellular space. For clarity, the two monoolein lipids are shown with differently colored (grey, purple) carbon atoms. Bond lengths are in ångströms.

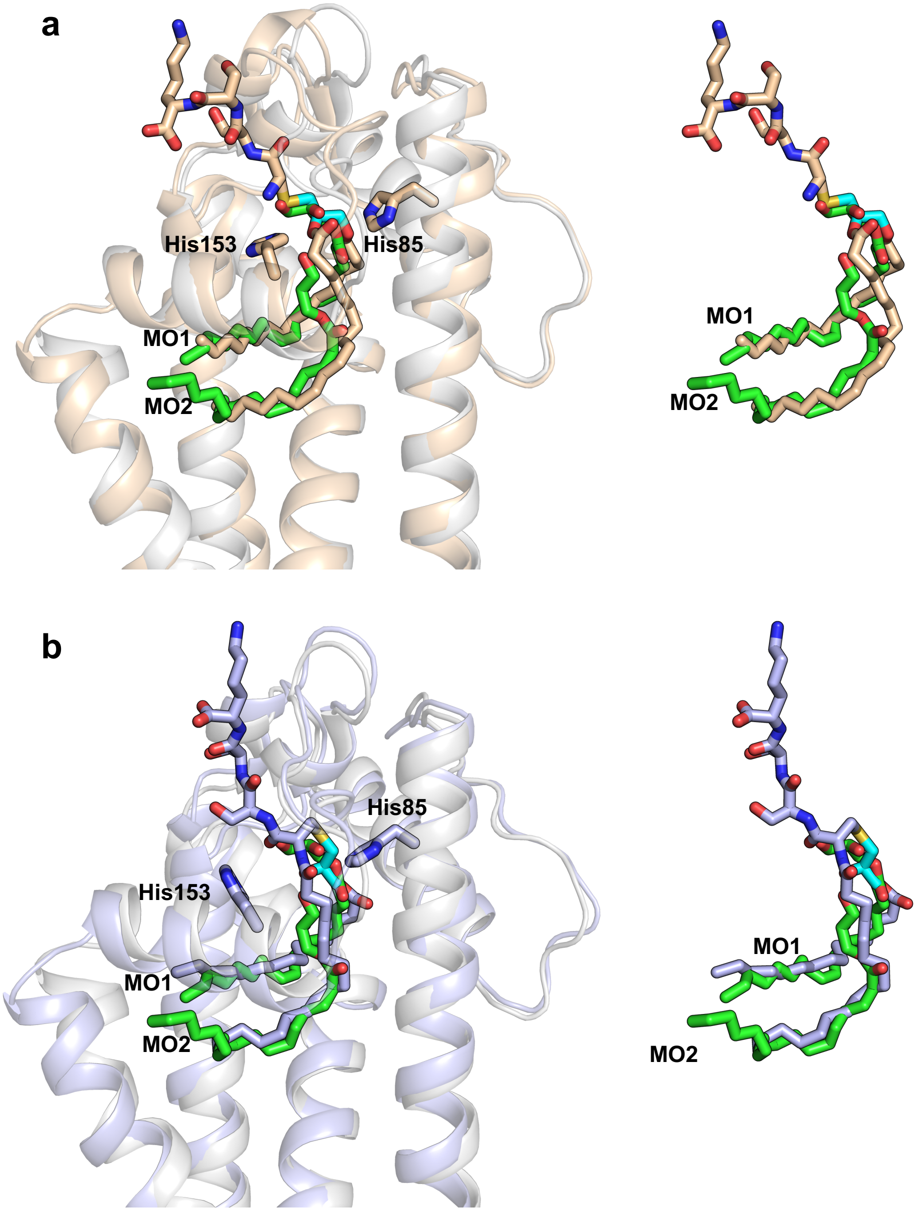

Supplementary Figure 19. Acyl chains of the substrate and product in the binding pocket of Lit obtained from MD simulations coincide with those of the monoolein molecules in the crystal structure. (**a**) Left: Overlay of the lipopeptide substrate and monoolein molecules in the Lit structure. The Lit crystal structure (grey) with bound monooleins (green acyl chain carbons, cyan glycerol carbons) was superposed on the Lit structure (light wheat) with bound substrate (wheat) obtained from MD simulations. The side chains of the two catalytic histidines are shown as fiducials. Right: Overlay of the bound substrate and monoolein molecules are shown with the protein structure omitted for clarity. (**b**) As in A showing the bound lipopeptide product. The position of the substrate and product does not undergo significant changes during the MD simulation runs. The ester group in the lipopeptide substrate and product coincide with the ester linkage in one of the bound monooleins (MO1) in the crystal structure. The hydrophobic tails of substrate and product overlap to considerable degrees with those of the two bound monooleins.

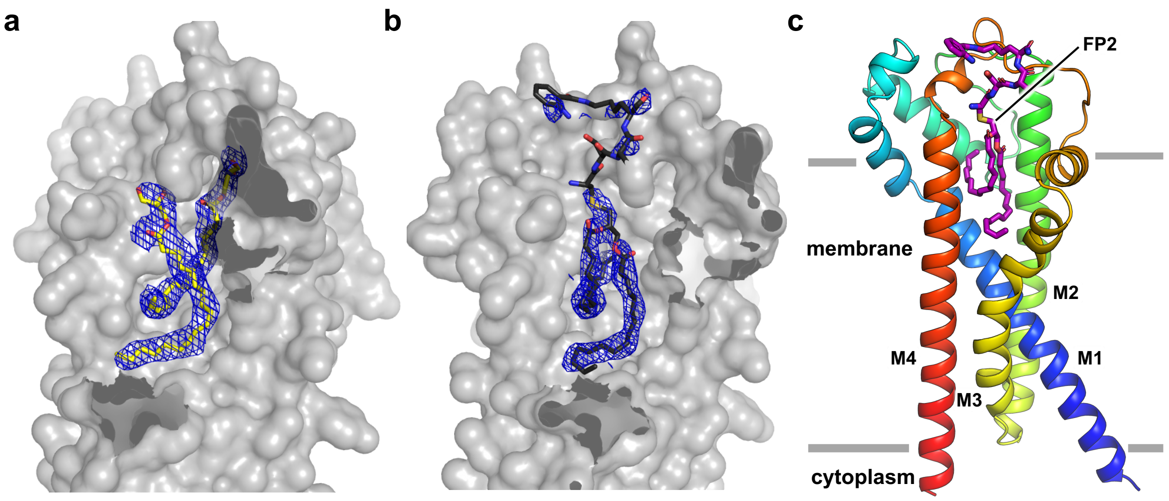

Supplementary Figure 20. Interpreting the electron density in the binding pocket of the His85Ala inactive Lit mutant crystallized with lipopeptide substrate. (**a**) Two monoolein molecules fit to the density (blue net, 2Fo-Fc map, 1 sigma) that emerged when the model was refined with two lipid molecules in the binding pocket. Lit is shown in surface representation with parts cut away to reveal structured lipids shown with yellow carbon atoms. (**b**) FP2 molecule fit to density. All else as in **a**. (**c**) A cartoon representation of the structure in **b**. It shows how the extracellular half of M3 separates from M4 to create an opening into the binding pocket of this inactive mutant form of the enzyme. Model orientation in the three panels is different and optimized to highlight particular features in each.

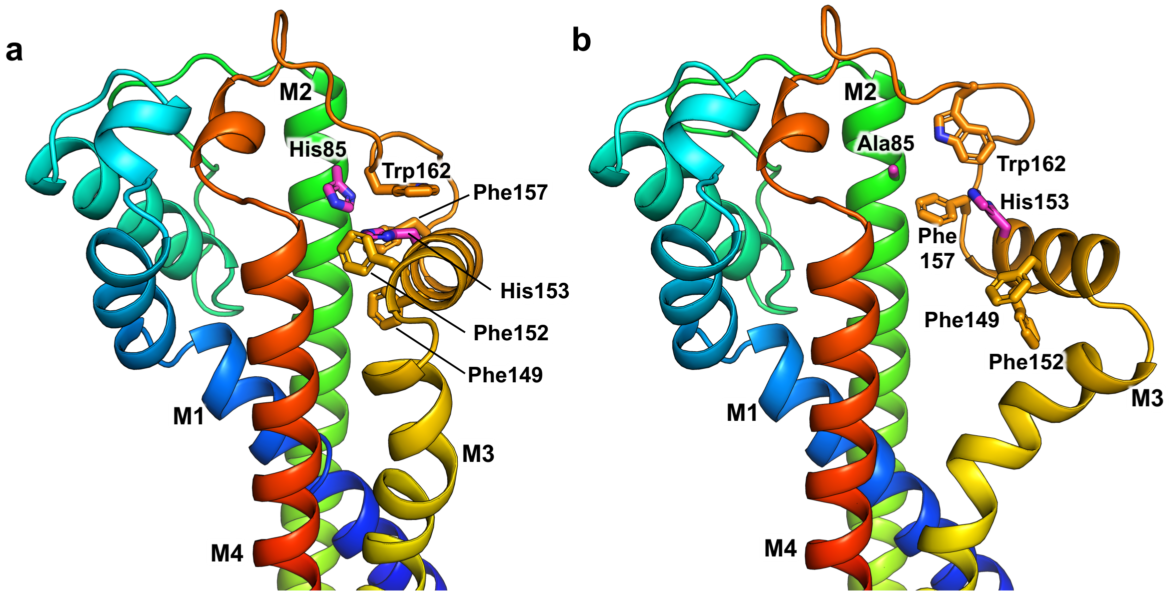

Supplementary Figure 21. Comparison of the entrance to and of conserved residues in the putative binding pocket of wild-type Lit and the His85Ala Lit mutant. The view is from the membrane into the active site. (**a**) Wild-type Lit. The conserved residues (stick representation) are clustered toward the interior of the cap domain with the catalytic histidine dyad residues (purple carbons) at the core of the cluster. The gate between the extracellular ends of M3 and M4 are in close contact effectively closing entry to and egress from the binding pocket. (**b**) His85Ala Lit. The extracellular end of M3 has moved away from M4 to open the gate. The conserved residues are no longer clustered toward the interior of the protein as in wild-type Lit. This mutant form, whilst not physiologically relevant, may provide insights regarding the structural changes the enzyme can undergo to enable substrate binding and product release. A similar structural flexibility was observed in the MD simulations (Supplementary Fig. 22, Supplementary Movies 2-4).

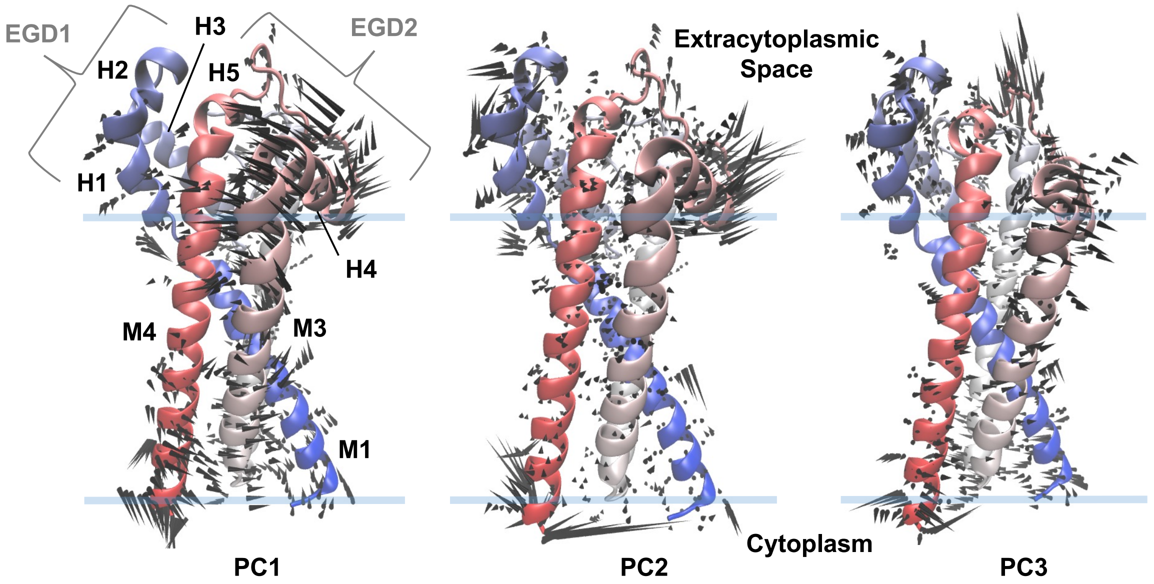

Supplementary Figure 22. Direction of motion in parts of Lit detected by principle component analysis (PCA) of MD simulations trajectories suggest a route into and out of the active site. The three lowest frequency modes of the empty form of Lit from the PCA of the MD simulations trajectories show considerable outward and inward movements of the extracellular half of M3 and upward and downward movements of EGD2 (H4, H5) from the helical core. A potential pathway by which substrate enters and product leaves the enzyme is via the fenestration between the extracellular halves of M3 and M4. The projection of three principle components (PC1, PC2, PC3) is shown using porcupine plots. The black arrowheads highlight the direction of motion. The three directions of motion have been captured in Supplementary Movies 2-4.

Supplementary Figs. 23 and 24 show the ^{1}H NMR spectra of protiated and deuterated Lit substrates and products, respectively**.** Supplementary Figs. 25 and 26 show and compare the ^{1}H NMR spectra of protiated and deuterated Lit product. Supplementary Figs. 27-34 show the ^{1}H-^{13}C HSQC spectrum of each substrate and product.

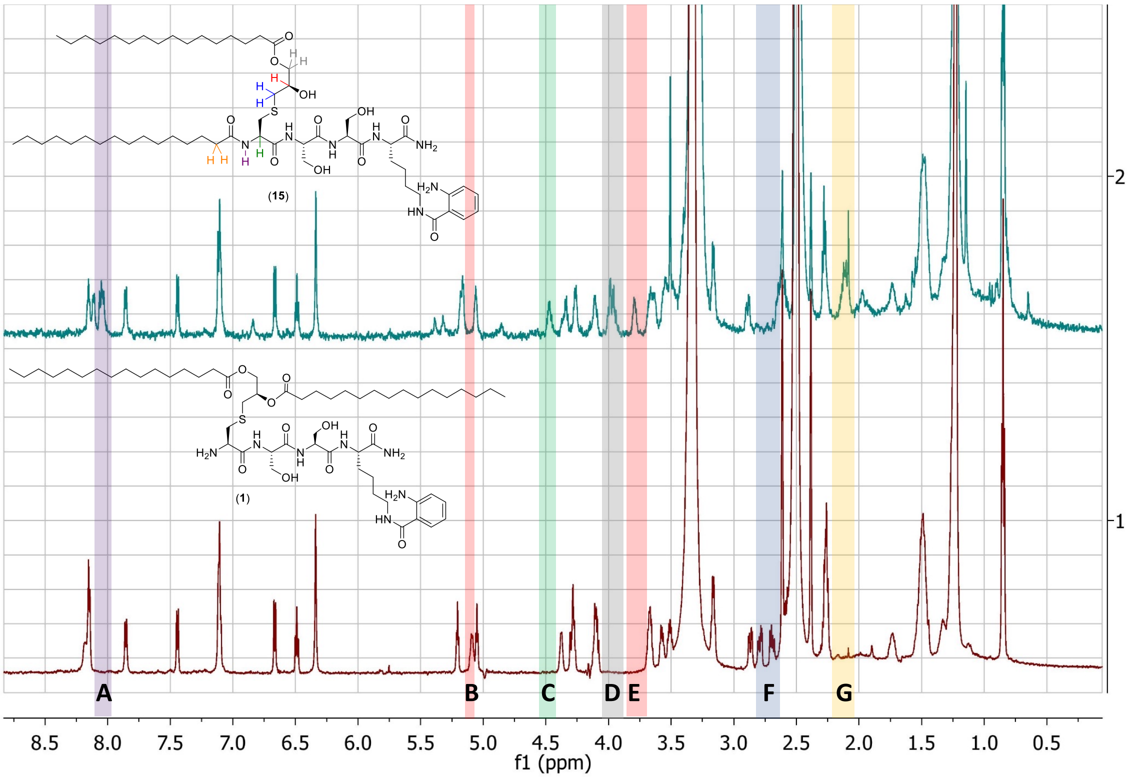

Supplementary Figure 23. ^{1}H NMR (600 MHz, DMSO-d_6) comparison of protiated product lyso-FP2 (blue, top) and protiated substrate FP2 (red, bottom). The changes in these ^{1}H NMR spectra are consistent with Lit transferring the acyl chain at the *sn*-2 of the glyceryl moiety to the free amino group of the lipopeptide. In what follows, different regions in the spectra are examined with a view to providing evidence in support of this conclusion. Protons are colour-coded by type in the chemical structure and regions (A through G) in the spectra. **Region A**: A new C(O)NH̲ signal (purple) at 8.06 ppm confirms that the *N*-terminus of the product is acylated. **Region B + E**: The signal from glyceryl-CH̲ (red) is shifted significantly upfield from 5.10 ppm to 3.80 ppm in the product as a result of deacylation of the adjacent *sn*-2 ester. **Region C**: The signal from Cys-αCH̲ (green) is shifted downfield from 3.40 ppm (concealed here by H_2O) to 4.48 ppm in the product due to acylation of the neighbouring amine in the product. **Region D**: The signal from glyceryl-O-C$\underline{H_2}$ (grey) has shifted slightly upfield from 4.30 ppm and 4.11 ppm to 3.98 ppm in the product due to removal of the *sn*-2 ester (4 bonds away). This upfield shift of 0.32 ppm is not large enough to signify deacylation of the adjacent *sn*-1 position. Note that this CH_2 group in the substrate appears as two signals, one at 4.30 ppm, the other at 4.11 ppm. The signals in the substrate overlap with the resonances of Ser-αCH (4.30 ppm) and Lys-αCH (4.11 ppm). **Region F**: The glyceryl-S-C$\underline{H_2}$ (blue) signal is shifted slightly upfield from 2.80 ppm and 2.70 ppm to 2.63 ppm in the product due to removal of the *sn*-2 ester (4 bonds away). Note that in the product, this CH_2 signal overlaps with the residual non-deuterated DMSO NMR solvent satellite peak which appears as a sharp peak at 2.62 ppm. **Region G**: A new signal appears at 2.12 ppm in the product. This signal arises from the α-CH_2 group (orange) of the acyl chain now amide-linked to the N-terminus. Unlike in the substrate, this α-CH_2 signal is not equivalent to the *sn*-1 ester α-CH_2 group.

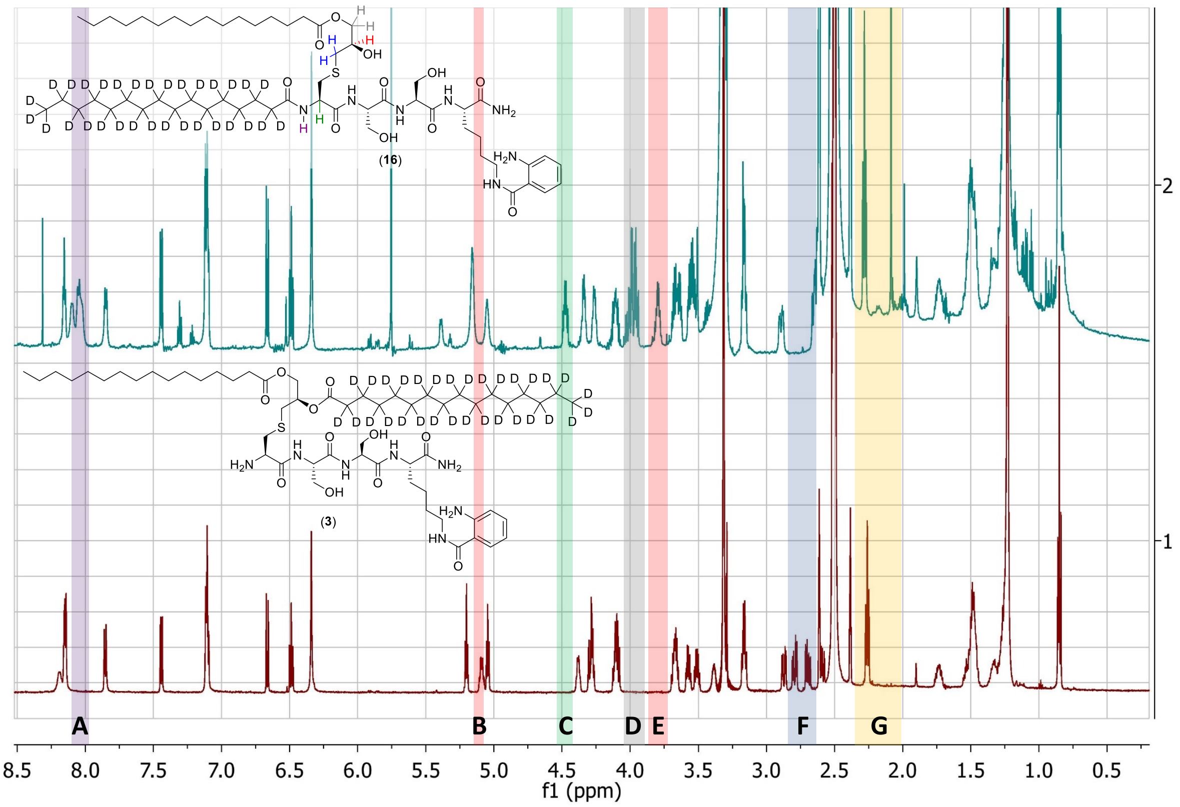

Supplementary Figure 24. ^{1}H NMR (600 MHz, DMSO-d_6) comparison of deuterated product lyso-dFP2 (blue, top) and deuterated substrate dFP2 (red, bottom). As with the protiated analogue (Supplementary Fig. 23), the differences between these two ^{1}H spectra, highlighted in regions A-G, are consistent with deacylation at the *sn*-2 position of the substrate and formation of an amide bond at the N-terminus of the product.

Due to the distinct electronic environments of the ester- and amide-linked acyl chains in the product, the characteristic α-CH_2 chemical shifts of the ester- and amide-linked acyl chain can be used to determine if a deuterated or protiated acyl chain is bound to the N-terminus. The signal in region G (2.27 ppm) belongs to the α-CH_2 group of the ester-linked *sn*-1 acyl chain. This signal has not changed between the substrate and product. This means that the ester-linked α-CH_2 group of the *sn*-1 acyl chain is in the same electronic environment in both the substrate and the product. Therefore, in the course of the Lit reaction it has remained in place and has not shifted to form the terminal amide bond in the product. As shown in Supplementary Figs. 25 and 26, all ^{1}H resonances in the deuterated product, with the exception of the signal at 2.12 ppm, are consistent with that of the protiated product, verifying that acyl transfer has occurred. However, the lack of a signal at 2.12 ppm means that the amide-linked acyl chain does not have an α-CH_2 group, indicating that the amide-linked acyl chain contains a ^{1}H NMR silent α-CD_2 group. As the deuterated acyl chain originates from the *sn*-2 position in the substrate, this result shows that the *sn*-2 acyl chain is transferred in the Lit reaction.

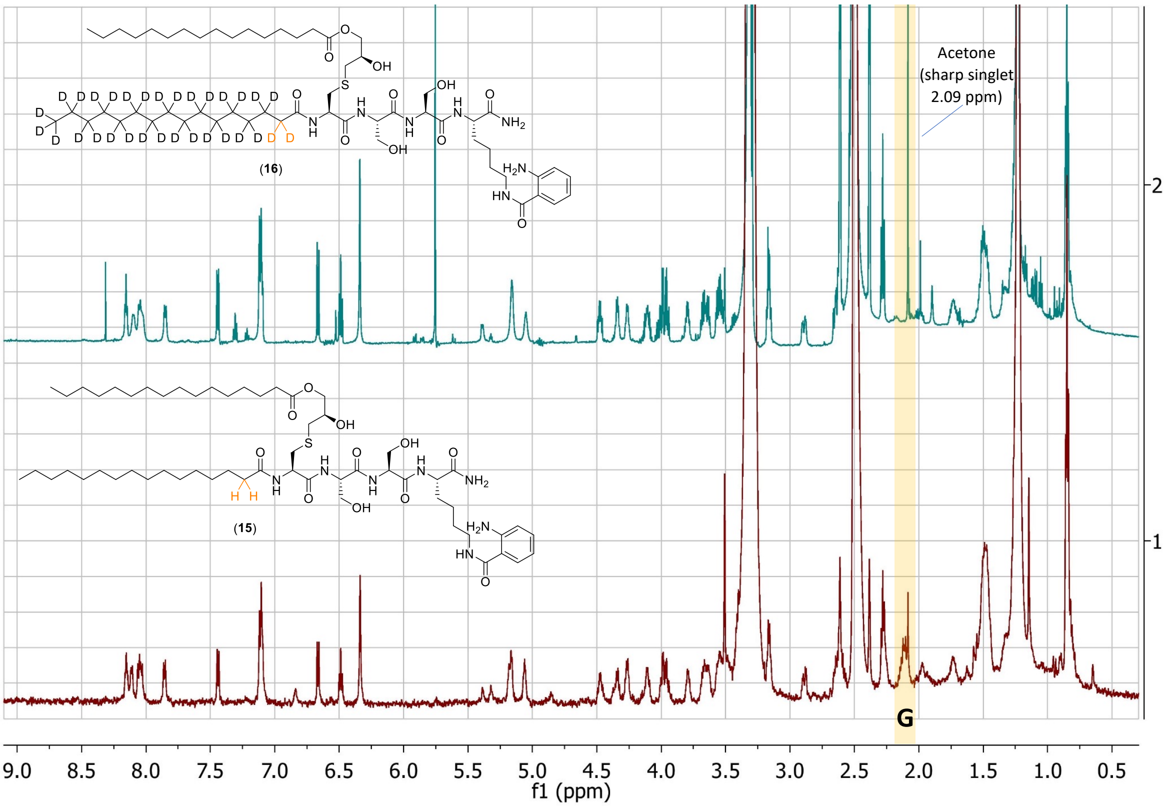

Supplementary Figure 25. Comparison of the ^1H NMR (600 MHz, DMSO-d_6) of the deuterated product lyso-dFP2 (blue, top) and the protiated product lyso-FP2 (red, bottom). A notable difference in the spectra occurs at 2.12 ppm (highlighted). This signal arises in the protiated product from the α-CH_2 of the amide linked acyl chain. In the deuterated product the amide linked acyl chain contains an α-CD_2 group which does not produce a signal in ^1H NMR.

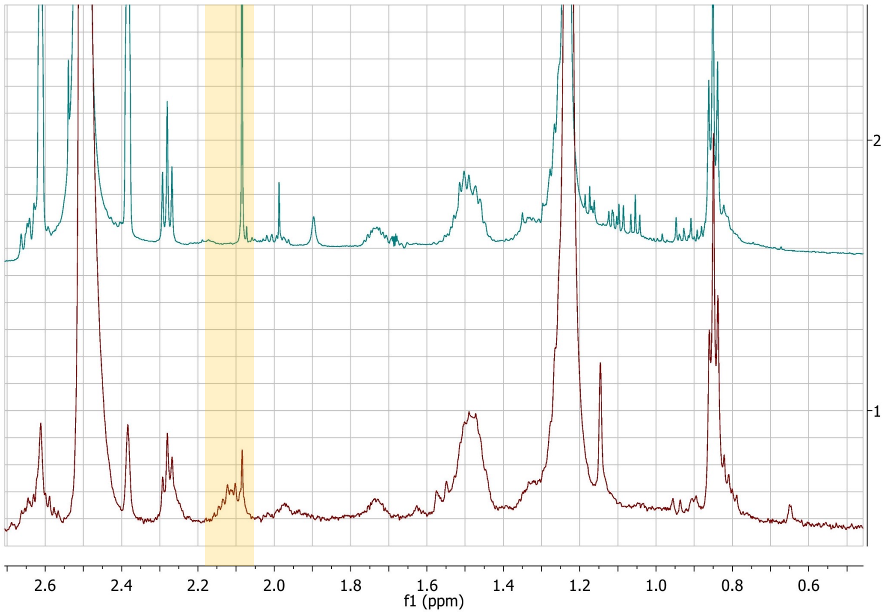

Supplementary Figure 26. Expanded view of the spectra in Supplementary Fig. 25 in the vicinity of the resonance signals at ~2.12 ppm.

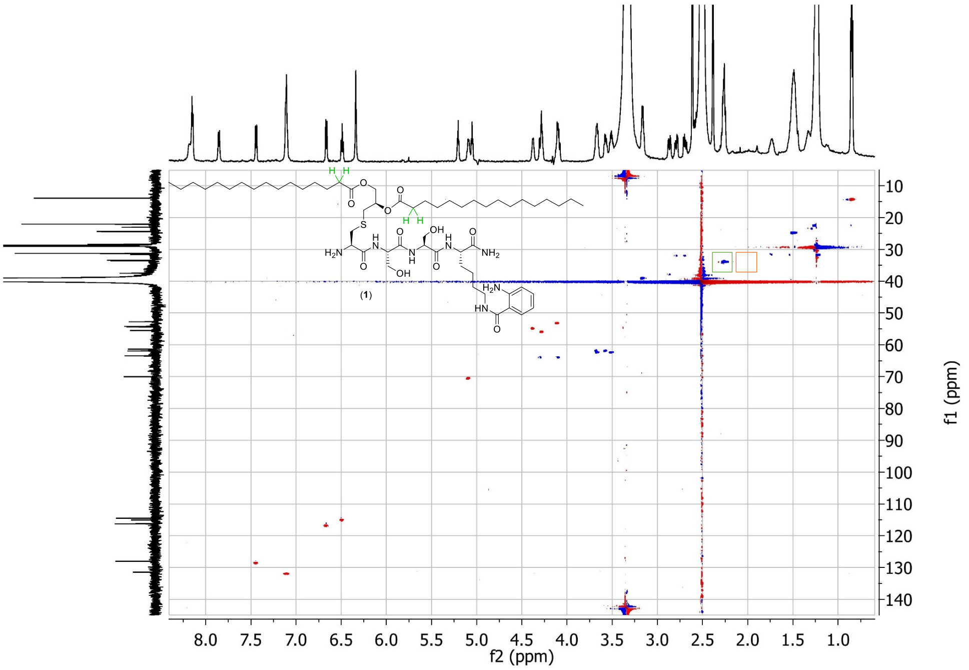

Supplementary Figure 27. ^1H-^{13}C HSQC of protiated substrate FP2. ^1H-^{13}C HSQC is a 2-dimensional NMR experiment with ^1H chemical shifts displayed on the x-axis and ^{13}C chemical shifts on the y-axis. Each HSQC signal represents a unique hydrogen which is bound to a carbon. The number of hydrogens bound to the carbon will determine the type of signal – hydrogens which are part of a CH_2 group will produce a blue signal while CH and CH_3 groups will produce a red signal. The four hydrogens (green) of the two ester α-CH_2 groups in FP2 are chemically equivalent. As "CH_2" groups, they appear as a single blue signal (green box).

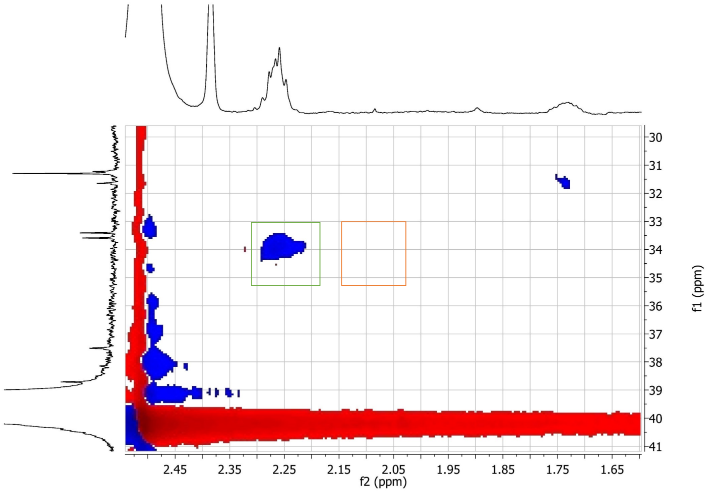

Supplementary Figure 28. Expanded view of Supplementary Fig. 27 showing the single signal arising from two ester α-CH_2 groups.

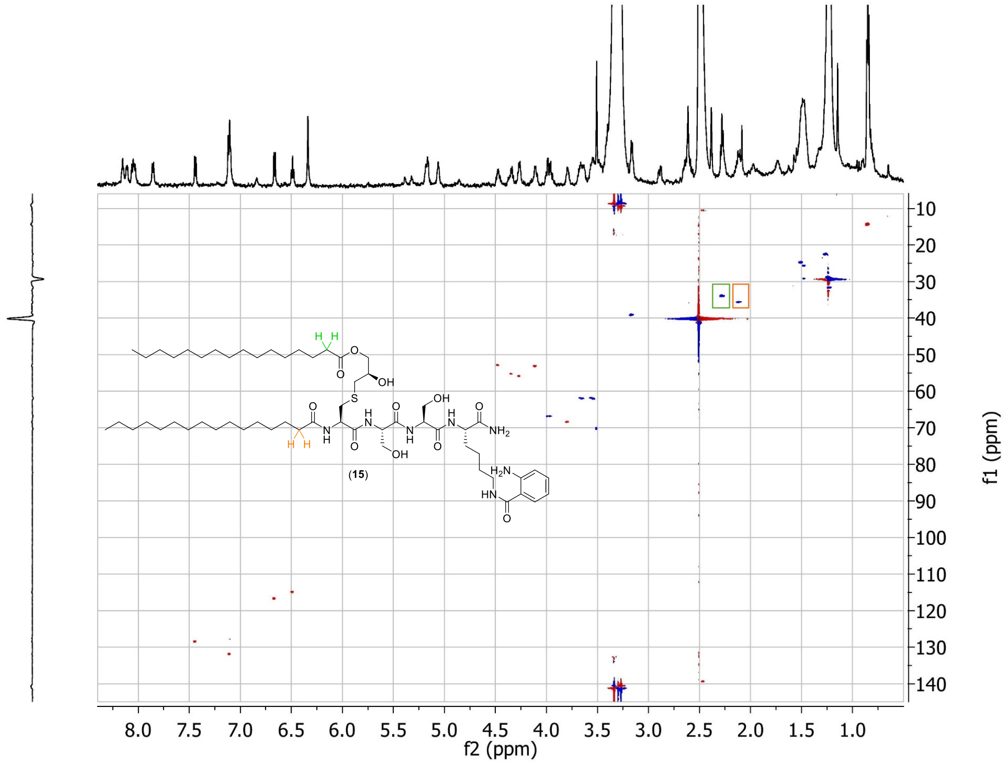

Supplementary Figure 29. ^{1}H-^{13}C HSQC of protiated product lyso-FP2. Following the acyl shift upon Lit reaction the α-CH_2 groups of the ester-linked (green) and amide-linked (orange) acyl chains become distinct, producing characteristic HSQC signals at (2.27, 33.5) ppm and (2.11, 35.5) ppm respectively.

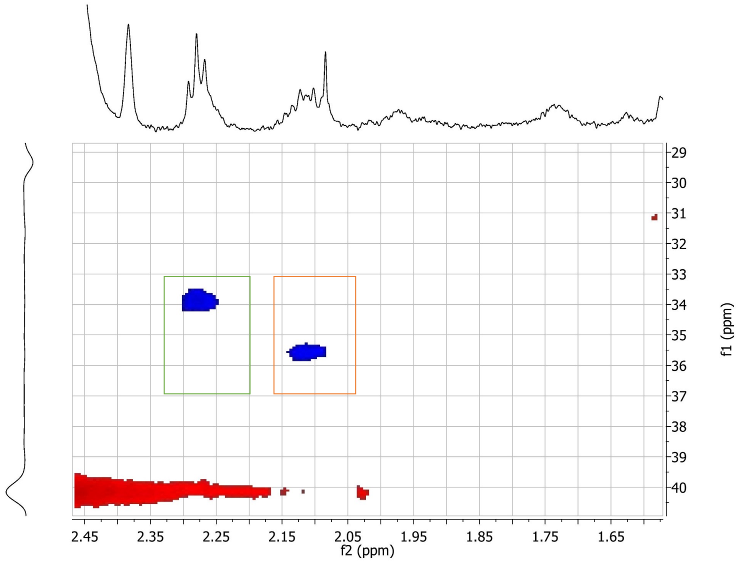

Supplementary Figure 30. Expanded view of Supplementary Fig. 29 highlighting the non-equivalent α-CH_2 signals in lyso-FP2.

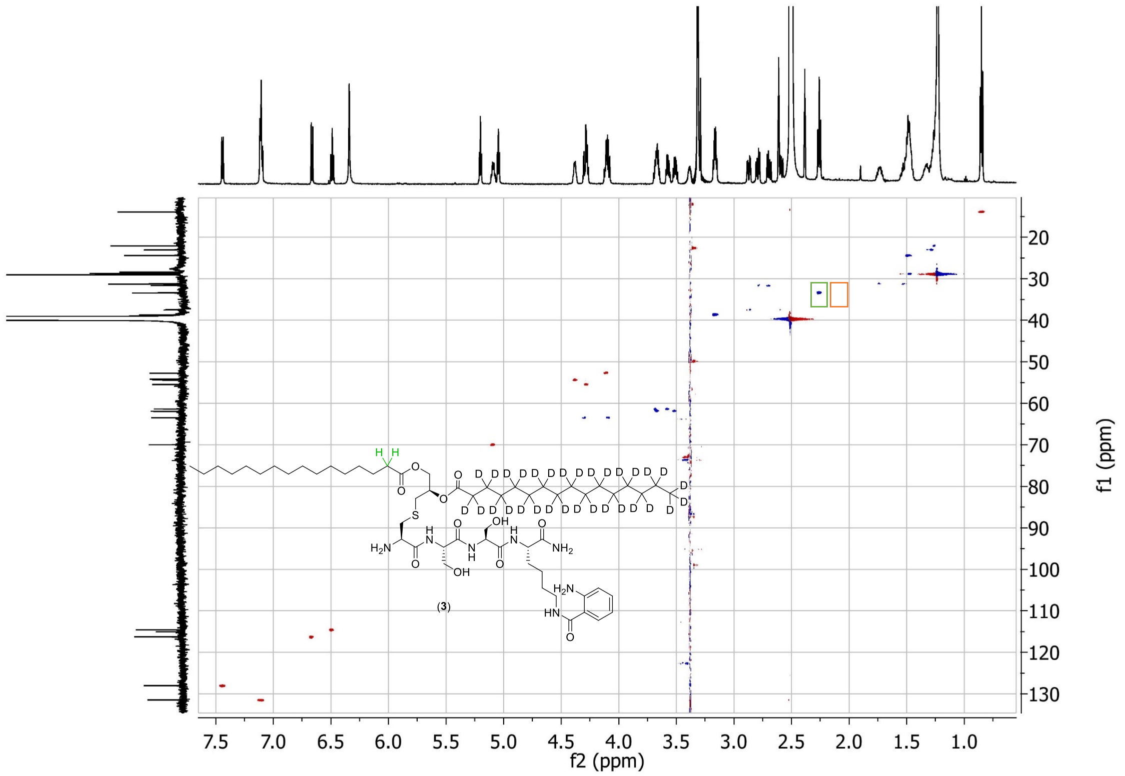

Supplementary Figure 31. ^{1}H-^{13}C HSQC of deuterated substrate dFP2. The two hydrogens (green) of the ester-linked α-CH_2 group appear as one signal highlighted in green.

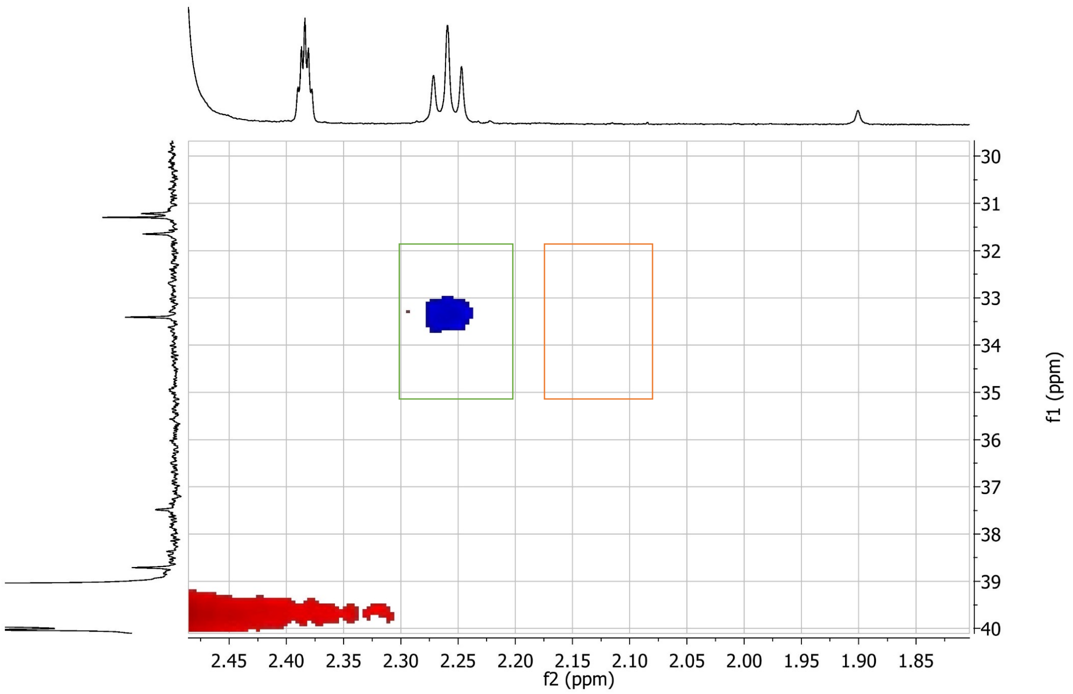

Supplementary Figure 32. Expanded view of Supplementary Fig. 31.

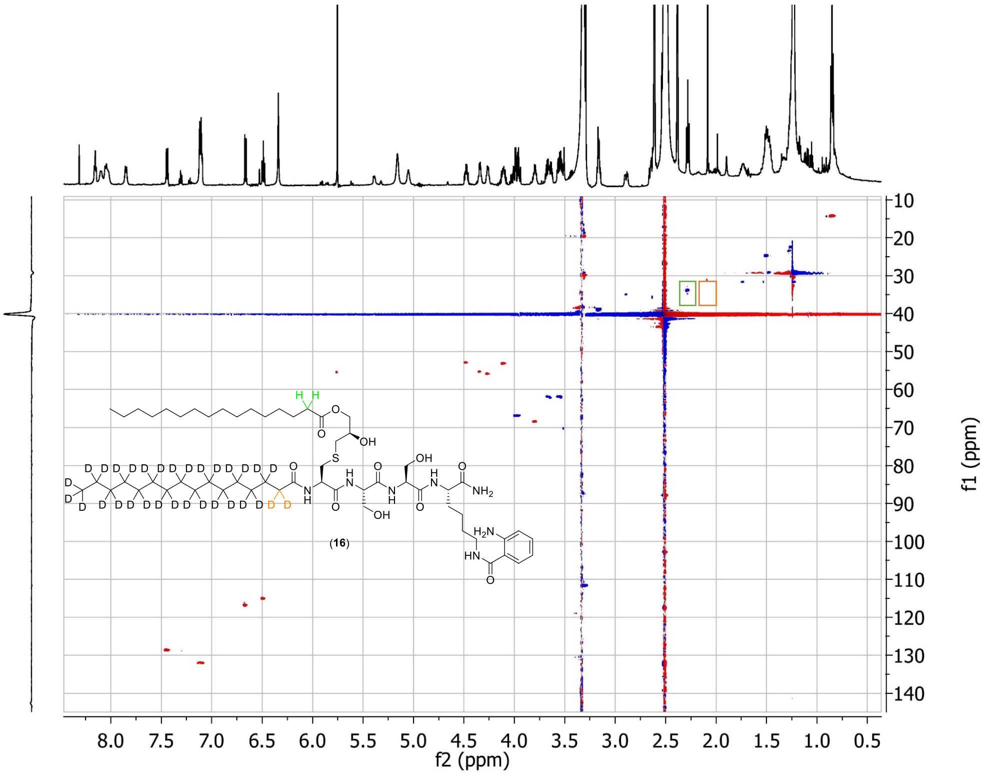

Supplementary Figure 33. ^{1}H-^{13}C HSQC of deuterated product lyso-dFP2. The signal arising from the two hydrogens (green) of the α-CH_2 group appear at the same chemical shift as in the substrate showing they remain ester linked (and not amide linked) in the product. This confirms that the *sn*-1 acyl chain in the product is protiated.

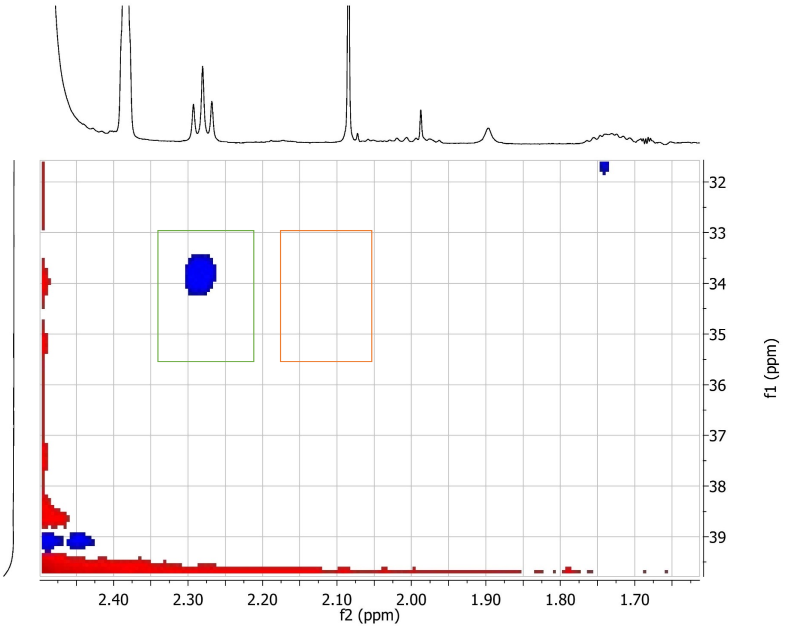

Supplementary Figure 34. Expanded view of Supplementary Fig. 33 highlighting the lack of signal at 2.12 ppm.

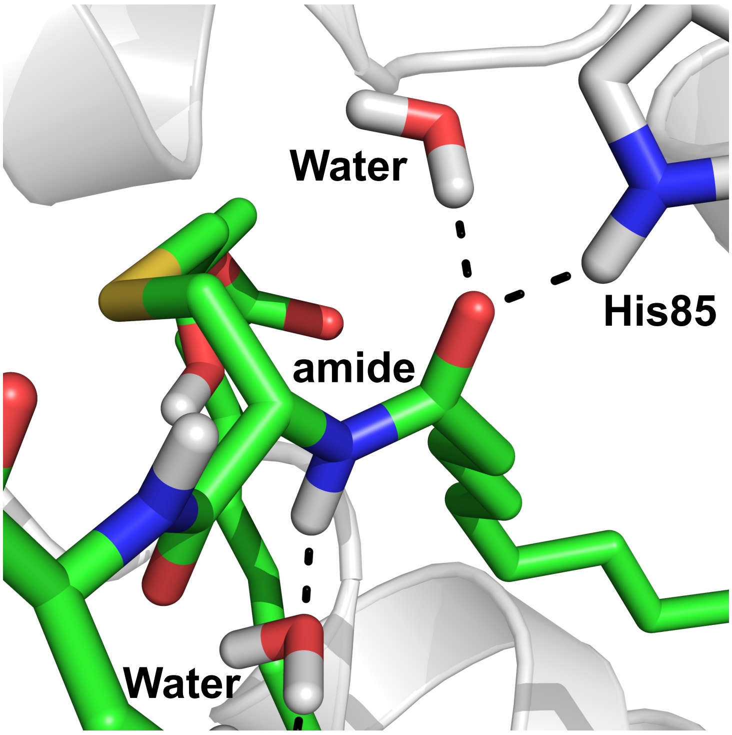

Supplementary Figure 35. The newly formed amide bond in the Lit product hydrogen bonds with water molecules as observed in MD simulations. The view is into the active site modelled to include the Lit product. The carbonyl oxygen and the amide hydrogen of the amide linkage each hydrogen bond with a single water molecule.

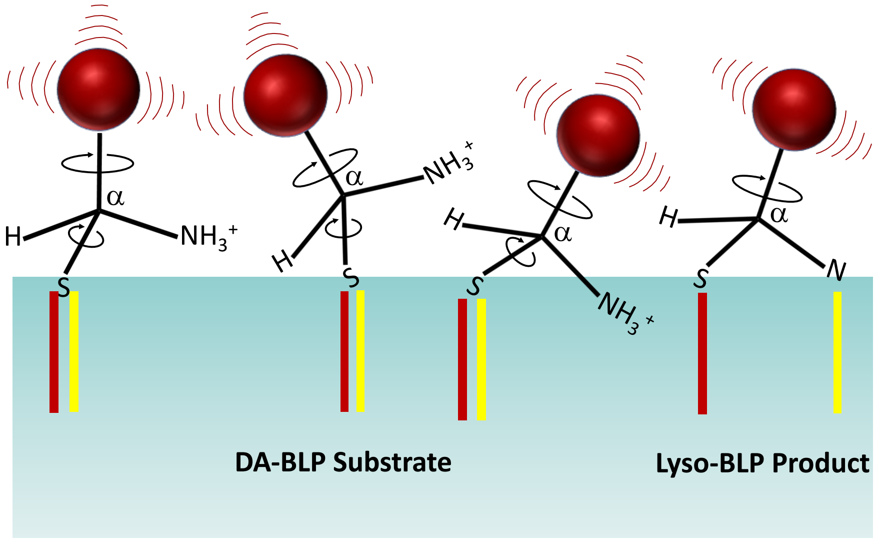

Supplementary Figure 36. Possible effects of acyl chain position in a diacylated BLP on mobility and disposition of the protein at the membrane interface. The BLP used in this example has an extramembranal ultra-domain (red sphere). The lyso form is doubly anchored by two acyl chains (red, yellow bars) from two arms of the tetrahedral α-carbon at the N-terminal cysteine. It has considerably fewer degrees of motional freedom than the DA-BLP form which is linked to the α-carbon by a single arm. Note too that the free amino group in the DA-BLP is protonated and cationic whilst that part of the lyso-BPL is uncharged at physiological pH.

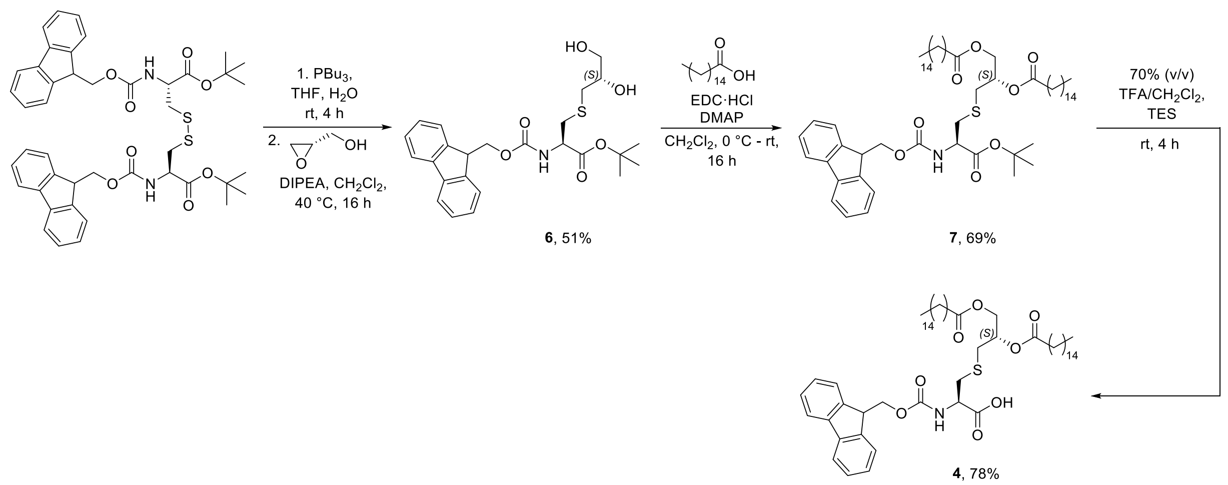

6, 51%

7, 69%

4, 78%

Supplementary Figure 37. Synthetic scheme of Fmoc-Cys((*S*)-2,3-bis(palmitoyloxy)propyl)-OH (4).

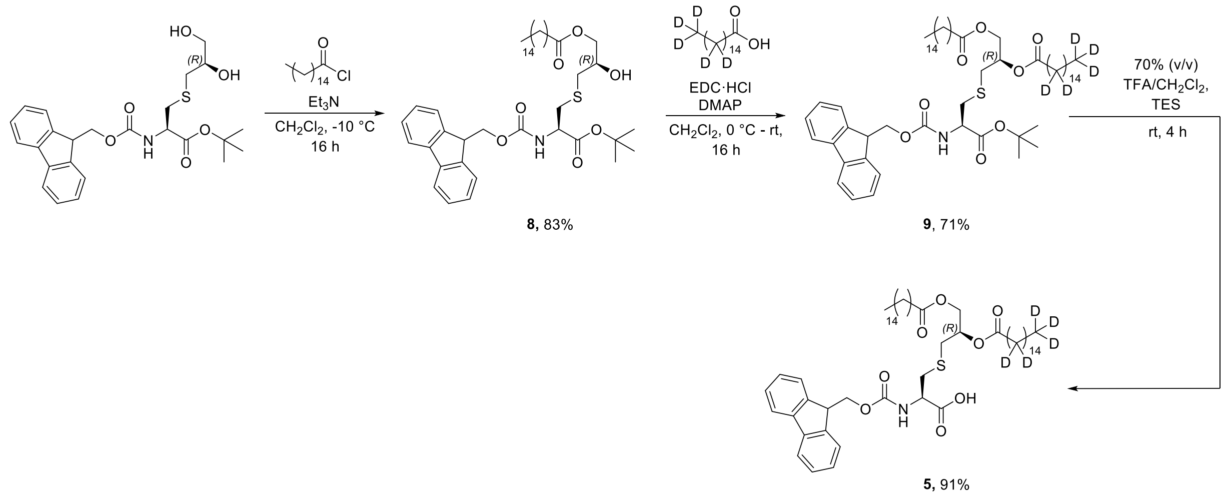

8, 83%

9, 71%

5, 91%

Supplementary Figure 38. Synthetic scheme of Fmoc-Cys((*R*)-2-((hexadecanoyl-d_{31})oxy)-3-(palmitoyloxy)propyl)-OH (5).

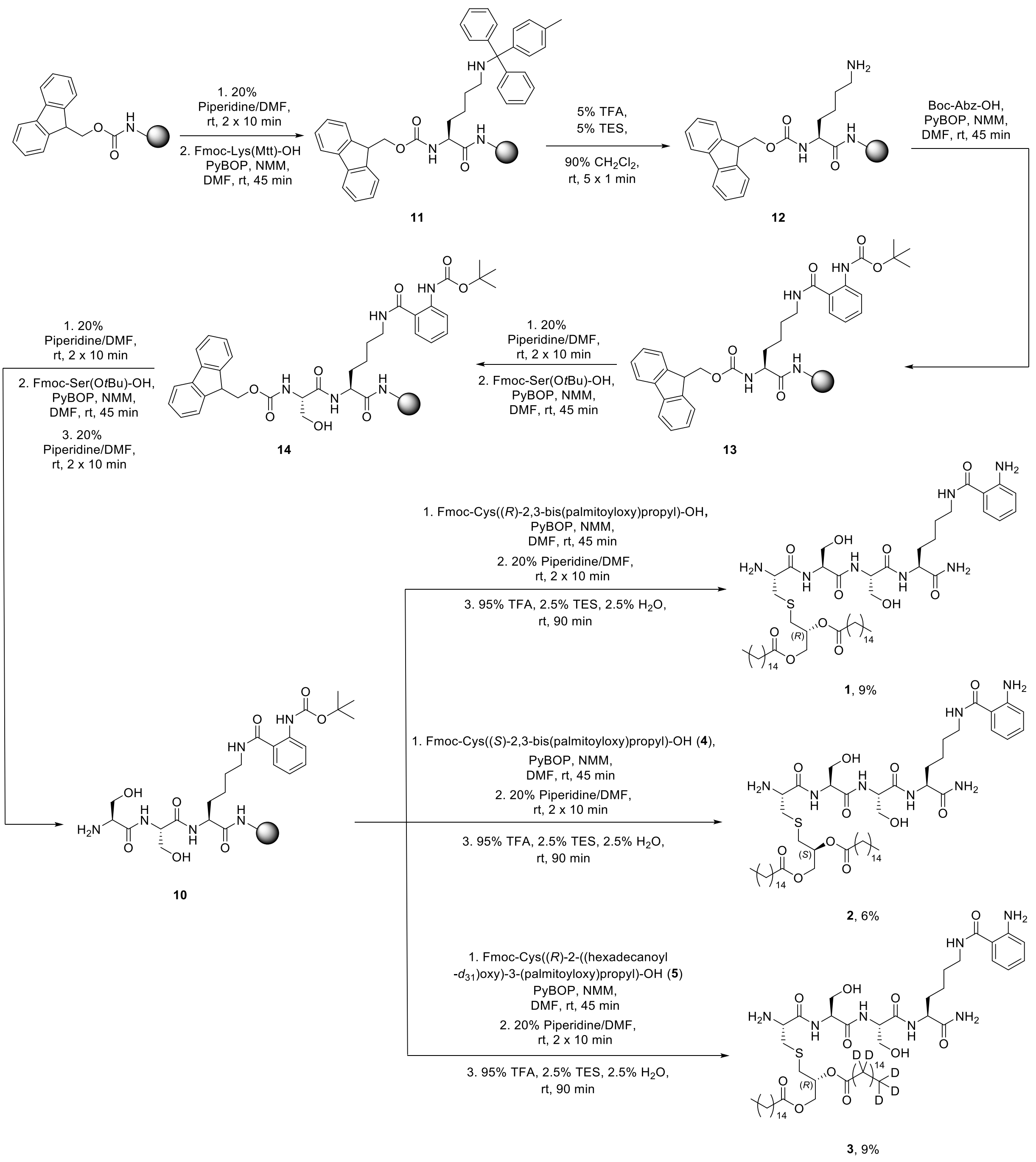

Supplementary Figure 39. Synthetic scheme of peptide probes 1-3.

NMR Spectra of Novel Compounds

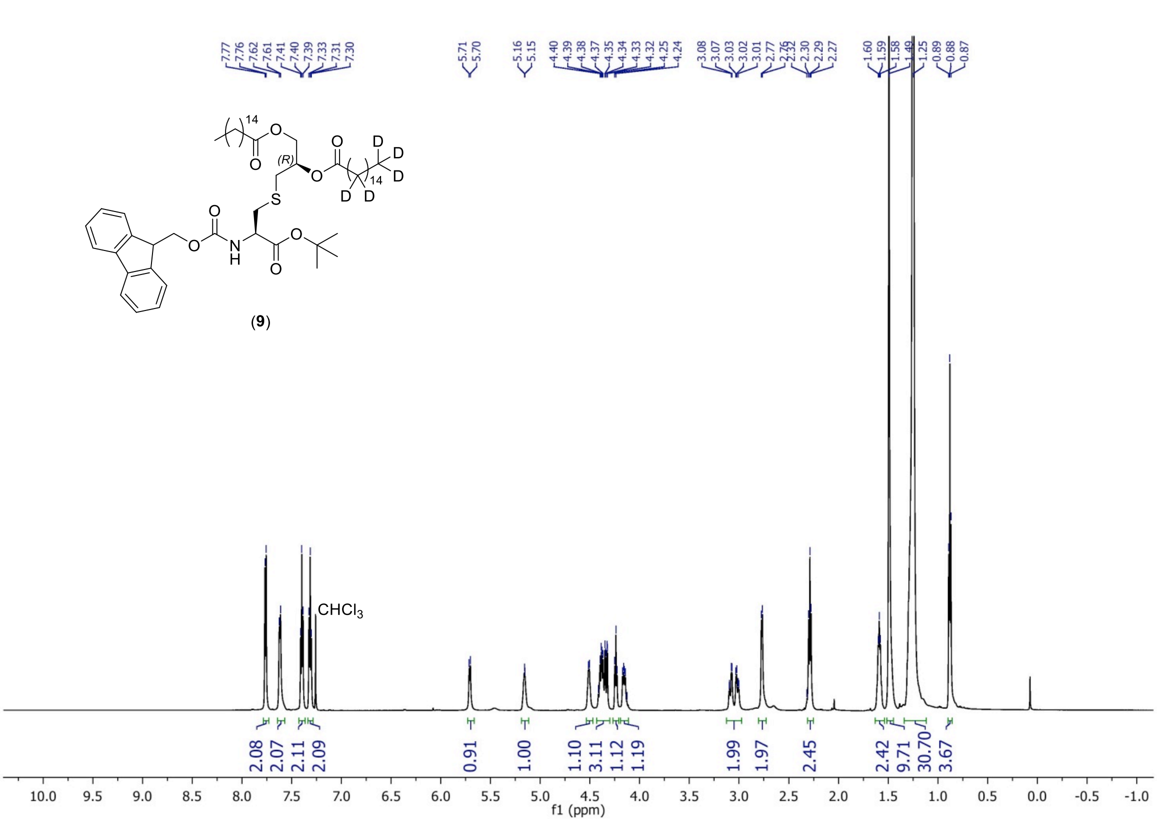

Supplementary Figure 40. **^{1}H NMR (600 MHz, $CDCl_3$) of compound 9.**

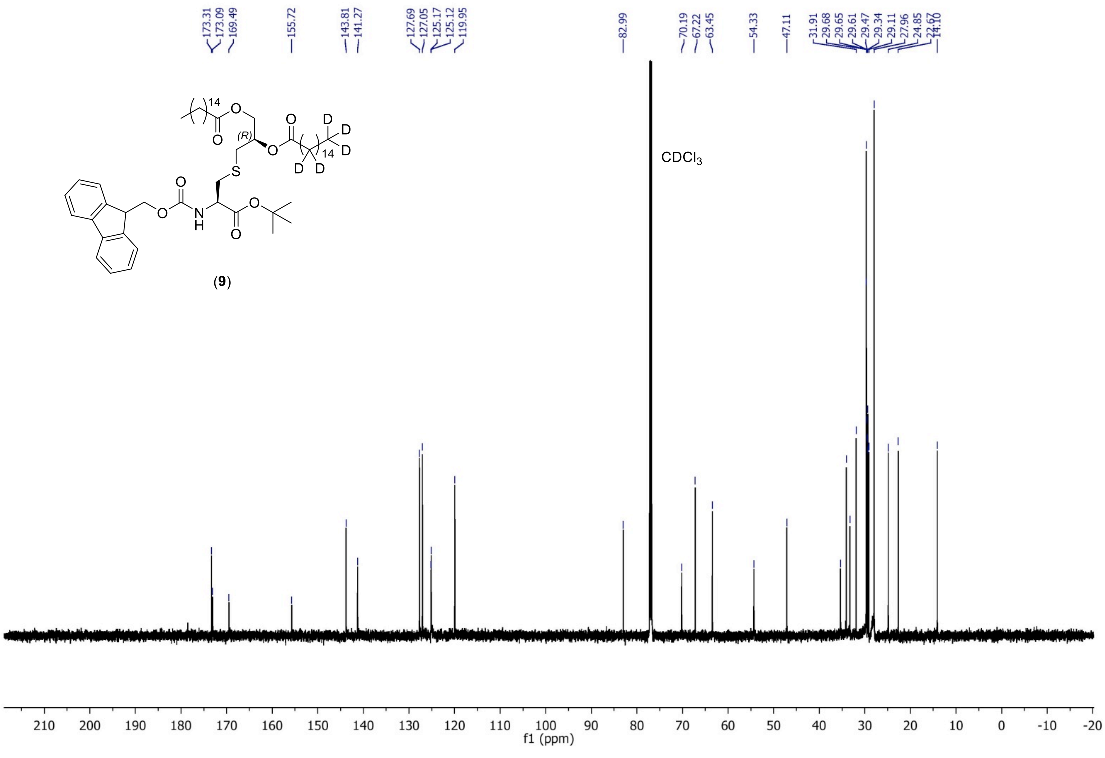

Supplementary Figure 41. **^{13}C NMR (151 MHz, $CDCl_3$) of compound 9.**

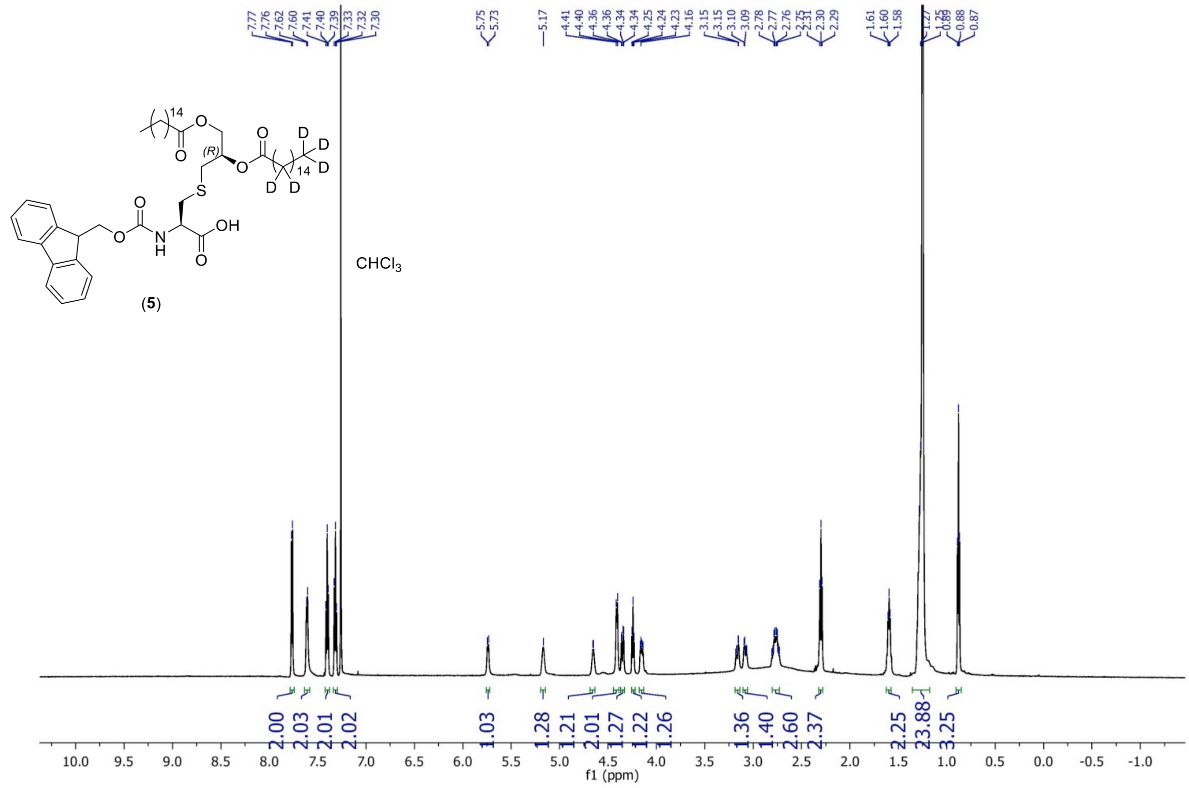

Supplementary Figure 42. ^{1}H NMR (600 MHz, $CDCl_3$) of compound 5.

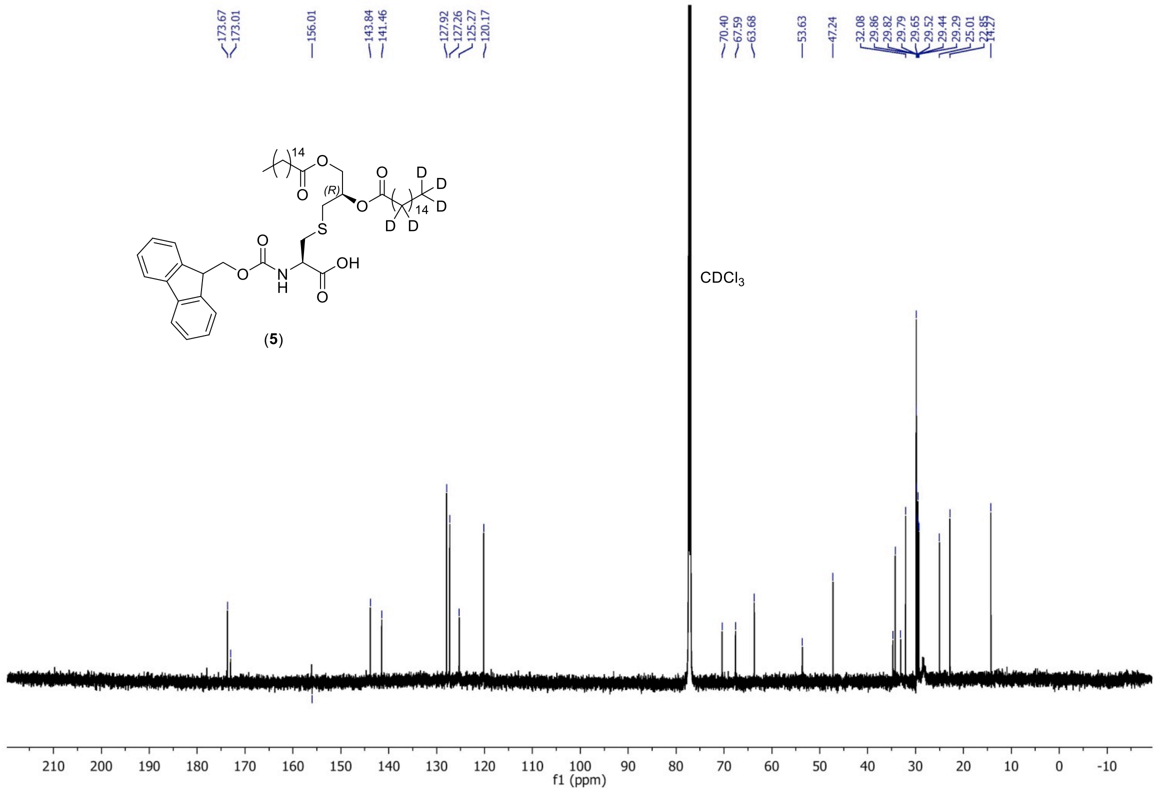

Supplementary Figure 43. ^{13}C NMR (151 MHz, $CDCl_3$) of compound 5.

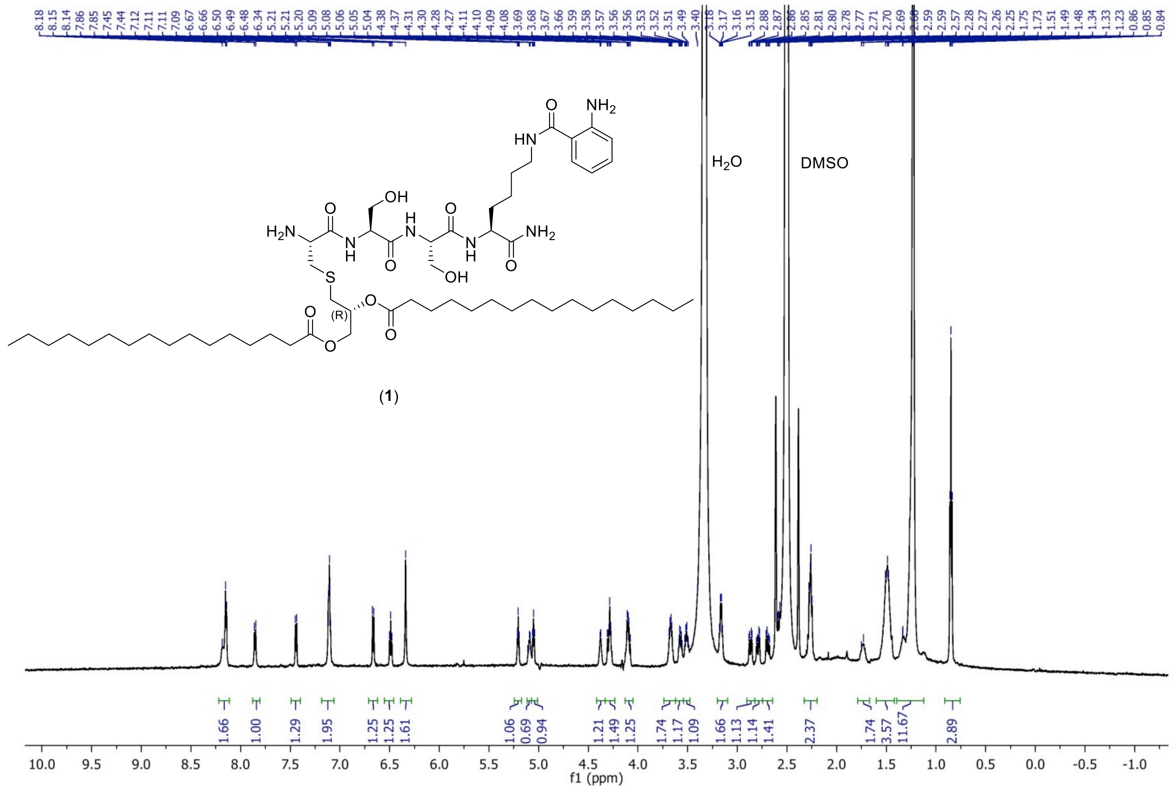

Supplementary Figure 44. ^{1}H NMR (600 MHz, DMSO-d_6) of compound 1.

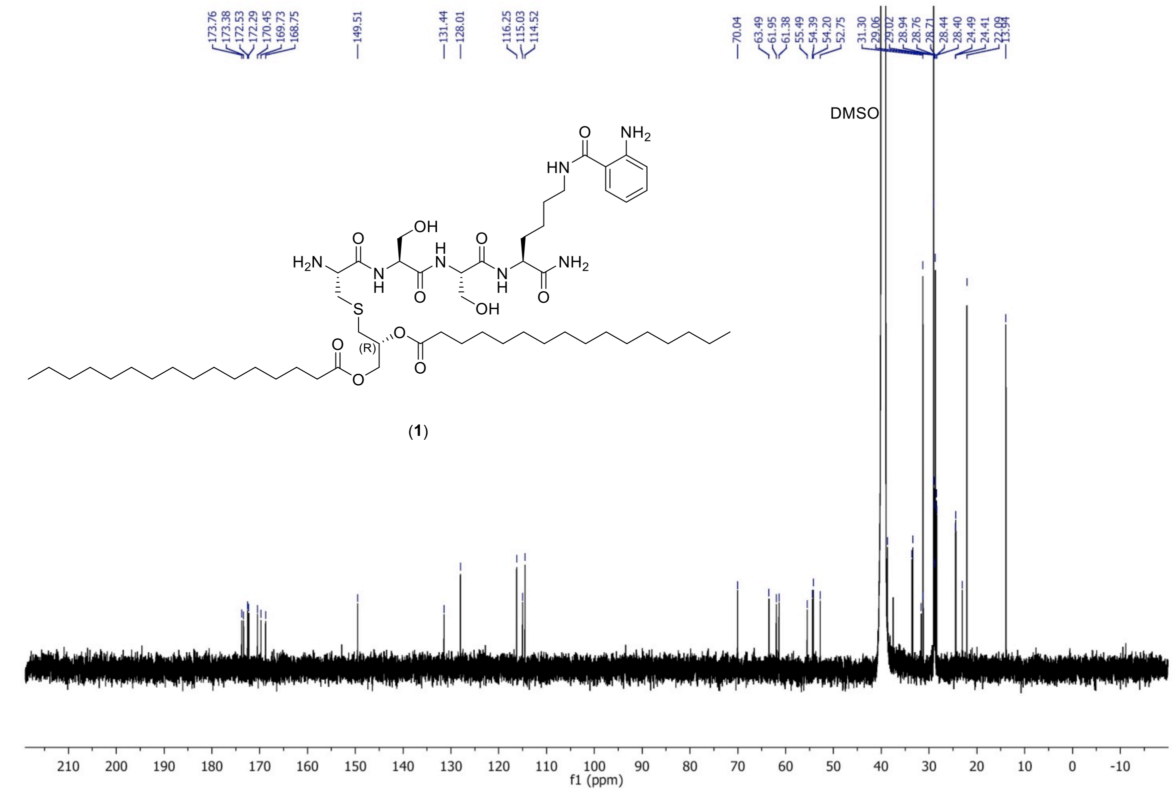

Supplementary Figure 45. ^{13}C NMR (151 MHz, DMSO-d_6) of compound 1.

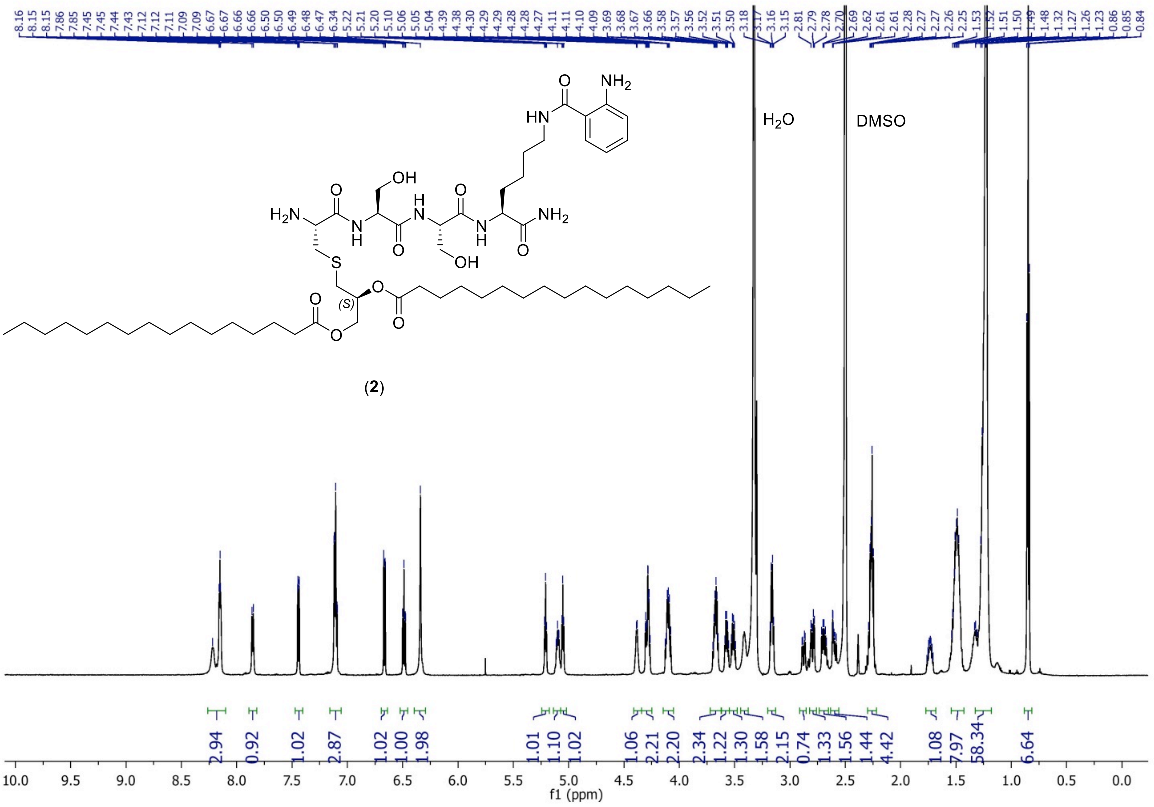

Supplementary Figure 46. ^{1}H NMR (600 MHz, DMSO-d_6) of compound 2.

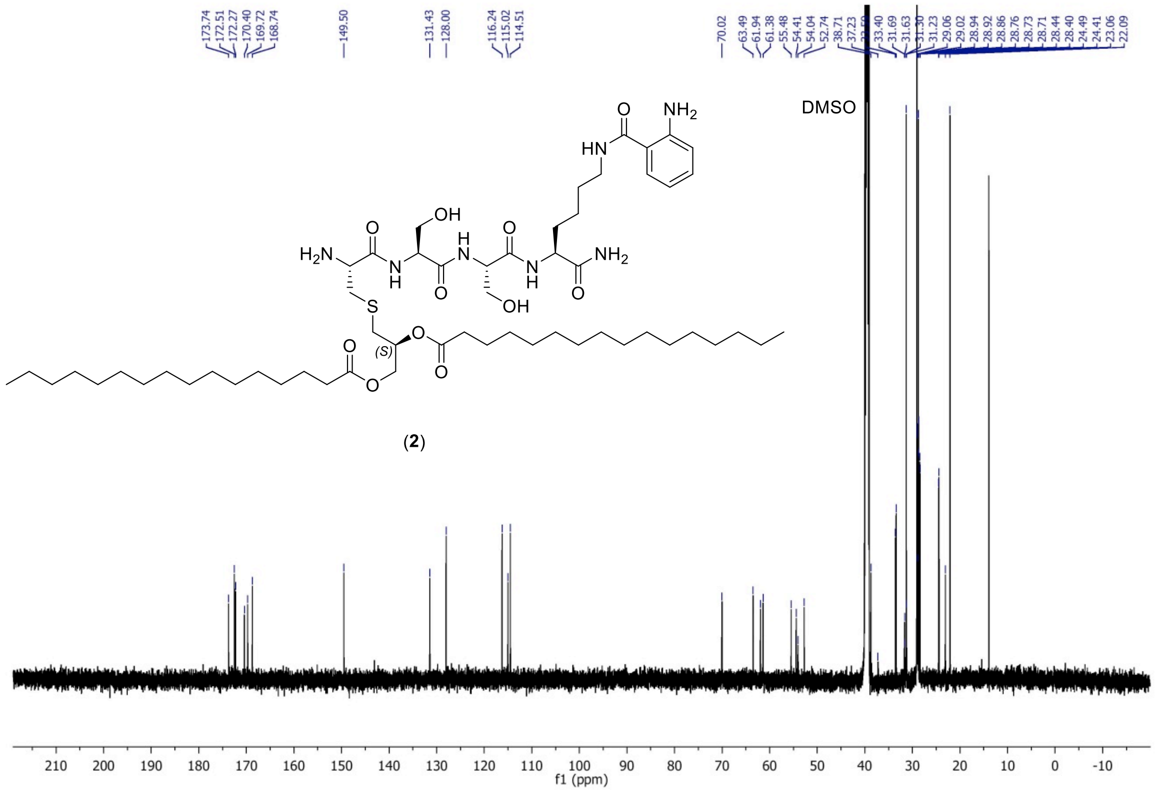

Supplementary Figure 47. ^{13}C NMR (151 MHz, DMSO-d_6) of compound 2.

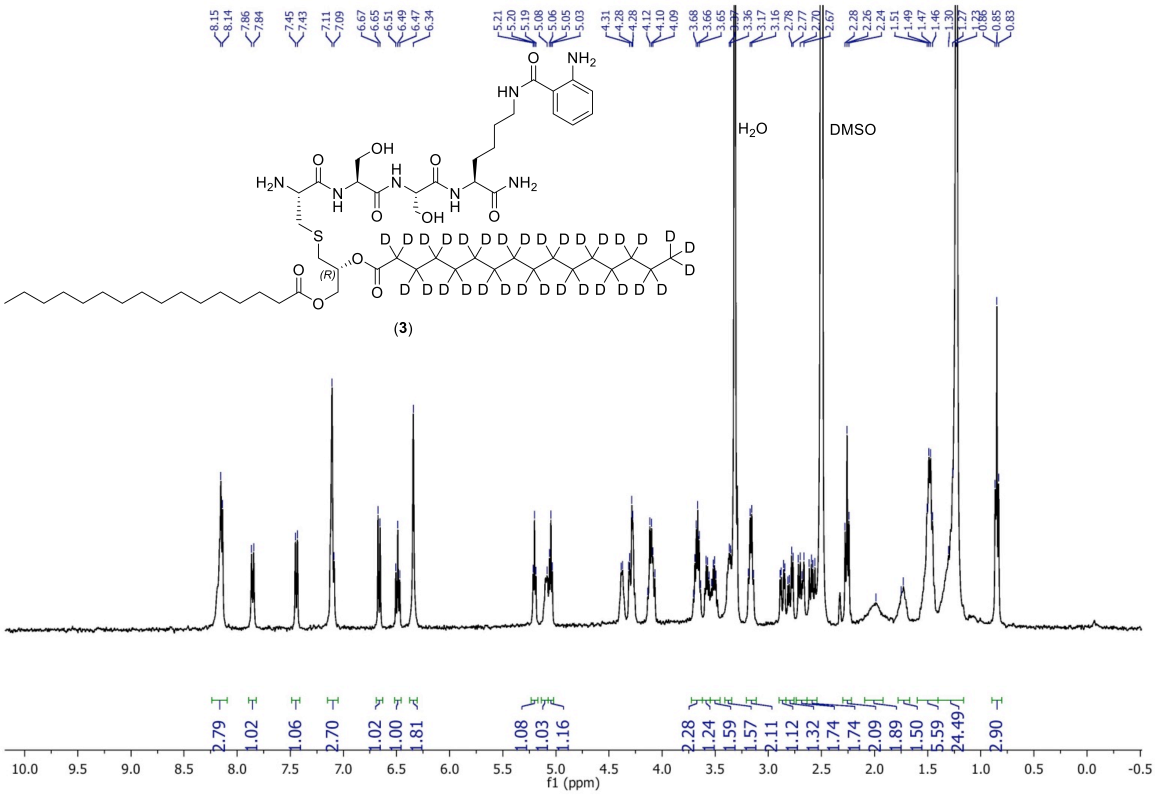

Supplementary Figure 48. ^{1}H NMR (600 MHz, DMSO-d_6) of compound 3.

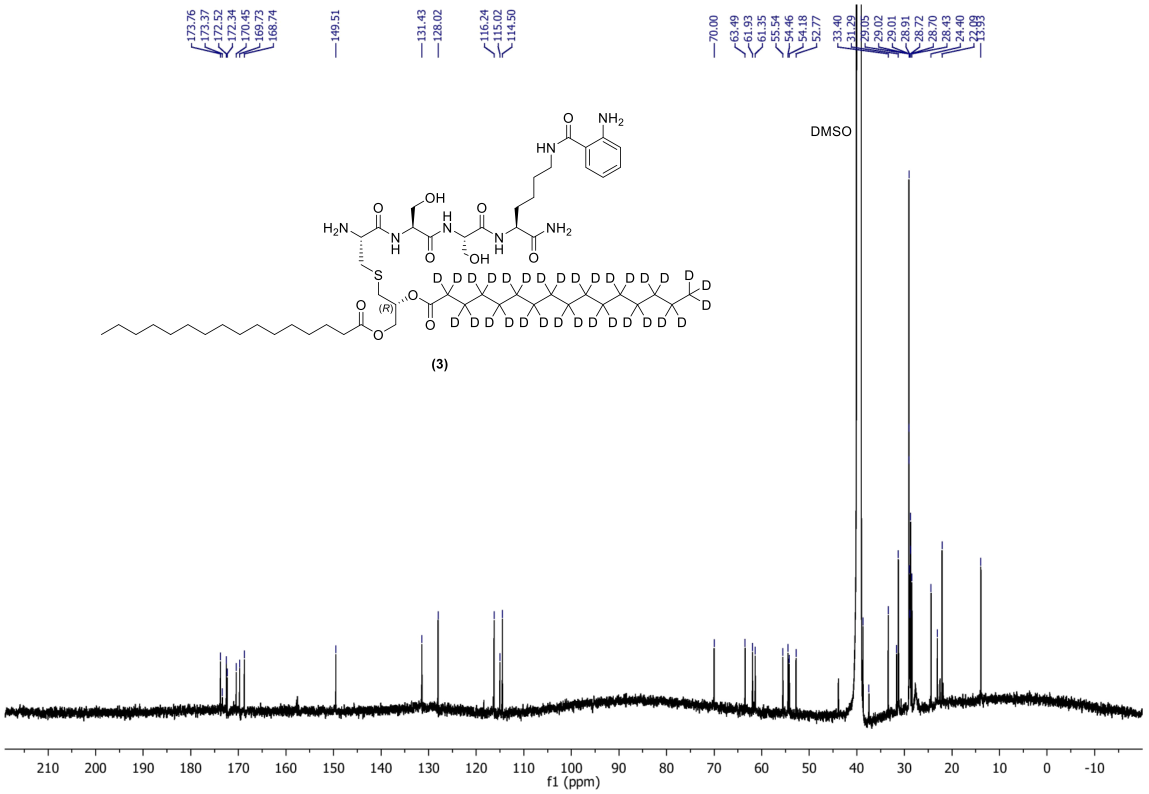

Supplementary Figure 49. ^{13}C NMR (151 MHz, DMSO-d_6) of compound 3.

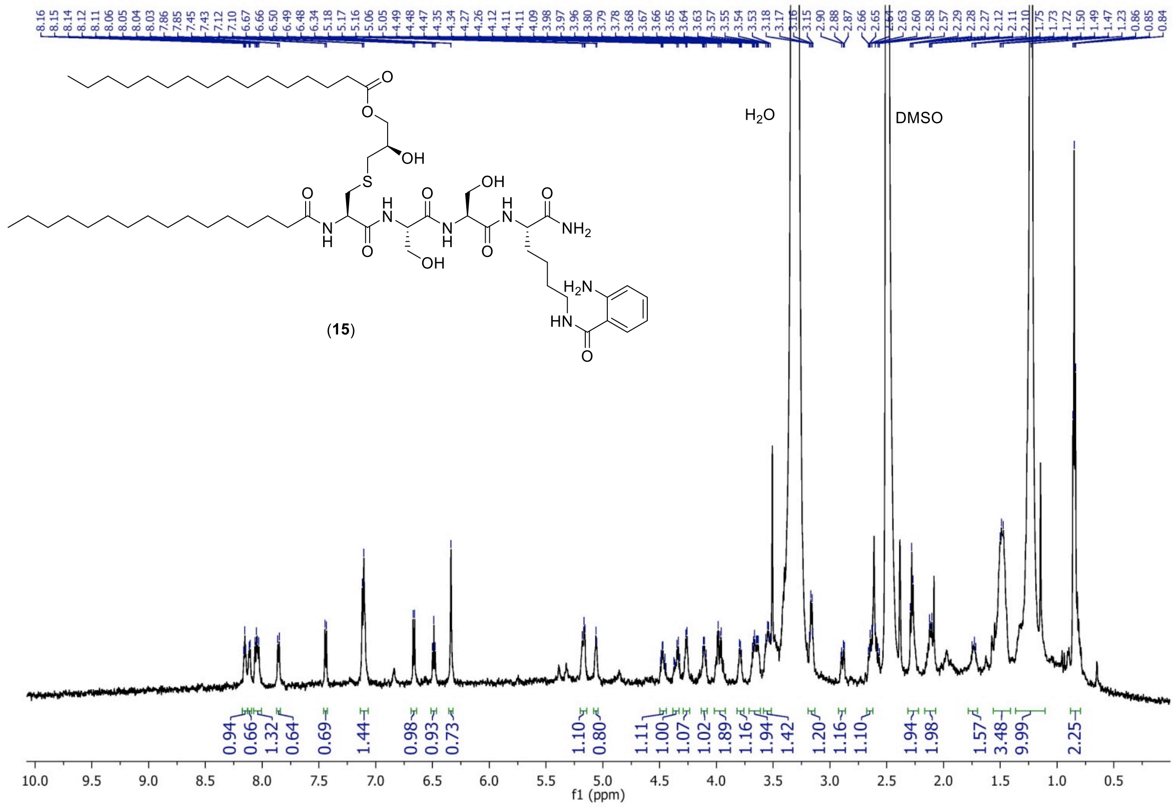

Supplementary Figure 50. ^{1}H NMR (600 MHz, DMSO-d_6) of compound 15.

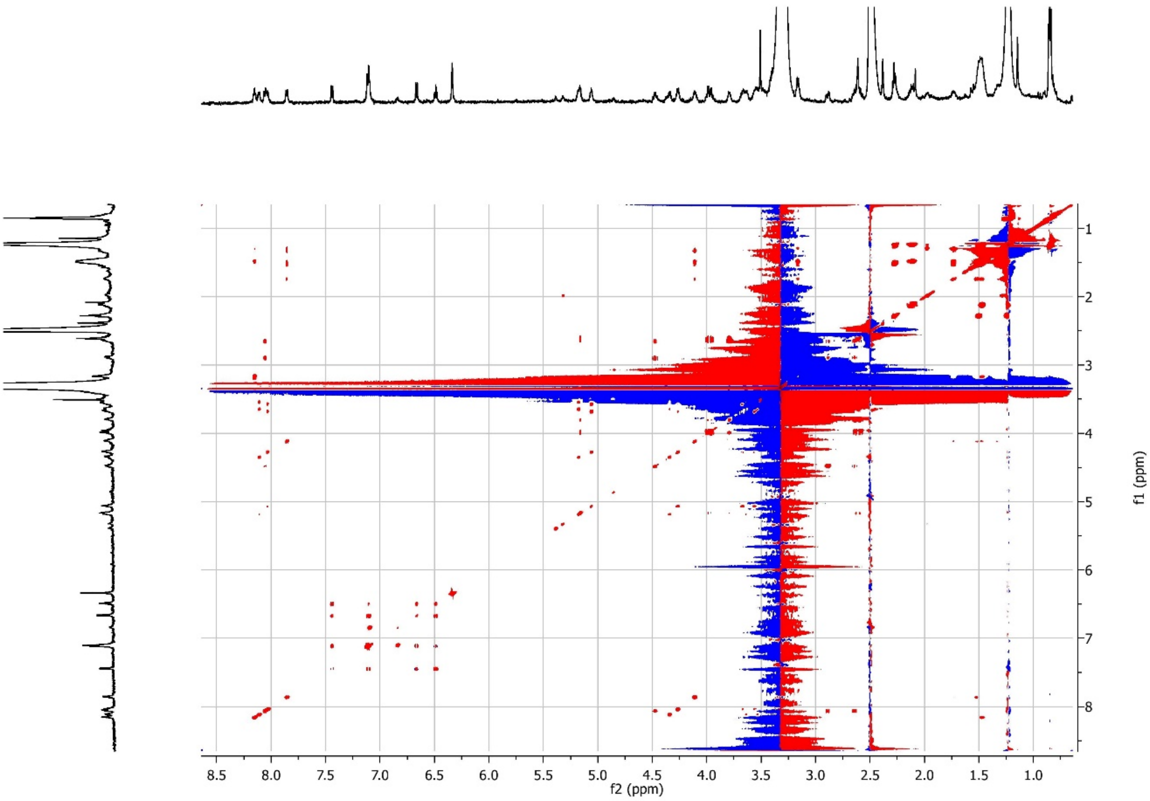

Supplementary Figure 51. TOSCY (600 MHz, DMSO-d_6) of compound 15.

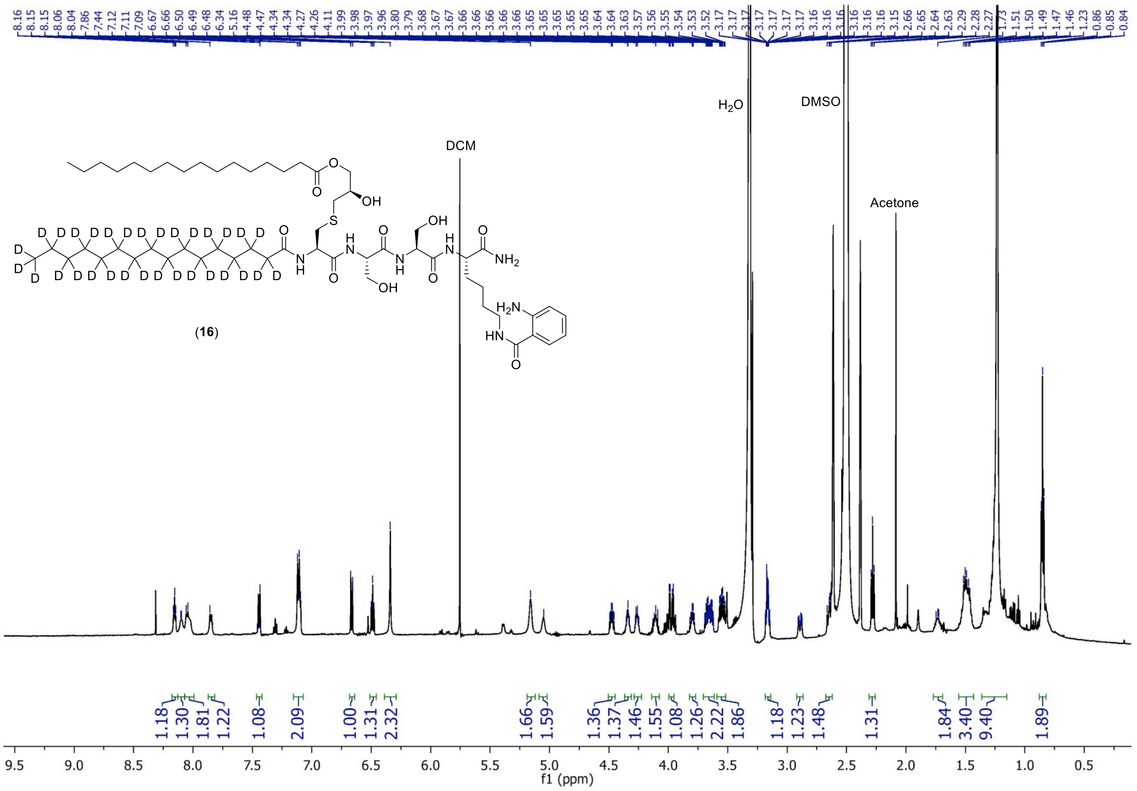

Supplementary Figure 52. ^{1}H NMR (600 MHz, DMSO-d_6) of compound 16.

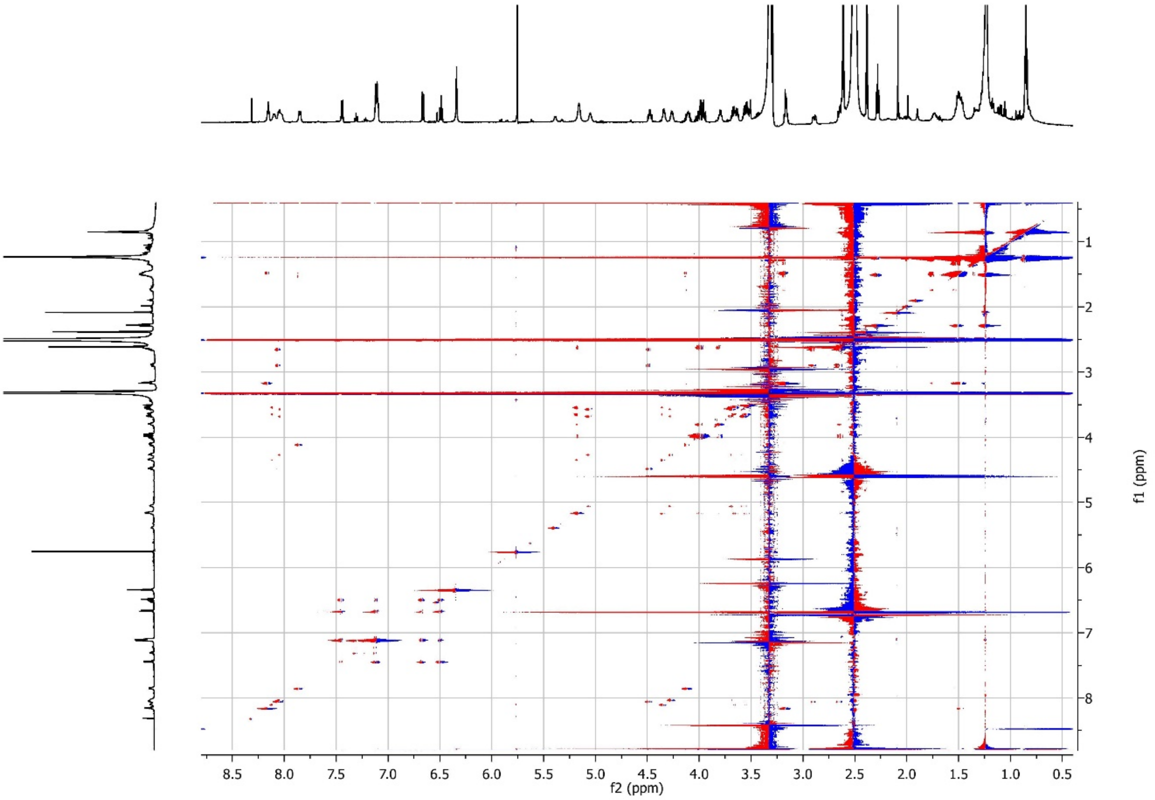

Supplementary Figure 53. TOSCY (600 MHz, DMSO-d_6) of compound 16.

Supplementary Tables 1-6

Supplementary Table 1. Data collection and refinement statistics.[a]

	Lit-Se	Lit_1	Lit_2	LitH85A	LitH85R
PDB ID		7B0O	7B0P	7B0Q	7B0R
**Data collection**					
Space group	$P2_1$	$P2_1$	$P2_12_12$	$C2_1$	$C2_1$
Cell dimensions					
a, *b*, *c* (Å)	66.50, 43.37, 117.50	67.72, 44.70, 116.87	51.17, 192.24, 76.80	115.24, 45.14, 70.17	114.47, 44.87, 69.54
α, β, γ (°)	90, 92.92, 90	90, 92.38, 90	90, 90, 90	90, 121.38, 90	90, 121.06, 90
Beamline	SLS-X06SA-PXI	DLS-I24	DLS-I24	DLS-I24	DLS-I24
Wavelength (Å)	0.97882	0.96862	0.96860	0.96863	0.96862
Resolution (Å)	58.74 - 3.20 (3.30 - 3.20)[b]	67.76 - 2.27 (2.45 - 2.27)	60.00 - 1.95 (2.28 - 1.95)	49.19 - 2.43 (2.64 - 2.43)	40.8 - 2.20 (2.33 - 2.20)
No. of reflections (total/unique)	1209886 (23132)	148041 (24031)	155441 (29001)	55527 (8396)	94116 (14531)
R_{meas}	0.44 (3.80)	0.32 (1.57)	0.75 (7.41)	0.12 (1.74)	0.15 (1.42)
$I/\sigma(I)$	10.79 (1.42)	3.40 (1.40)	4.40 (1.70)	9.70 (1.30)	10.46 (1.23)
$CC_{1/2}$ (%)	99.00 (61.00)	95.50 (17.90)	72.80 (16.20)	99.80 (51.80)	99.80 (64.40)
Completeness (%)	100 (100)	91.50 (57.60)[c]	89.50 (47.40)[c]	92 (66.60)[c]	92.6 (67.10)
Multiplicity	52.30 (20.88)	6.20 (6.90)	5.40 (3.90)	6.60 (6.80)	6.48 (5.71)
**Phasing**	Se-SAD				
Heavy atom sites	14				
CCanom (%)	36				
**Refinement**					
Resolution (Å)		43.32 – 2.33	60.00 – 1.94	49.19 – 2.42	40.80 – 2.20
**Reflections**		19605	27837	8388	14504
R_{work}/R_{free}		0.25/0.29	0.23/0.25	0.22/0.24	0.23/0.28
R.m.s. deviations					
Bond lengths (Å)		0.003	0.004	0.004	0.002
Bond angles (°)		0.69	0.72	0.82	0.51
No. atoms					
Protein		3494	3606	1788	1818
Ligand/ion[d]		374	400	229	161
Water		43	168	21	43
B-factor					
Proteins		52.82	31.90	62.58	54.27
Ligand/ions		69.11	56.85	80.09	67.32
Water		42.65	32.70	51.55	47.88
Ramachandran Plot					
Favored (%)		98.35	99.08	98.62	99.55
Allowed (%)		1.65	0.92	1.38	0.45
Outliers (%)		0	0	0	0
MolProbity Clashscore		11.67	8.74	10.06	7.61

[a] Data processing statistics are reported with Friedel pairs separated for Lit-Se and Friedel pairs merged for Lit_1, Lit_2, LitH85A and LitH85R

[b] Values in parentheses are for the highest resolution shell

[c] Ellipsoidal completeness, as defined by *autoPROC/STARANISO*, is referred to here

[d] Other 'ligands' include citrate, monoolein, glycerol, and PEG

Supplementary Table 2. Conserved residues in Lit from *B. cereus*.

Residue[a]	Position	Conservation (%)	Alternative residues[b]
F	157	99.74	**L** (0.26)
H	153	99.23	**R** (0.39), **K** (0.26), **F** (0.13)
H	85	97.83	**R** (0.38), **Q** (0.38), **A** (0.26), **N** (0.26), **V** (0.26), **K** (0.13), **I** (0.51)
W	162	97.58	**V** (0.76), **I** (0.51), **S** (0.51), **Y** (0.25), **A** (0.13), **E** (0.13), **F** (0.13)
V	89	95.03	**C** (2.42), **L** (0.89), **A** (0.64), **T** (0.51), **I** (0.38), **F** (0.13)
F	149	92.37	**W** (5.60), **L** (1.02), **Y** (0.51), **A** (0.13), **I** (0.13), **S** (0.13), **V** (0.13)
F	152	88.92	**L** (6.31), **M** (2.19), **V** (1.68), **A** (0.64), **I** (0.26)
I	172	88.41	**S** (3.69), **T** (3.31), **A** (0.51), **C** (0.13), **F** (0.26), **K** (0.13), **L** (1.66), **M** (0.13), **P** (0.26), **R** (0.13), **V** (0.76), **W** (0.38), **Y** (0.26)
P	176	86.62	**K** (5.10), **T** (3.69), **Q** (1.27), **G** (1.02), **N** (0.38), **H** (0.38), **D** (0.26), **E** (0.26), L (0.38), **S** (0.13), **V** (0.13), **Y** (0.15), **A** (0.13), **F** (0.13)
D	169	84.10	**S** (12.72), E (1.78), **H** (0.51), **A** (0.25), **T** (0.25), **C** (0.13), **G** (0.13), **P** (0.13)

[a] Conserved residues were identified from a Clustal Omega[8] sequence alignment of 788 Lit orthologues from the UniProt Reference Proteomes database[9]. Conservation scores were calculated using the Consurf server[10,11]. Residues are identified by the one-letter amino acid code.

[b] Alternative residues are indicated by residue type with % conservation in parenthesis.

Supplementary Table 3. His85 and His153 interactions with local aromatic residues along the catalytic reaction pathway from MD trajectories.[a]

Residue	Residue-His85 Interaction Energy, kcal/mol				
	Empty	Substrate	IM1	IM2	Product
**Phe86**	**-22.1±3**	**-20.6±1**	**-18.7±2**	**-20.6±1**	**-19.1±1**
Phe149	0.1±0.2	0.1±0.1	0	0.4±0.4	0.2±0.3
Phe152	0.2±0.2	0.2±0.3	0.1±0.3	0.3±0.3	0
His153	-3.8±2	-3.1±1	1.3±0.1	1.5±0.2	1.4±2
**Phe157**	**-5.4±1**	**-6.2±1**	**-5.8±2**	**-6.2±1**	**-5.9±2**
**Trp162**	-1.6±3	**-8.0±5**	**-10.0±4**	-0.4±2	0.2±2
Phe164	-0.2±2	0.7±0	0.5±1	-0.2±1	0.4±1
Phe178	0	0	0	0	0
Phe180	0	0.3±2	0.1±0.2	0	0.1±0.2

Residue	Residue-His153 Interaction Energy, kcal/mol				
	Apo	Substrate	IM1	IM2	Product
His85	-3.8±3	-3.1±1	1.3±2	1.5±1	1.4±1
Phe86	0	0	0	0	0
**Phe149**	**-12.9±3**	**-12.9±2**	**-15.1±4**	**-13.8±3**	**-12.5±4**
**Phe152**	**-15.5±2**	**-15.3±1**	**-6.9±2**	**-6.9±1**	**-7.0±2**
**Phe157**	**-4.1±1**	**-4.9±2**	**-4.1±2**	**-5.3±5**	**-3.7±3**
**Trp162**	**-5.3±2**	**-2.6±0.3**	-0.4±3	**-5.9±5**	**-9.3±4**
Phe164	-0.4±1	-0.2±0	0	0	0.1±2
Phe178	0	0	0	0	0
Phe180	-0.4±1	0	-0.2±1	0	0.2±0.5

[a] The calculated interaction energy represents a total average energy including electrostatic and Van der Waals energies. His85 forms strong electrostatic interactions with Phe86 and Phe157 in empty Lit and during the catalytic reaction, and with Trp162 in the substrate-bound complex and IM1. His153 engages with Phe149, Phe152, Phe157 and Trp162 in empty Lit and during the catalytic reaction (apart from IM1 for Trp162). Residues forming strong interactions are highlighted in bold. The starting coordinates of IM1 and IM2 complexes are derived from QM/MM calculations.

Supplementary Table 4. Lipopeptide-Lit residue interaction energies obtained from MD simulation trajectories of the Lit structure bound to substrate, product and monoacylated lipopeptide, and in intermediate states IM1 and IM2.[a]

Residue	Lipopeptide-Residue Interaction Energy, kcal/mol				
	Substrate	IM1	IM2	Product	Monoacylated Lipopeptide
**His85**	**-19.6±5**	**-14.9±9**	**-15.9±3**	**-22.0±5**	4.1±2
**His153**	**-14.3±7**	-1.7±4	-0.3±2	-0.3±3	-8.0±6
Phe86	-1.5±1	-1.9±1	-3.3±1	-3.8±2	-4.0±4
**Phe149**	**-5.6±3**	**-3.3±2**	**-2.4±1**	**-1.8±2**	**-3.3±1**
**Phe152**	**-5.0±1**	**-6.7±2**	**-5.9±2**	**-4.9±2**	**-5.0±1**
Phe157	-0.5±1	-1.0±1	-1.0±1	-0.9±1	-1.0±1
**Trp162**	**-3.4±5**	**-2.6±1**	**-4.1±1**	**-7.9±2**	**-8.2±3**
**Phe164**	**-3.1±1**	**-3.3±3**	**-3.5±1**	**-4.1±1**	**-6.0±2**
Phe178	-0.9±0.4	-1.2±1	-1.0±1	-1.7±1	1.2±1
**Phe180**	**-6.1±1**	**-7.3±2**	**-10.2±2**	**-9.1±2**	**-5.4±1**

[a] The calculated interaction energy represents a total average energy including electrostatic and Van der Waals energies. The data reveal strong electrostatic interactions between the lipopeptide and His85 throughout the catalytic reaction, and with His153 in the substrate-bound complex. Strong Van der Waals interactions are observed with Phe149, Phe152, Trp162, Phe164 and Phe180 in the catalytic reaction. The monoacylated lipopeptide has weak interactions in the active site.

Supplementary Table 5. Inter-atomic distances observed in the QM/MM study of the Lit intramolecular transacylation reaction.

States	Distance (Å) *			
	His153(**N**ε) NH_3^+(**H**)	NH_3^+(**N**) C=O(**C**) (*sn*-2)	C=O(**C**)(**O**) (*sn*-2)	His85(**H**ε) C=O(**C**) (*sn*-2)
R	1.58	4.13	1.23	1.98
TS1	1.35	4.07	1.23	1.98
IM1	1.03	2.78	1.23	1.85
TS2	1.03	1.92	1.27	1.59
IM2	1.03	1.69	1.30	1.50
TS3	1.03	1.50	1.24	1.74
P	1.03	1.33	1.25	1.68

* Distance refers to the separation between atoms highlighted in red and bold

Supplementary Table 6. Primers used in this study.

Name	Sequence[a]
LitBce_NdeI_F[b]	TGGC***CATATG***GACCGTCTGATCACCCTGG
LitBce_XhoI_R	CAGA***CTCGAG***TTACGCGCTGAATTTCTTTTGGCTCATAC
Bce_W30P_F[c]	CCGCTGTACTATTTCGAGATCGATTTTCTG
Bce_W30P_R	CTTGCCATAAACCACCGCCATGG
Bce_L59N_F	AACATTACCTACCTGAGCCCGTTCTATG
Bce_L59N_R	CACATCGTAGTTACGTTTGATTTCGTC
Bce_H85A_F	CGGCCGTATTGCCTTTGTGGACG
Bce_H85E_F	CGGCCGTATTGAATTTGTGGACG
Bce_H85A/E_R	TTGGTGCTCATATCCAGG
Bce_H85D_F	CGGCCGTATTGACTTTGTGGACG
Bce_H85N_F	CGGCCGTATTAACTTTGTGGACG
Bce_H85D/N_R	TTGGTGCTCATATCCAGGG
Bce_H85Q_F	CAGTTTGTGGACGTTAAGAACATCCTGGTG
Bce_H85Q_R	AATACGGCCGTTGGTGCTCATATCC
Bce_H85R_F	CGGCCGTATTCGCTTTGTGGACG
Bce_H85R_R	TTGGTGCTCATATCCAGGGTC
Bce_F86T_F	CCGTATTCACACCGTGGACGTTAAGAAC
Bce_F86T_R	CCGTTGGTGCTCATATCC
Bce_D88A_F	GCGGTTAAGAACATCCTGGTGAAAATTCAG
Bce_D88A_R	CACAAAGTGAATACGGCCGTTGG
Bce_D88E_F	TCACTTTGTGGAAGTTAAGAACATCCTG
Bce_D88N_F	TCACTTTGTGAACGTTAAGAACATCCTG
Bce_D88E/N_R	ATACGGCCGTTGGTGCTC
Bce_K90A_F	TGTGGACGTTGCGAACATCCTGG
Bce_K90A_R	AAGTGAATACGGCCGTTG
Bce_H153A_F	CGTGCTGTTTGCCAAACTGCTGTTC
Bce_H153E_F	CGTGCTGTTTGAAAAACTGCTGTTC
Bce_H153N_F	CGTGCTGTTTAACAAACTGCTGT
Bce_H153A/E/N_R	AAGCTCTTCTCGAAGTTAATC
Bce_H153D_F	CGTGCTGTTTGACAAACTGCTGT
Bce_H153Q_F	CGTGCTGTTTCAGAAACTGCTGT
Bce_H153R_F	CGTGCTGTTTCGCAAACTGCTGT
Bce_H153D/Q/R_R	AAGCTCTTCTCGAAGTTAATCG
Bce_F157A_F	CAAACTGCTGGCCAGCAACGATTAC
Bce_F157A_R	TGAAACAGCACGAAGCTC
Bce_N159D_F	GCTGTTCAGCGACGATTACTGGG
Bce_N159D_R	AGTTTGTGAAACAGCACGAAG
Bce_W162A_F	CAACGATTACGCCGTTTTTGACCCGGAAAAAG
Bce_W162A_R	CTGAACAGCAGTTTGTGAAAC

[a] Primer sequences are from the 5' end to the 3' end.

[b] The first two primers in the table were used to amplify the *lit* gene; NdeI and XhoI restriction sites are in bold and italics.

'F' and 'R' in the primer names refer to the forward and reverse amplification primers, respectively.

[c] Primers used for mutagenesis of the *lit* gene

Supplementary References

1. Olatunji, S. *et al.* Structures of lipoprotein signal peptidase II from Staphylococcus aureus complexed with antibiotics globomycin and myxovirescin. *Nat. Commun.* **11**, 140 (2020).
2. Reichel, F. *et al.* Stereochemical dependence of the self-assembly of the immunoadjuvants Pam3Cys and Pam3Cys-Ser. *J. Am. Chem. Soc.* **121**, 7989–7997 (1999).
3. Arai, Y., Inuki, S. & Fujimoto, Y. Site-specific effect of polar functional group-modification in lipids of TLR2 ligands for modulating the ligand immunostimulatory activity. *Bioorg. Med. Chem. Lett.* **28**, 1638–1641 (2018).
4. Caffrey, M. On the Mechanism of Membrane Protein Crystallization in Lipidic Mesophases. *Cryst. Growth Des.* **8**, 4244–4254 (2008).
5. Kumar, S., Stecher, G., Li, M., Knyaz, C. & Tamura, K. MEGA X: Molecular Evolutionary Genetics Analysis across Computing Platforms. *Mol. Biol. Evol.* **35**, 1547–1549 (2018).
6. Saitou, N. & Nei, M. The neighbor-joining method: a new method for reconstructing phylogenetic trees. *Mol. Biol. Evol.* **4**, 406–425 (1987).
7. Miehlich, B., Savin, A., Stoll, H. & Preuss, H. Results obtained with the correlation energy density functionals of becke and Lee, Yang and Parr. *Chem. Phys. Lett.* **157**, 200–206 (1989).
8. Madeira, F. *et al.* The EMBL-EBI search and sequence analysis tools APIs in 2019. *Nucleic Acids Res.* **47**, W636–W641 (2019).
9. The UniProt Consortium. UniProt: a worldwide hub of protein knowledge. *Nucleic Acids Res.* **47**, D506–D515 (2018).
10. Landau, M. *et al.* ConSurf 2005: the projection of evolutionary conservation scores of residues on protein structures. *Nucleic Acids Res.* **33**, W299–W302 (2005).
11. Ashkenazy, H. *et al.* ConSurf 2016: an improved methodology to estimate and visualize evolutionary conservation in macromolecules. *Nucleic Acids Res.* **44**, W344–W350 (2016).
