# Supplementary material for: Structural basis of the membrane intramolecular transacylase reaction responsible for lyso-form lipoprotein synthesis
Source: Nat Commun. 2021 Jul 12;12:4254. doi: 10.1038/s41467-021-24475-0 (PMC8275575; doi:10.1038/s41467-021-24475-0)
Supplement: Supplementary file 3 — Supplementary Dataset 1 [file 41467_2021_24475_MOESM3_ESM.pdf]

Reactant

-1961.804707488701 a.u

C	37.133758	46.751541	70.541828
H	36.561306	47.482607	71.131301
H	37.856245	46.275532	71.217700
C	37.886493	47.406836	69.432359
N	37.293885	48.107407	68.393809
H	36.261983	48.215100	68.267273
C	38.201761	48.381809	67.456169
H	38.009281	48.908959	66.529082
N	39.379687	47.902446	67.871617
H	40.251195	47.801333	67.336585
C	39.210282	47.295550	69.095802
H	40.029998	46.812596	69.610760
C	40.544080	55.428007	64.484048
H	40.934381	56.457876	64.483131
H	39.812572	55.374262	65.302577
C	41.644103	54.443354	64.778767
N	42.979455	54.784080	64.852852
H	43.356264	55.697114	64.613269
C	43.699886	53.704545	65.238311
H	44.780857	53.726751	65.397842
N	42.890926	52.664980	65.407698
H	43.487786	51.284177	65.902918
C	41.615395	53.109365	65.125860
H	40.743234	52.467558	65.233508
N	44.022371	50.433457	66.379983
C	42.594058	46.566292	66.143277
C	42.260708	47.118447	64.782365
C	42.654063	46.188535	63.629876
C	42.150723	46.712726	62.284673
H	42.604209	47.698165	62.082330
H	41.055989	46.859371	62.320316
H	43.751939	46.112686	63.627304
H	42.278334	45.174509	63.838900
H	42.789390	48.080718	64.663498
H	41.181226	47.334412	64.775523
O	41.822754	46.621150	67.099097
H	45.036430	50.607913	66.187223
C	43.842246	50.464253	67.847066
H	42.989159	51.116524	68.060800
C	43.593512	49.103418	68.493114
S	45.055512	48.014457	68.253854
C	44.332678	46.367643	68.609244
C	44.403063	45.471966	67.371817
C	43.938527	44.028291	67.559044
O	44.677765	43.167701	66.684865
C	44.165362	42.849426	65.476955
C	45.108470	41.944504	64.710419
C	45.145068	42.186624	63.194110
H	45.703231	41.370780	62.711663
H	44.118873	42.171524	62.791749
H	44.753218	40.916326	64.911097
H	46.106232	42.006157	65.169250
O	43.080340	43.222426	65.094863
H	44.183028	43.692689	68.576599
H	42.861755	43.916850	67.374341
O	43.800141	46.039633	66.181818
H	43.830445	49.536855	65.922497
H	45.458250	45.411899	67.080016
H	43.317316	46.478678	69.006625
H	44.951035	45.898572	69.389920
H	43.410249	49.259960	69.566593
H	42.700475	48.631637	68.057992
C	45.124376	51.120623	68.396702
O	46.169852	50.978918	67.783859
N	44.958682	51.810459	69.528979
H	44.040186	51.838124	69.980765
C	46.013236	52.666846	70.039246
H	46.988082	52.287739	69.699232
H	36.434616	46.002630	70.169807
H	40.029835	55.238590	63.541848
H	42.380130	46.045723	61.453681
H	45.905236	53.689856	69.678892
H	45.959445	52.693177	71.127584
H	45.620920	43.121726	62.898785

TS1

-1961.802534863923 a.u

C	37.123595	46.717853	70.562748
H	36.551548	47.451139	71.150076
H	37.839832	46.237183	71.241937
C	37.887658	47.370769	69.460348
N	37.308300	48.082808	68.422309
H	36.277908	48.195826	68.287606
C	38.225918	48.354941	67.493320
H	38.045572	48.890352	66.569062
N	39.396262	47.861186	67.913433
H	40.270235	47.751681	67.383691
C	39.212306	47.247583	69.131679
H	40.023027	46.750321	69.646807
C	40.601036	55.453042	64.530934

H	40.997610	56.479826	64.510789
H	39.878428	55.416246	65.357836
C	41.698793	54.467175	64.831611
N	43.026646	54.819062	64.987464
H	43.403983	55.747259	64.819949
C	43.745341	53.741910	65.368636
H	44.816450	53.758118	65.582511
N	42.936229	52.691416	65.454862
H	43.352296	51.459963	65.839323
C	41.666360	53.122530	65.126834
H	40.805216	52.459052	65.161049
N	43.797327	50.393502	66.302899
C	42.591319	46.501768	66.179065
C	42.245889	47.047841	64.820334
C	42.636019	46.115872	63.669915
C	42.131965	46.638914	62.326064
H	42.575593	47.630023	62.129472
H	41.035483	46.772677	62.360734
H	43.733526	46.041672	63.668214
H	42.264603	45.101194	63.881761
H	42.777734	48.006198	64.690813
H	41.166918	47.265050	64.818717
O	41.827779	46.559646	67.140594
H	44.795745	50.397085	66.019069
C	43.728148	50.428585	67.771907
H	42.878921	51.060466	68.051734
C	43.547717	49.076970	68.465660
S	45.021544	48.010665	68.205188
C	44.345656	46.354969	68.621408
C	44.415592	45.434137	67.401680
C	43.973682	43.984057	67.598782
O	44.728923	43.136989	66.723706
C	44.238491	42.824873	65.504394
C	45.219696	41.962862	64.734676
C	45.243395	42.196001	63.216322
H	45.832520	41.398453	62.740416
H	44.218193	42.134445	62.815635
H	44.913988	40.920062	64.941521
H	46.214551	42.072083	65.191201
O	43.145872	43.169054	65.117447
H	44.226583	43.654867	68.616362
H	42.898469	43.853372	67.416357
O	43.797508	45.976188	66.207577
H	43.380206	49.563815	65.880747
H	45.469777	45.380686	67.105981
H	43.335673	46.453078	69.035448
H	44.989555	45.918802	69.400488
H	43.400862	49.255719	69.541475
H	42.650601	48.572607	68.078701
C	45.015405	51.132752	68.240565
O	46.037286	51.031909	67.580699
N	44.874513	51.807018	69.387443
H	43.973100	51.792702	69.874119
C	45.915063	52.675934	69.898705
H	46.888843	52.366042	69.491893
H	36.423230	45.972799	70.185322
H	40.074080	55.254142	63.597755
H	42.371577	45.975672	61.494942
H	45.741529	53.713541	69.613531
H	45.909682	52.640034	70.988085
H	45.677484	43.147447	62.909101

IM1

-1961.814310953324 a.u

C	37.042182	46.617691	70.576457
H	36.461191	47.364954	71.135891
H	37.733413	46.141951	71.284330
C	37.851059	47.245768	69.492649
N	37.325442	47.976507	68.439795
H	36.303364	48.111572	68.273983
C	38.287415	48.233102	67.547701
H	38.159031	48.780528	66.621493
N	39.426825	47.707087	68.005742
H	40.331995	47.573993	67.522270
C	39.182316	47.088891	69.208374
H	39.959848	46.562070	69.745048
C	40.567011	55.910069	64.502410
H	40.735474	56.994337	64.390639
H	39.901771	55.782261	65.368969
C	41.880360	55.236978	64.783408
N	42.853510	55.874126	65.543988
H	42.732908	56.758429	66.027879
C	43.931766	55.093873	65.682211
H	44.854541	55.313537	66.216839
N	43.683692	53.965333	65.033674
H	44.355274	53.180327	65.019674
C	42.424376	54.016195	64.467393
H	42.024518	53.193761	63.881970
N	43.827040	48.853625	66.856060
C	42.561792	46.407825	66.465828
C	42.230926	46.932166	65.094175
C	42.599380	45.966170	63.967552
C	42.150367	46.499660	62.609211

H	42.598036	47.494509	62.437566
H	41.053585	46.636135	62.606212
H	43.690811	45.830607	63.993054
H	42.180888	44.968684	64.167807
H	42.765902	47.883405	64.971157
H	41.152255	47.158313	65.090503
O	41.752536	46.391103	67.390479
H	43.954860	49.532338	66.100723
C	43.767645	49.531447	68.131765
H	42.862780	50.160136	68.163839
C	43.627837	48.525341	69.285796
S	44.990236	47.291053	69.329937
C	44.109956	45.717795	69.050659
C	44.294573	45.129146	67.652280
C	43.846890	43.673277	67.518113
O	44.723050	42.994784	66.610288
C	44.314426	42.690521	65.363441
C	45.411466	41.966796	64.609671
C	45.445866	42.229293	63.099246
H	46.080386	41.473279	62.613933
H	44.430302	42.120544	62.684108
H	45.216041	40.894026	64.794573
H	46.373510	42.176237	65.100780
O	43.205217	42.923992	64.941834
H	43.958822	43.157442	68.482355
H	42.805293	43.594991	67.176466
O	43.775435	45.886621	66.530045
H	44.700704	48.319917	66.845861
H	45.372377	45.145301	67.450840
H	43.059040	45.830232	69.337358
H	44.572465	44.998053	69.744934
H	43.622018	49.038268	70.256034
H	42.683340	47.975392	69.188241
C	44.973966	50.474255	68.338234
O	45.933916	50.446305	67.583681
N	44.853031	51.294557	69.402337
H	43.986883	51.274065	69.949379
C	45.875139	52.229576	69.811740
H	46.847593	51.928298	69.393105
H	36.340098	45.876301	70.195030
H	40.048931	55.555283	63.611462
H	42.419872	45.846800	61.779026
H	45.675003	53.252097	69.491639
H	45.898317	52.277235	70.900435
H	45.830071	43.209664	62.817598

TS2

-1961.809112541907 a.u

C	36.932446	46.592118	70.603360
H	36.331509	47.330381	71.153285
H	37.611225	46.121410	71.327468
C	37.768836	47.230507	69.549794
N	37.288736	47.966663	68.480658
H	36.275048	48.091675	68.269143
C	38.301570	48.245562	67.646491
H	38.219853	48.808774	66.724940
N	39.419627	47.727632	68.153950
H	40.411810	47.592375	67.773394
C	39.112725	47.092667	69.329414
H	39.869670	46.562796	69.890986
C	40.600414	56.166734	64.663018
H	40.679171	57.262655	64.542896
H	39.935916	55.996553	65.524782
C	41.961924	55.636049	65.012002
N	42.516192	55.971920	66.241627
H	42.018096	56.374521	67.037987
C	43.766439	55.507232	66.330430
H	44.424924	55.599539	67.189885
N	44.047583	54.881224	65.196770
H	44.981319	54.444699	65.018308
C	42.948239	54.932415	64.362713
H	42.960799	54.498467	63.369930
N	43.876713	48.168238	67.120024
C	42.601806	46.763556	66.840300
C	42.049334	47.118669	65.448019
C	42.659823	46.313898	64.306553
C	42.110727	46.680520	62.925827
H	42.373503	47.723988	62.678088
H	41.007432	46.606910	62.909329
H	43.749646	46.468360	64.329409
H	42.510481	45.243721	64.515536
H	42.149691	48.199167	65.268521
H	40.968330	46.914829	65.514488
O	41.793749	46.811677	67.818715
H	44.012229	48.711818	66.252735
C	43.657065	49.037050	68.272582
H	42.674410	49.527595	68.171304
C	43.651080	48.232751	69.594597
S	44.925114	46.923304	69.670331
C	43.983329	45.423679	69.203118
C	44.138009	44.978539	67.747097
C	43.782803	43.502147	67.519889
O	44.719145	42.922585	66.599589
C	44.374182	42.681159	65.320598

C	45.522435	42.010996	64.589172
C	45.548212	42.233585	63.073164
H	46.222856	41.502385	62.604550
H	44.540048	42.064955	62.661133
H	45.401031	40.933720	64.807943
H	46.465000	42.296053	65.081597
O	43.284361	42.915666	64.852548
H	43.896501	42.926786	68.449546
H	42.758797	43.406413	67.135275
O	43.488252	45.729166	66.714324
H	44.774924	47.697061	67.277551
H	45.211796	45.065164	67.518126
H	42.940236	45.551533	69.508311
H	44.430351	44.630672	69.821751
H	43.847935	48.902419	70.441179
H	42.677074	47.766043	69.765043
C	44.760061	50.120840	68.278629
O	45.676974	50.082690	67.475387
N	44.630180	51.040450	69.258989
H	43.806470	51.023454	69.870855
C	45.699761	51.940752	69.628325
H	46.654898	51.569192	69.226515
H	36.245187	45.843757	70.208773
H	40.113180	55.765399	63.774424
H	42.513345	46.017856	62.159771
H	45.571753	52.962832	69.271908
H	45.727424	52.026255	70.714599
H	45.878995	43.227245	62.771022

IM2

-1961.809481599268 a.u

C	36.930330	46.611407	70.590201
H	36.323982	47.355660	71.125918
H	37.601237	46.148392	71.326852
C	37.778597	47.237980	69.538573
N	37.312333	47.964932	68.457808
H	36.301562	48.096587	68.239835
C	38.337680	48.226478	67.630094
H	38.265333	48.779420	66.701387
N	39.448531	47.708579	68.150039
H	40.477707	47.548119	67.796139
C	39.123648	47.090752	69.329560
H	39.872422	46.562210	69.903367
C	40.591225	56.160480	64.672510
H	40.670437	57.257011	64.558274
H	39.925938	55.985407	65.532804
C	41.952131	55.627358	65.019602
N	42.504620	55.953670	66.252367
H	42.005182	56.351128	67.050745
C	43.755024	55.489763	66.339041
H	44.412110	55.575248	67.200289
N	44.038068	54.872376	65.200863
H	44.972425	54.439019	65.019477
C	42.939437	54.928781	64.366199
H	42.953312	54.503024	63.369917
N	43.819391	48.073121	67.168044
C	42.666440	46.871612	66.893514
C	42.066432	47.171142	65.495934
C	42.686392	46.379653	64.349616
C	42.127183	46.732915	62.968674
H	42.387120	47.773879	62.707121
H	41.024071	46.658422	62.959197
H	43.774687	46.547466	64.361940
H	42.554093	45.308702	64.562778
H	42.108079	48.254846	65.299338
H	41.000645	46.911732	65.588554
O	41.807330	46.885062	67.866172
H	44.004996	48.609306	66.299845
C	43.592541	48.974196	68.313052
H	42.604215	49.445062	68.202226
C	43.620815	48.204193	69.654757
S	44.903952	46.905845	69.721462
C	43.968525	45.408144	69.226548
C	44.140939	44.992048	67.762981
C	43.805264	43.513849	67.518492
O	44.744904	42.952236	66.589972
C	44.395122	42.707903	65.313425
C	45.535431	42.022780	64.583245
C	45.555813	42.234741	63.066114
H	46.230035	41.503719	62.597164
H	44.546056	42.061576	62.660137
H	45.404090	40.948347	64.809949
H	46.483528	42.300735	65.069179
O	43.307082	42.948425	64.844328
H	43.926981	42.927633	68.440301
H	42.781477	43.416135	67.134363
O	43.468838	45.737347	66.746079
H	44.711456	47.597254	67.367162
H	45.218084	45.093145	67.547473
H	42.920877	45.538465	69.514245
H	44.403835	44.607033	69.842698
H	43.839007	48.901405	70.473793
H	42.651636	47.742275	69.858168
C	44.703612	50.047347	68.269969

O	45.625339	49.958543	67.476426
N	44.578108	51.004560	69.213362
H	43.756169	51.007243	69.828735
C	45.665232	51.888519	69.578597
H	46.613711	51.499158	69.177962
H	36.246270	45.859128	70.197512
H	40.105463	55.763407	63.781199
H	42.525700	46.062349	62.207364
H	45.552650	52.910912	69.217896
H	45.695285	51.978191	70.664471
H	45.880789	43.228581	62.758314

TS3
-1961.774876601099 a.u

C	36.741266	46.586106	70.672489
H	36.124311	47.265315	71.279524
H	37.473988	46.120909	71.345688
C	37.488072	47.315648	69.612008
N	36.901520	48.067355	68.607727
H	35.873095	48.130248	68.442475
C	37.834376	48.443369	67.727122
H	37.659139	49.042708	66.841907
N	39.008723	47.968190	68.144983
H	39.939034	47.958227	67.684517
C	38.822726	47.265880	69.309854
H	39.642694	46.756697	69.800044
C	40.589504	56.103074	64.718606
H	40.657681	57.200367	64.604996
H	39.921544	55.921648	65.575521
C	41.954046	55.584934	65.071795
N	42.505510	55.929168	66.300731
H	42.005372	56.333640	67.094875
C	43.760734	55.477270	66.388829
H	44.419785	55.575946	67.247360
N	44.046331	54.852160	65.255937
H	44.983741	54.426917	65.072856
C	42.946077	54.888514	64.423818
H	42.962162	54.450706	63.432812
N	43.538878	48.284334	67.063344
C	42.349007	47.434853	66.742441
C	41.899656	47.447430	65.292055
C	42.568346	46.496397	64.302635
C	42.070182	46.721020	62.873613
H	42.343441	47.736247	62.533350
H	40.967422	46.651108	62.829318
H	43.655177	46.635840	64.362251
H	42.393996	45.462669	64.629328
H	42.011418	48.486495	64.935429
H	40.818751	47.238357	65.336611
O	41.527789	47.256640	67.654614
H	43.873495	48.852908	66.261566
C	43.494315	49.053166	68.323691
H	42.530960	49.586570	68.372739
C	43.591208	48.168837	69.588910
S	44.868156	46.864304	69.549534
C	43.887212	45.392199	69.055992
C	44.101677	45.006126	67.581048
C	43.416426	43.671371	67.222367
O	44.319028	42.879125	66.430719
C	44.176859	42.792485	65.100140
C	45.344027	42.028041	64.498613
C	45.529951	42.231313	62.993144
H	46.231564	41.484157	62.595371
H	44.564259	42.079618	62.484693
H	45.149864	40.962873	64.723581
H	46.247607	42.273372	65.079033
O	43.231528	43.226653	64.481211
H	43.217258	43.063568	68.118660
H	42.480410	43.839917	66.672465
O	43.825704	46.023505	66.683692
H	44.207020	47.379608	67.080097
H	45.182684	44.781652	67.493637
H	42.835536	45.607584	69.293431
H	44.222040	44.570620	69.708323
H	43.827905	48.815931	70.444237
H	42.624385	47.701443	69.801158
C	44.625539	50.099067	68.262860
O	45.471068	50.072723	67.386084
N	44.574994	50.987488	69.278102
H	43.776674	50.974823	69.923182
C	45.666447	51.873524	69.624610
H	46.615111	51.482291	69.226347
H	36.081527	45.819789	70.265587
H	40.111015	55.702196	63.825067
H	42.486638	45.991857	62.178664
H	45.553288	52.893078	69.256139
H	45.694510	51.974302	70.709563
H	45.905932	43.216317	62.716607

P
-1961.851025880270 a.u

C	36.462159	46.669548	70.652740
H	35.812904	47.245992	71.331313

H	37.287987	46.267521	71.256333
C	37.032832	47.532683	69.583054
N	36.284967	48.203315	68.628896
H	35.253585	48.113498	68.504422
C	37.103062	48.775747	67.740002
H	36.791477	49.370899	66.890697
N	38.356507	48.505007	68.100035
H	39.260527	48.665640	67.601150
C	38.341379	47.741829	69.241275
H	39.256569	47.398992	69.706488
C	40.618411	56.056319	64.708697
H	40.691560	57.153402	64.595354
H	39.956123	55.877016	65.570295
C	41.981272	55.527808	65.053031
N	42.546902	55.871980	66.275622
H	42.061740	56.296912	67.068610
C	43.794188	55.397487	66.359616
H	44.460980	55.494164	67.212340
N	44.060704	54.755874	65.231047
H	44.989565	54.310844	65.047241
C	42.956387	54.808672	64.404086
H	42.956599	54.361652	63.416955
N	43.032464	48.752386	67.037342
C	41.809766	48.390336	66.656143
C	41.577919	48.171506	65.181474
C	42.186968	46.881410	64.613338
C	41.971276	46.848942	63.100311
H	42.384477	47.775322	62.663500
H	40.886073	46.851145	62.891677
H	43.255622	46.831320	64.861788
H	41.728711	46.004502	65.098492
H	42.003520	49.027534	64.632934
H	40.486625	48.170164	65.027707
O	40.882612	48.248793	67.491912
H	43.786314	48.861609	66.340892
C	43.299989	49.220632	68.383434
H	42.429618	49.808482	68.704133
C	43.460808	48.117636	69.445543
S	44.657107	46.804532	69.027015
C	43.502024	45.513892	68.427488
C	44.258206	44.594031	67.478149
C	43.428754	43.402010	67.031098
O	44.285666	42.527276	66.295666
C	44.215855	42.528694	64.948914
C	45.413302	41.813335	64.359732
C	45.607745	42.051491	62.861831
H	46.338390	41.340766	62.452040

H	44.652479	41.881318	62.340715
H	45.254072	40.740787	64.573687
H	46.296244	42.087918	64.959215
O	43.296622	43.001705	64.324018
H	43.068751	42.840674	67.905790
H	42.582526	43.716808	66.403140
O	44.663331	45.292197	66.320303
H	45.039520	46.137915	66.621446
H	45.130756	44.179655	68.019298
H	42.688860	46.001516	67.871730
H	43.097706	44.949240	69.283989
H	43.788302	48.579070	70.386827
H	42.490006	47.649040	69.651342
C	44.534236	50.135283	68.339046
O	45.389492	50.020717	67.476951
N	44.561032	51.010162	69.364358
H	43.760748	51.048442	70.005972
C	45.689564	51.852116	69.697421
H	46.621117	51.431564	69.284415
H	35.865699	45.848294	70.255443
H	40.131013	55.658465	63.818629
H	42.429177	46.004152	62.585798
H	45.600209	52.871881	69.323037
H	45.730511	51.952594	70.781992
H	45.949978	43.056341	62.614403
