# Supplementary material for: Structural basis of the membrane intramolecular transacylase reaction responsible for lyso-form lipoprotein synthesis
Source: Nat Commun. 2021 Jul 12;12:4254. doi: 10.1038/s41467-021-24475-0 (PMC8275575; doi:10.1038/s41467-021-24475-0)
Supplement: Supplementary file 11 — Description of additional supplementary files [file 41467_2021_24475_MOESM11_ESM.docx]

Description of additional supplementary information

Title: Supplementary Movie 1.

Description: Dynamics of the empty Lit structure revealed by MD simulations in a POPC/POPG membrane. View is from the extracytoplasmic space into the putative binding pocket. The protein is in cartoon representation coloured to match Fig. 2. The aromatic residues of the active site are shown as sticks with cyan carbon atoms. Lipids and ions are in space-filled representation. The movie shows the enhanced mobility of H4 and H5 (orange) and the extracytoplasmic half of M3 at the M3/M4 interface (yellow/red).

Title: Supplementary Movie 2.

Description: Flexibility in parts of Lit detected by principle component (PC1) analysis of MD simulations trajectories suggest a route into and out of the active site. The movie shows inward and outward movements of the extracellular half of M3 and H4 from the helical core.

Title: Supplementary Movie 3.

Description: Flexibility in parts of Lit detected by principle component (PC2) analysis of MD simulations trajectories suggest a route into and out of the active site. The movie shows upward and downward movements of the two EGDs.

Title: Supplementary Movie 4.

Description: Flexibility in parts of Lit detected by principle component (PC3) analysis of MD simulations trajectories suggest a route into and out of the active site. The movie shows upward and outward movements of the two EGDs and outward movement of the extracellular half of M3.

Title: Supplementary Movie 5.

Description: Dynamics of the substrate in the Lit active site from MD simulations. The substrate is shown in thick stick representation. His85, Asp88, His153, Asn159 and Trp162 of Lit are shown as thin sticks. Water molecules at a distance of 5 Å from these residues are shown in space-filling representation. The Lit is in grey cartoon representation. The α-ammonium group of the substrate is close to His153 while the carbonyl group of the sn-2 chain of the substrate is proximal to His85. The water molecule between His85 and Asp88 observed in the crystal structure remains in place during the MD simulations with a percentage residence time of 77±3%.

Title: Supplementary Movie 6.

Description: Dynamics of the product in the Lit active site from MD simulations. The product is shown in thick stick representation. His85, Asp88, His153, Asn159 and Trp162 of Lit are shown as thin sticks. Water molecules at the distance of 5 Å from these residues are shown in space-filling representation. The Lit is in grey cartoon representation. The carbonyl group of the sn-2 chain (now in an amide linkage) is close to His85. The protonated His153 moves away from the active site to where it engages in cationπ interaction with Trp162. The water molecule between His85 and Asp88 observed in the crystal structure remains in place during the MD simulations with a percentage residence time of 76±5%.

Title: Supplementary Movie 7.

Description: MD simulations show that the His153 rotates back toward the active site following deprotonation by equilibration with bulk water. The conformation of Lit from the product-Lit complex with the protonated His153 facing the aqueous environment (in cationπ interaction with Trp162) was used as a starting structure for this MD simulations of Lit with His153 in its neutral, deprotonated form. During the MD simulations, the uncharged His153 side chain moves to its original position facing into the active site for another reaction round. His85, His153 and Trp162 are shown in stick representation.
