# Supplementary material for: Structural basis of the membrane intramolecular transacylase reaction responsible for lyso-form lipoprotein synthesis
Source: Nat Commun. 2021 Jul 12;12:4254. doi: 10.1038/s41467-021-24475-0 (PMC8275575; doi:10.1038/s41467-021-24475-0)
Supplement: Supplementary file 12 — Reporting Summary [file 41467_2021_24475_MOESM12_ESM.pdf]

nature research

Corresponding author(s): Martin Caffrey
Last updated by author(s): 24 May 2021

Reporting Summary

Nature Research wishes to improve the reproducibility of the work that we publish. This form provides structure for consistency and transparency in reporting. For further information on Nature Research policies, see our Editorial Policies and the Editorial Policy Checklist.

Statistics

For all statistical analyses, confirm that the following items are present in the figure legend, table legend, main text, or Methods section.

n/a	Confirmed	
☐	☒	The exact sample size (*n*) for each experimental group/condition, given as a discrete number and unit of measurement
☐	☒	A statement on whether measurements were taken from distinct samples or whether the same sample was measured repeatedly
☒	☐	The statistical test(s) used AND whether they are one- or two-sided<br>*Only common tests should be described solely by name; describe more complex techniques in the Methods section.*
☒	☐	A description of all covariates tested
☒	☐	A description of any assumptions or corrections, such as tests of normality and adjustment for multiple comparisons
☐	☒	A full description of the statistical parameters including central tendency (e.g. means) or other basic estimates (e.g. regression coefficient) AND variation (e.g. standard deviation) or associated estimates of uncertainty (e.g. confidence intervals)
☒	☐	For null hypothesis testing, the test statistic (e.g. *F*, *t*, *r*) with confidence intervals, effect sizes, degrees of freedom and *P* value noted<br>*Give P values as exact values whenever suitable.*
☒	☐	For Bayesian analysis, information on the choice of priors and Markov chain Monte Carlo settings
☒	☐	For hierarchical and complex designs, identification of the appropriate level for tests and full reporting of outcomes
☒	☐	Estimates of effect sizes (e.g. Cohen's *d*, Pearson's *r*), indicating how they were calculated

Our web collection on statistics for biologists contains articles on many of the points above.

Software and code

Policy information about availability of computer code

Data collection

Se-methionine derivative X-ray diffraction data collection was performed using data acquisition software suites (DA+) from the Swiss Light Source PXI-X06SA beamline.

Data analysis

Crystallography data analysis:
- XDS/XSCALE (version Jan 26, 2018 BUILT=20180409, version Jan 26, 2018 BUILT=20180808, and version Mar 15, 2019 BUILT=20190315)
- autoPROC/STARANISO (STARANISO OpenMP version: 2.2.1, 29-Oct-2018)
- HKL2MAP (version 0.4 c-beta)
- SHELXC/D/E (included in HKL2MAP version 0.4 c-beta)
- CRANK2 (CRANK2 version 2.0.253 in CCP4 7.0.077)
- Coot (version 0.8.9.1 EL)
- Phenix.refine (included in PHENIX dev_3494, and PHENIX version 1.17.1_3660)
- BUSTER (Version 2.10.3)
- PyMOL (Version 1.8.4.0 Open-Source)

TLC data analysis:
- ImageJ (version 1.52)
- GraphPad Prism (version 8)

NMR data analysis:
- MestReNova (version 6.0.2-5475)

MD and QM/MM simulations:
- Maestro version 2018-4 Amber version 2018 (Including Amber18 and AmberTools18)
- ChemShell suite version 2019

- VMD (version 1.9.3)
- NAMD (version 2.13)
- ORCA (version 4.2.0)
- DL_POLY MD (version DL_POLY_2)

Web servers:
- Expasy ProtParam (https://web.expasy.org/protparam/)
- HmmerWeb server (https://www.ebi.ac.uk/Tools/hmmer/search/jackhmmer)
- Clustal Omega (https://www.ebi.ac.uk/Tools/msa/clustalo/)
- Consurf (https://consurf.tau.ac.il)
- WebLogo3 (http://weblogo.threeplusone.com/create.cgi)
- Orientation of Protein in Membranes (OPM) server (https://opm.phar.umich.edu)
- CHARMM-GUI (https://www.charmm-gui.org)

For manuscripts utilizing custom algorithms or software that are central to the research but not yet described in published literature, software must be made available to editors and reviewers. We strongly encourage code deposition in a community repository (e.g. GitHub). See the Nature Research guidelines for submitting code & software for further information.

Data

Policy information about availability of data

All manuscripts must include a data availability statement. This statement should provide the following information, where applicable:

- Accession codes, unique identifiers, or web links for publicly available datasets
- A list of figures that have associated raw data
- A description of any restrictions on data availability

Source data are provided with this paper. Atomic coordinates and structure factors have been deposited in the Protein Data Bank (PDB) with accession codes 7B0O [https://doi.org/10.2210/pdb7B0O/pdb] (apo Lit wild-type, monoclinic P21 form), 7B0P [https://doi.org/10.2210/pdb7B0P/pdb] (apo Lit wild-type, orthorhombic P21212 form), 7B0Q [https://doi.org/10.2210/pdb7B0Q/pdb] (LitH85A, monoclinic C21 form), and 7B0R [https://doi.org/10.2210/pdb7B0R/pdb] (LitH85R, monoclinic C21 form). The source data underlying Fig. 3f and Supplementary Figs. 9 and 10 are provided as a Source Data file. The atomic coordinates of the QM region used in the QM/MM are available in .xyz file format. Other data supporting the findings of this manuscript are available from the corresponding author upon reasonable request.

Field-specific reporting

Please select the one below that is the best fit for your research. If you are not sure, read the appropriate sections before making your selection.

☒ Life sciences ☐ Behavioural & social sciences ☐ Ecological, evolutionary & environmental sciences

For a reference copy of the document with all sections, see nature.com/documents/nr-reporting-summary-flat.pdf

Life sciences study design

All studies must disclose on these points even when the disclosure is negative.

Sample size	TLC-based assays were performed at a minimum with technical duplicates. No statistical methods were used to determine sample size.
Data exclusions	No data was excluded.
Replication	Protein production and purification of the wild-type protein were conducted at least five times independently in the study.<br>Mutants proteins were produced once or twice, following a reproducible protocol described in the method section of the manuscript. All attempts to grow crystals of the wild-type or H85A and H85R mutants proteins and replicate enzyme assays were successful.
Randomization	Samples were not allocated into groups. Randomization is not relevant to this study.
Blinding	Blinding is not relevant to this study as no grouping of samples was performed.

Reporting for specific materials, systems and methods

We require information from authors about some types of materials, experimental systems and methods used in many studies. Here, indicate whether each material, system or method listed is relevant to your study. If you are not sure if a list item applies to your research, read the appropriate section before selecting a response.

Materials & experimental systems

n/a	Involved in the study
☒	☐ Antibodies
☒	☐ Eukaryotic cell lines
☒	☐ Palaeontology and archaeology
☒	☐ Animals and other organisms
☒	☐ Human research participants
☒	☐ Clinical data
☒	☐ Dual use research of concern

Methods

n/a	Involved in the study
☒	☐ ChIP-seq
☒	☐ Flow cytometry
☒	☐ MRI-based neuroimaging
